# Incidence, risk factors, and epidemiological trends of tracheal cancer: a global analysis

**DOI:** 10.1186/s12943-024-02188-4

**Published:** 2024-12-18

**Authors:** Junjie Huang, Mingtao Chen, Lin Zhang, Xu Lin, Don Eliseo Lucero-Prisno, Claire Chenwen Zhong, Wanghong Xu, Zhi-Jie Zheng, Mellissa Withers, Martin C. S. Wong, N. C. D. Global Health Working Group, Association of Pacific Rim Universities (APRU)

**Affiliations:** 1 The Jockey Club School of Public Health and Primary Care, Faculty of Medicine, Chinese University of Hong Kong, Hong Kong SAR, China; 2 Centre for Health Education and Health Promotion, Faculty of Medicine, The Chinese University of Hong Kong, Hong Kong SAR, China; 3 The School of Public Health and Preventive Medicine, Monash University, Victoria, Australia; 4 Department of Thoracic Surgery, School of Medicine, The First Affiliated Hospital, Zhejiang University, Hangzhou, Zhejiang China; 5 Department of Global Health and Development, London School of Hygiene and Tropical Medicine, London, UK; 6 School of Public Health, Fudan University, Shanghai, China; 7 Department of Global Health, School of Public Health, Peking University, Beijing, China; 8 Department of Population and Health Sciences, Institute for Global Health, University of Southern California, Los Angeles, USA; 9 School of Public Health, The Chinese Academy of Medical Sciences and Peking Union Medical College, Beijing, China

**Keywords:** Tracheal cancer, Epidemiology, Risk factor, Cancer registry

## Abstract

**Background:**

Tracheal cancer is a rare malignancy with limited research but high mortality rates. This study aims to analyse recent data to understand the global burden, trends, and risk factors for tracheal cancer, facilitating improved prevention and treatment strategies.

**Methods:**

We conducted a study on tracheal cancer using data from the Global Cancer Observatory and the Cancer Incidence in Five Continents databases. We collected information on the incidence of tracheal cancer, risk factors, and the Human Development Index (HDI) at the country level. The univariate linear regression was used to explore the relationship between tracheal cancer and the various risk factors. We utilised joinpoint regression analysis to calculate the Average Annual Percentage Change (AAPC) in tracheal cancer incidence.

**Results:**

The global age-standardised rate of incidence of tracheal cancer was 2.9 per 10 million (3,472 cases in total) in 2022, with the highest regional incidence observed in Central and Eastern Europe (ASR = 9.0) and the highest national incidence in Hungary (12.5). Higher incidence was found among the males (3.8) than females (2.0); among the older adults aged 50-74 (11.9) than the younger population aged 15-49 (1.2). A higher tracheal cancer incidence ratio was associated with higher levels of smoking, alcohol drinking, diabetes, lipid disorders, and HDI. Despite the overall decreasing trends for all population groups (highest decrease in Thailand; AAPC: -15.06, 95% CI: -21.76 to -7.78, *p* = 0.002), there was an increase in some female populations (highest increase in Colombia, AAPC: 19.28, 95% CI: 16.48 to 22.15, *p* < 0.001) and younger populations (highest increase in Ireland; AAPC: 29.84, 95% CI: 25.74 to 34.06, *p* < 0.001).

**Conclusion:**

This study provides a comprehensive analysis of tracheal cancer, focusing on risk factors and population-level trends. There has been an overall decreasing trend in the incidence of tracheal cancer, particularly among males and older adults, while the decline is less pronounced in females and younger individuals. Further research is needed to explore the underlying drivers of these epidemiological trends.

**Supplementary Information:**

The online version contains supplementary material available at 10.1186/s12943-024-02188-4.

## Background

Tracheal cancer is a rare malignancy that accounts for only 0.1–0.4% of all malignant diseases [1]. However, the primary diagnosis often presents with an advanced stage due to the delayed diagnosis [2]. The 5-year survival rate of tracheal cancer was 27.1% [1]. Despite its rarity, it can be categorised into two major types, squamous cell carcinoma (SCC) and adenoid cystic carcinoma (ACC). One Demark study in 2001 discovered that SCC was the most common subtype with the lowest survival rate (7%) when compared with ACC (50%) [3]. As it is a rare type of cancer, the treatments available were also limited to radiology and surgical removal of the malignancy.

The trachea is composed of various types of cells and features functional components, including the mucous layer, the airway surface liquid, and cilia. These structures work together to clear inhaled pathogens and particles, preventing their entry into the lungs [4]. Although the trachea was protected with such a well-structured mechanism, it shares some of the common risk factors of lung cancer [5]. Recent research has discovered SCC was more common among smokers, while ACC was more common among non-smokers [6]. Moreover, the majority of tracheal cancers occur in men [6]. However, due to its rarity, there has been limited research on the risk factors associated with this type of cancer.

Due to the rarity of primary tracheal cancer, it is often not studied as a distinct entity but rather grouped with bronchial and lung cancers. Recent studies have been limited to specific countries, and the available data is relatively outdated [3, 7, 8]. Moreover, the limited number of cases restricts opportunities to investigate its risk factors. This study aims to contribute to pioneering efforts in addressing the knowledge gap by analysing the most recent data to provide an updated assessment of the global disease burden and trends by country, gender, and age group. Additionally, risk factors will be examined at the country level to offer insights for primary prevention.

## Methods

### Data sources

The incidence of tracheal cancer was determined by the International Classification of Diseases, Tenth Revision (ICD-10) C33: malignant neoplasm of trachea using data of 10-year cancer occurrence in 108 countries from the Cancer Incidence in Five Continents Plus (CI5 Plus) database [9]. The CI5 Plus database is a useful database for time trend analysis and comprehending changes across time as it provides the most recent and historical data set on cancer incidence and further categorising it into three categories: year, population, and region. By accessing the Global Cancer Observatory (GLOBOCAN) database, the incidence of tracheal cancer in 185 countries was estimated using proportion estimations (tracheal cancer out of lung and tracheal cancers) from the Cancer Incidence in Five Continents (CI5) [10]. GLOBALCAN, as an online data base, provides global incidence and mortality statistics for 26 different types of cancer. It is a database created by the International Association of Cancer Registries through cooperating with multiple population-based registries of cancer, the World Health Organisation, and publicly available online data. By using those data from the international or national cancer registry database, GLOBALCAN computed the cancer-related statistics data, such as the incidence-to-mortality ratio and trend analysis forecast [11]. As for the risk factor analysis, the Global Burden of Disease (GBD) database was used for the country-specific data. The variables for each country included the weighted prevalence of smoking, alcohol drinking, unhealthy diet, obesity, hypertension, diabetes, and lipid disorders. The GBD database aims to improve healthcare systems and enhance health equity by examining the health loss derived from specific illnesses, accidents, and risk factors [12]. It includes data on early death and disability caused by over 350 diseases and injuries and 80 risk factors examined by more than 7,000 researchers in 156 countries and regions. The United Nations and the World Bank were accessed for each country's human development index (HDI) and the gross domestic product (GDP) per person for each nation, respectively [13, 14].

### Statistical analysis

Risk variables such as HDI, GDP per capita, lifestyle, and metabolic risk factors were examined for their relationship with the incidence of tracheal cancer through conducting univariate linear regression analysis for each country by sex and age. In the regression analysis, beta coefficients (*β*) and the corresponding 95% confidence intervals (CI) were generated. *β* estimates represent the ratio of the change between the outcome variable (ASR of incidence) and the change in the predictor variable, expressed as a unit increase (risk factor). The significance of the result was defined as a p-value less than 0.05.

In this study, the joinpoint regression analysis was conducted for trend analysis of the tracheal cancer incidence using software developed by the SEER Program of the National Cancer Institute of the United States. To determine the temporal trend of tracheal cancer incidence, the Average Annual Percentage Change (AAPC) was calculated [15]. The current study selected the most recent 10-year data for the calculation, which followed the customary in cancer epidemiology research. The corresponding standard errors for the incidence data's logarithmic transformation had been generated. Afterward, for every distinct demographic group, the AAPC and the 95% CI were determined, and it was used to assess the accuracy of the trend. When the interval overlaps with 0, it represents a stable trend without a significant increase or decrease. Moreover, a positive AAPC represents an upward trend in tracheal cancer incidence, and vice versa. Changes in tracheal cancer incidence were evaluated in terms of subgroups of gender (male and female), age (all population: 0–85 + years, young population: 15–49 years, old population: 50–74 years), and geographic regions (Asia, Oceania, America, Europe, and Africa).

## Results

### Tracheal cancer incidence in 2022

Globally, there were a total of 3,474 tracheal cancer incidences with an ASR of 2.9 per 10,000,000 people in 2022 (Supplementary Table 1a). In subregions, Central and Eastern Europe (9.0), Western Europe (5.0), and Southern Europe (5.0) were found with the highest ASR, while the lowest ASR was in Northern Africa (1.0), South-Central Asia (2.0), and South-Eastern Asia (2.0). On a country level, Hungary (12.5), France, Guadeloupe (10.9), and Croatia (10.1) were observed with the highest ASR. Conversely, Sudan (0.23), Yemen (0.37), and Saudi Arabia (0.40) were observed to be the lowest in ASR.

### Tracheal cancer incidence by subgroup in 2022

#### Tracheal cancer incidence by sex groups

Globally, the ASR of male tracheal cancer incidence was 3.8 (2,223) per 10 million people in 2022, whereas the ASR of the female population was 2.0 (1,251). In terms of subregions, Central and Eastern Europe (14.0), Eastern Asia (6.0), and Western Europe (6.0) were observed with the highest male ASR in tracheal cancer incidence while Central and Eastern Europe (4.0), Southern Europe (3.0), and Western Europe (3.0) reported the highest incidence among females. Countries with the highest ASR were located in France, Guadeloupe (17.3), Belarus (17.1), and the Russian Federation (16.9) among the male populations, whereas Hungary (10.7), South Africa (7.6), and Poland (7.4) had the highest ASR among female populations.

#### Tracheal cancer incidence by age groups

**Fig. 1:**
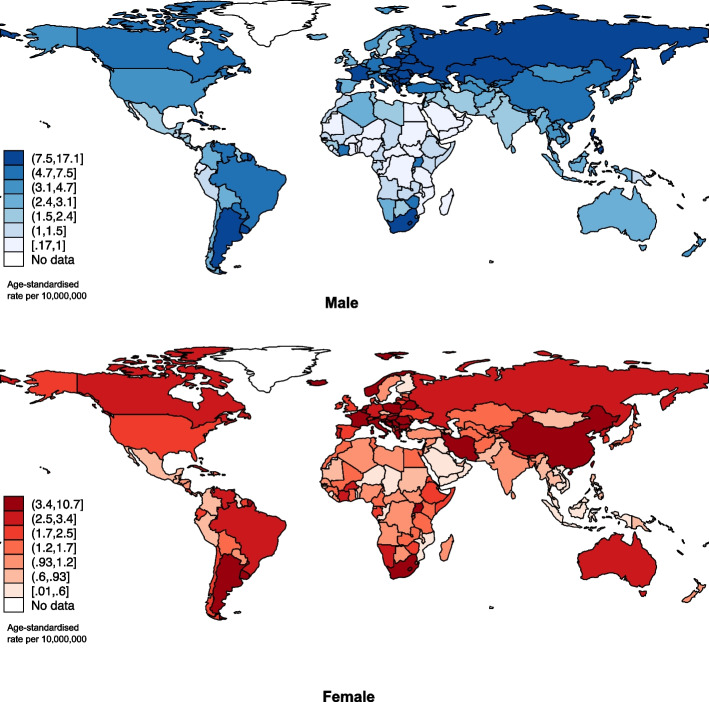
Global incidence of tracheal cancer by sex, all ages, in 2022

**Fig. 2:**
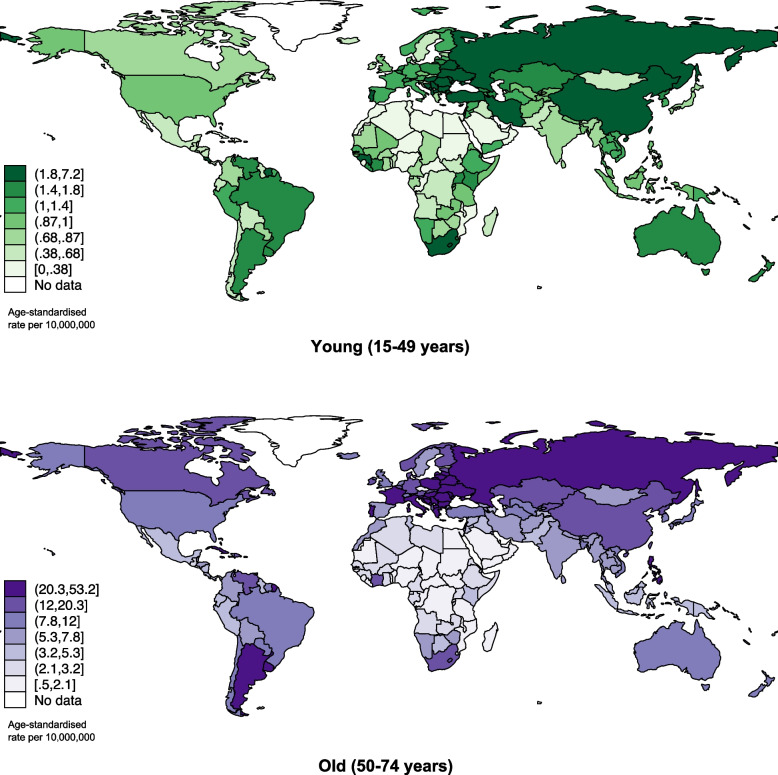
Global incidence of tracheal cancer by ages, all sexes, in 2022

Globally, the ASR of the old age group (11.9; 2,045 total new cases reported) was much higher than the young age group (1.2; 472 total new cases reported) in 2022. (Fig. 1; Supplementary Table 1a). By region, Central and Eastern Europe (3.0 vs. 33.0 for old) had the highest ASR in both the younger population and older population, while the ASR of the older population was considerably higher than the younger population for each region. On the country level, the highest ASR of the younger population was located in Brunei Darussalam (7.2), New Caledonia (4.5), and Romania (4.2). As for the older population, France, Guadeloupe (68.5), Hungary (53.2), and Croatia (46.3) were observed with the highest ASR. (Fig. 2; Supplementary Table 1b).

### Associations of risk factors with tracheal cancer incidence

Including all ages and sexes, the associated risk factors of tracheal cancer incidence included higher level of HDI (*β* = 0.643, 95%CI 0.391 to 0.894, *p* < 0.001) and GDP per capita (*β* = 0.226, 95%CI 0.030 to 0.422, *p* = 0.024), higher prevalence of smoking (*β* = 0.300, 95%CI 0.242 to 0.357, p < 0.001), alcohol drinking (*β* = 0.212, 95%CI 0.140 to 0.283, *p* < 0.001), unhealthy dietary (*β* = 0.052, 95%CI 0.016 to 0.088, *p* = 0.005), physical inactivity (*β* = 0.134, 95%CI 0.005 to 0.264, *p* = 0.042), obesity (*β* = 0.097, 95%CI 0.063 to 0.132, *p* < 0.001), hypertension (*β* = 0.120, 95%CI 0.075 to 0.164, *p* < 0.001), diabetes (*β* = 0.149, 95%CI 0.076 to 0.222, *p* < 0.001) and lipid disorder (*β* = 0.123, 95%CI 0.094 to 0.153, *p* < 0.001) (Supplementary Table 2).

### Associations of risk factors with tracheal cancer incidence by subgroup

**Fig. 3:**
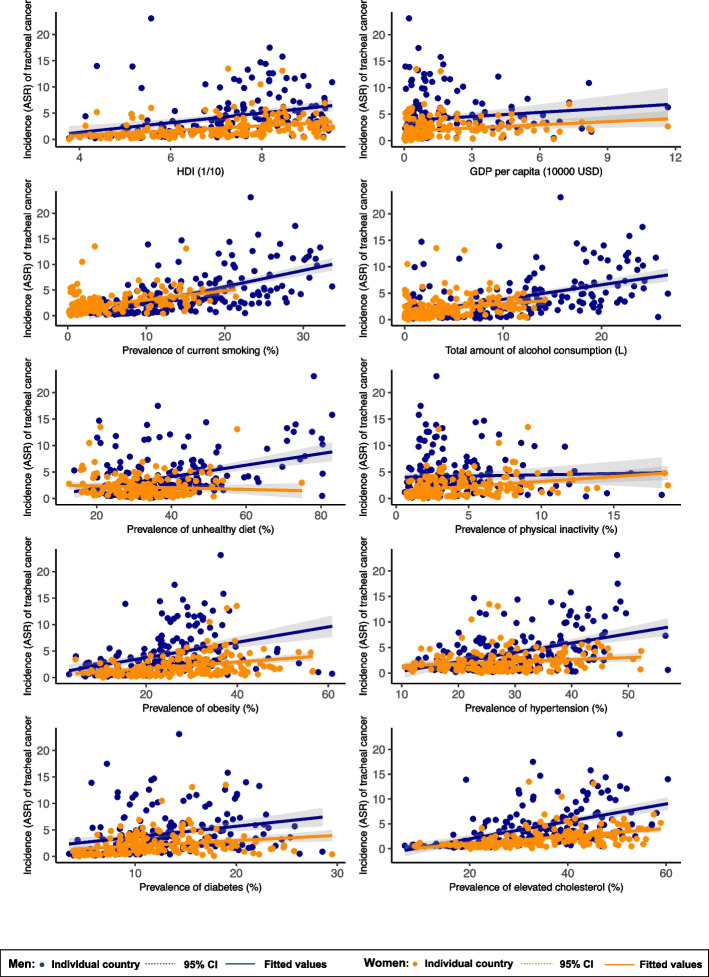
Association between risk factors and tracheal cancer by sex

Among the males, higher tracheal cancer incidence was associated with higher HDI (*β* = 0.923, 95%CI 0.546 to 1.300, *p* < 0.001) and higher prevalence of smoking (*β* = 0.319, 95%CI 0.257 to 0.381, *p* < 0.001), alcohol drinking *(β* = 0.275, 95%CI 0.202 to 0.348, *p* < 0.001), unhealthy dietary (*β* = 0.109, 95%CI 0.070 to 0.148, *p* < 0.001), obesity (*β* = 0.145, 95%CI 0.094 to 0.196, *p* < 0.001), hypertension (*β* = 0.183, 95%CI 0.123 to 0.244, *p* < 0.001), diabetes (*β* = 0.202, 95%CI 0.099 to 0.305, *p* < 0.001), and lipid disorder (*β* = 0.176, 95%CI 0.133 to 0.219, *p* < 0.001). As for the female population, the incidence of tracheal cancer was associated with higher HDI (*β* = 0.462, 95%CI 0.271 to 0652, *p* < 0.001), GDP per capita (*β* = 0.198, 95%CI 0.054 to 0.343, *p* = 0.007), higher prevalence of smoking (*β* = 0.176, 95%CI 0.122 to 0.230, *p* < 0.001), alcohol drinking *(β* = 0.139, 95%CI 0.059 to 0.220, *p* = 0.001), physical inactivity (*β* = 0.186, 95%CI 0.098 to 0.274, *p* < 0.001), obesity (*β* = 0.062, 95%CI 0.036 to 0.088, *p* < 0.001), hypertension (*β* = 0.039, 95%CI 0.005 to 0.074, p = 0.026), diabetes (*β* = 0.100, 95%CI 0.043 to 0.157, *p* = 0.001) and lipid disorder (*β* = 0.077, 95%CI 0.053 to 0.101, *p* < 0.001) (Fig. 3).

**Fig. 4:**
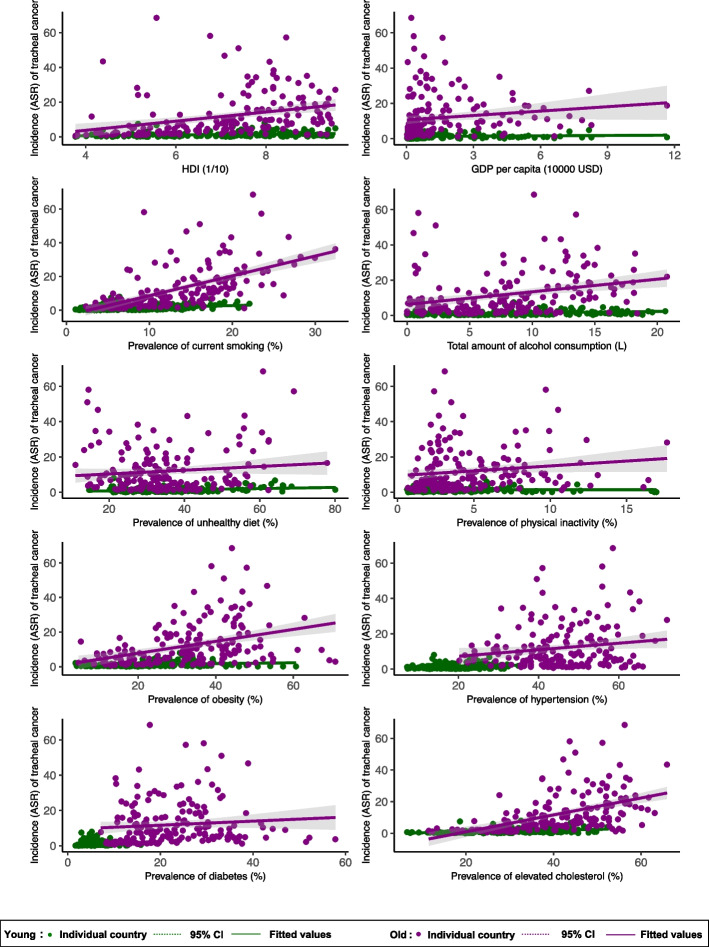
Association between risk factors and tracheal cancer by age

The associated risk factors of tracheal cancer incidence in younger population included higher HDI (*β* = 0.222, 95%CI 0.086 to 0.359, *p* = 0.002) and higher prevalence of smoking (*β* = 0.120, 95%CI 0.082 to 0.159, *p* < 0.001), alcohol drinking *(β* = 0.078, 95%CI 0.041 to 0.116, *p* < 0.001), obesity (*β* = 0.024, 95%CI 0.005 to 0.043, *p* = 0.015), hypertension (*β* = 0.048, 95%CI 0.012 to 0.084, *p* = 0.008), and lipid disorder (*β* = 0.054, 95%CI 0.035 to 0.073, *p* < 0.001). Among the older population, tracheal cancer incidence was associated with higher HDI (*β* = 2.629, 95%CI 1.470 to 3.788, p < 0.001), higher prevalence of smoking (*β* = 1.175, 95%CI 0.950 to 1.399, *p* < 0.001), alcohol drinking *(β* = 0.716, 95%CI 0.362 to 1.069, *p* < 0.001), obesity (*β* = 0.345, 95%CI 0.220 to 0.470, *p* < 0.001), hypertension (*β* = 0.185, 95%CI 0.014 to 0.356 *p* = 0.034), and lipid disorder (*β* = 0.534, 95%CI 0.397 to 0.671, *p* < 0.001) (Fig. 4; Supplementary Table 2).

### Temporal trend overall

The overall trend of tracheal cancer was decreasing, with six countries showing a decreasing trend and only one demonstrating an increasing trend (Supplementary Table 3c; Supplementary Fig. 1). Countries with the most significant trend decrease were Thailand (AAPC: -15.06, 95% CI: -21.76 to -7.78, *p* = 0.002), New Zealand (AAPC: -14.40, 95% CI: -21.92 to -6.15, *p* = 0.005), and Uganda (AAPC: -13.17, 95% CI: -24.36 to -0.34, *p* = 0.045). The only country with an increase was Kuwait (AAPC: 15.17, 95% CI: 0.34 to 32.20, *p* = 0.045). 34 countries have no significant increase or decrease during this period.

### Sex- and age- specific trends analysis

**Fig. 5:**
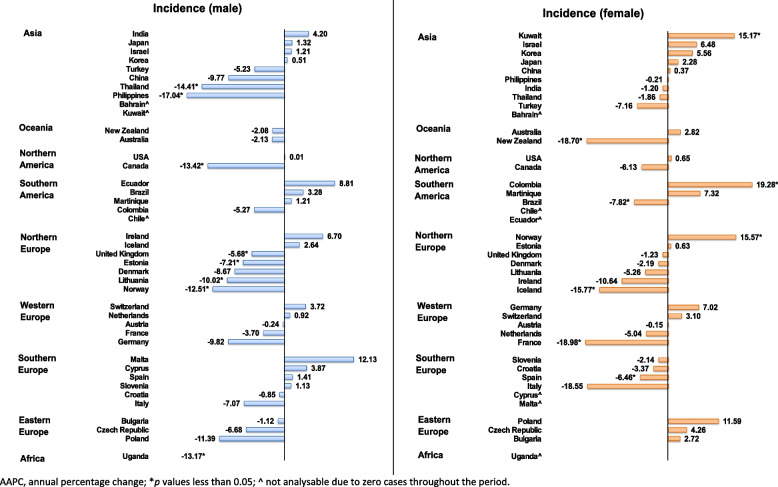
The AAPC of the incidence of tracheal cancer by sex group, all ages

Decreasing trends were reported in both male and female subgroups. In terms of the male population, eight countries reported declining trends, and no increasing trends were observed (Fig. 5; Supplementary Fig. 2). Countries with the most significant decreasing trends were the Philippines (AAPC: -17.04, 95% CI: -28.20 to -4.15, *p* = 0.018), Thailand (AAPC: -14.41, 95% CI: -22.50 to -5.48, *p* = 0.007), and Canada (AAPC: -13.42, 95% CI: -22.77 to -2.94, *p* = 0.020). The other 33 countries showed no significant increase or decrease.

Among the female subgroup, a decreasing trend was observed in five countries, while three countries showed increasing trends for tracheal cancer (Supplementary Table 3). Countries with the most significant decreasing trend were France (AAPC: -18.98, 95% CI: -26.19 to -11.06, *p* = 0.001), New Zealand (AAPC: -18.70, 95% CI: -27.61 to -8.69, *p* < 0.001), and Iceland (AAPC: -15.77, 95% CI: -15.84 to -15.71, *p* < 0.001). The three countries with increasing trend were Colombia (AAPC: 19.28, 95% CI: 16.48 to 22.15, *p* < 0.001), Norway (AAPC: 15.57, 95% CI: 0.59 to 32.77, *p* = 0.043), and Kuwait (AAPC: 15.17, 95% CI: 0.34 to 32.20, *p* = 0.045). The remaining 33 countries showed no significant increasing or decreasing trend.

**Fig. 6:**
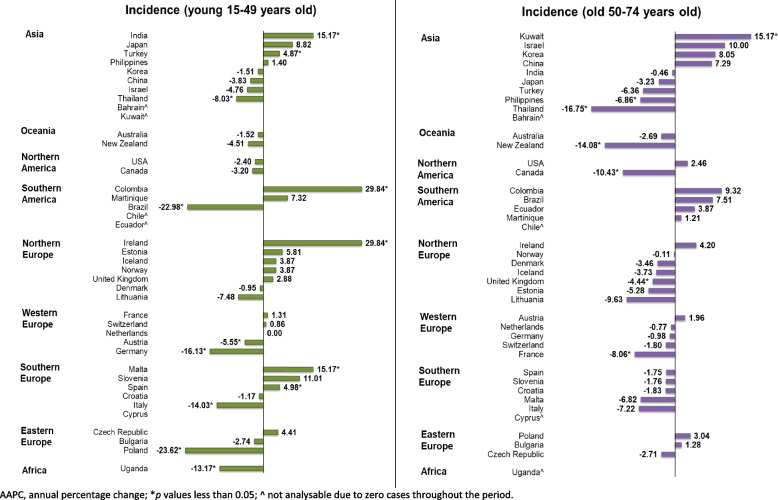
The AAPC of the incidence of tracheal cancer by age group, both sexes

As for the age subgroups, the trend of the younger population was mixed, while the older population was experiencing a decreasing trend of tracheal cancer. There were seven decreasing trends and six increasing trends among the younger population (Supplementary Table 3, Fig. 6). The countries with the most evident decreasing trends were Poland (AAPC: -23.62, 95% CI: -25.67 to -21.52, *p* < 0.001), Brazil (AAPC: -22.98, 95% CI: -25.41 to -20.47, *p* < 0.001), and Germany (AAPC: -16.13, 95% CI: -19.68 to -12.42, *p* < 0.001). The more evident increasing trend was located in Ireland (AAPC: 29.84, 95% CI: 25.74 to 34.06, *p* < 0.001), Columbia (AAPC: 29.84, 95% CI: 25.74 to 34.06, *p* < 0.001), India (AAPC: 15.17, 95% CI: 0.34 to 32.20, *p* = 0.045), and Malta (AAPC: 15.17, 95% CI: 0.34 to 32.20, *p* = 0.045). The rest 28 countries reported no significant trend increase or decrease.

As for the older population, the decreasing trend was more evident than the younger population; six countries observed decreasing trends, and only one showed an increasing trend. The top three countries with most evident decreasing trends were Thailand (AAPC: -16.75, 95% CI: -18.54 to -14.93, *p* < 0.001), New Zealand (AAPC: -14.08, 95% CI: -21.42 to -6.06, *p* = 0.001), and Canada (AAPC: -10.43, 95% CI: -16.00 to -4.48, *p* = 0.004). The only increasing trend was observed in Kuwait (AAPC: 15.17, 95% CI: 0.34 to 32.20, *p* = 0.045). The other 34 countries reported no significant trend increase or decrease (Supplementary Table 3).

## Discussion

### Summary of principal findings

This study comprehensively analysed the disease burden, risk factors, and temporal trends of tracheal cancer. There were three principal findings: (1) Higher incidence of tracheal cancer was found in males, older adults, and European regions and Mediterranean regions, such as Central and Eastern Europe, Southern Europe, and Northern Africa. (2) A higher incidence of tracheal cancer was associated with higher HDI, GDP per capita, higher prevalence of smoking, alcoholism, unhealthy diet, physical inactivity, obesity, hypertension, diabetes, and lipid disorder. (3) There has been an overall decreasing trend in the incidence of tracheal cancer, particularly among males and older adults, while the decline is less pronounced in females and younger individuals.

### Disease burden

The highest burden of tracheal cancer is concentrated in European regions, particularly Central and Eastern Europe and Southern Europe. This trend may be attributed to a higher prevalence of related risk factors, such as alcohol consumption, obesity, metabolic conditions, and environmental factors like air pollution in these areas [16, 17]. Additionally, in Northern Africa, one sub-region has shown a higher incidence of tracheal cancer, potentially due to unregulated tobacco use [18]. Regarding sex differences, the incidence in males is approximately double that of females, likely due to greater exposure to risk factors among men [17]. Another contributing factor may be the higher number of men working in the petroleum industry, where exposure to hydrocarbons—known pollutants—can increase the risk of tracheal cancer [19, 20]. Furthermore, a significant disparity exists, with the incidence of tracheal cancer being over ten times higher in older age groups compared to younger ones. A Norwegian study reported a mean age of 63.8 years (SD = 13.7) [21] for tracheal cancer patients, while a Canadian study [22] indicated a mean age of 68 years, reinforcing the notion that age is a major risk factor for this disease.

### Associated risk factors

In our analysis, the risk of tracheal cancer was significantly associated with higher Human HDI and GDP per capita, correlating with previous studies [17, 23]. Individuals living in countries with elevated HDI and GDP per capita tend to have better health literacy, enabling them to monitor their personal health more effectively. They also have access to advanced screening methods that can help identify potential cancer symptoms. This may explain the higher incidence of tracheal cancer observed in developed countries, such as Slovenia [24].

Smoking is the leading risk factor for tracheal cancer, as supported by various publications [16, 17, 25]. Smoking is the leading risk factor for tracheal cancer, as supported by various publications [8] An American study found that cigarette smoking significantly increases the likelihood of being diagnosed with tracheal cancer and other related malignancies, with smokers having a tenfold higher probability [26]. This may be attributed to the DNA damage caused by the harmful effects of smoking. Additionally, our analysis identified alcohol consumption as another major risk factor for tracheal cancer, often discussed in conjunction with smoking. A Korean study suggested that individuals who both smoke and consume alcohol have a significantly higher risk of developing tracheal cancer, with hazard ratios of 2.3 for males and 5.1 for females, indicating that increased alcohol intake correlates with a higher risk [27, 28]. Beyond smoking and alcohol, other behavioral risk factors contribute to a higher incidence of tracheal cancer. In the Chinese population, unhealthy dietary habits, such as low fruit intake and high meat consumption, may elevate the risk, particularly as urbanization makes it challenging to maintain healthier eating habits [25]. Furthermore, physical inactivity is associated with an increased risk of tracheal cancer, aligning with findings from Cannioto and colleagues, which reported a significant positive association between physical inactivity and tracheal cancer (OR = 2.23, 95% CI: 1.77–2.81) [29]. Hypertension has also been positively associated with tracheal cancer, as indicated by a prospective cohort study that found higher blood pressure levels correlate with increased risk [30]. Our study, along with previous research, also found a positive association between lipid disorders and tracheal cancer [31, 32]. However, while obesity and diabetes were identified as risk factors in our analysis, it is important to note that there is limited supporting evidence in the existing literature.

### Temporal trends

There has been a general decline in the incidence of tracheal cancer, particularly among the male population. This decrease may be attributed to increased regulation of cigarette smoking, including the anti-smoking movement that began in the 1960s, as well as legislation aimed at reducing occupational carcinogens, such as asbestos [17, 17, 33, 17]. However, the decline has been less pronounced among females, likely due to significant indoor air pollution from cooking and a rising prevalence of metabolic risk factors among women [34, 35]. Further research is needed to investigate the reasons behind this phenomenon.

The declining trend of tracheal cancer among the older population may be attributed to improved early detection through increased lung cancer screening using low-dose CT scans [16, 36]. However, the declining trend is less evident among the younger population, likely because lung cancer screening does not include this age group. Additionally, there is a rising trend of obesity and metabolic risk factors among younger individuals [37, 38].

### Strengths and limitations

The current data represents the most comprehensive and up-to-date information available for investigating the distribution, risk factors, and trends of tracheal cancer. However, several limitations should be noted. Due to varying data management strategies, there may be an overestimation of tracheal cancer incidence, particularly in developed countries. Conversely, underestimation may occur in developing countries, where limited resources and inadequate reporting mechanisms can hinder accurate monitoring of each new case. Nonetheless, comparisons among countries by different age and sex groups are less likely to be affected. Additionally, as this study is based on country-level data, ecological bias may have existed. Further research is needed to validate these findings at the individual level.

## Conclusions

This study offers a comprehensive analysis of the disease burden, risk factors, and population-level trends of tracheal cancer. There has been an overall decreasing trend in the incidence of tracheal cancer, particularly among males and older adults, while the decline is less pronounced in females and younger individuals. Further research is recommended to explore the underlying reasons for these epidemiological changes.

## Supplementary Information

Additional file 1: Supplementary Table 1a. Global incidence of tracheal cancer by sex. Supplementary Table 1b. Global incidence of tracheal cancer by age. Supplementary Table 2. Associations of risk factors with tracheal cancer incidence. Supplementary Table 3. Trend analysis of tracheal cancer incidence by country. Supplementary Fig. 1. Trend analysis of tracheal cancer by country. Supplementary Fig. 2. The graphs of the joinpoint regression output.

## Data Availability

Datasets used for this study were publicly available from the Global Cancer Observatory and the Cancer Incidence in Five Continents Plus.
