# Supplementary material for: Incidence, risk factors, and epidemiological trends of tracheal cancer: a global analysis
Source: Mol Cancer. 2024 Dec 18;23:271. doi: 10.1186/s12943-024-02188-4 (PMC11654256; doi:10.1186/s12943-024-02188-4)
Supplement: Supplementary file 1 — Additional file 1: Supplementary Table 1a. Global incidence of tracheal cancer by sex. Supplementary Table 1b. Global incidence of tracheal cancer by age. Supplementary Table 2. Associations of risk factors with tracheal cancer incidence. Supplementary Table 3. Trend analysis of tracheal cancer incidence by country. Supplementary Fig. 1. Trend analysis of tracheal cancer by country. Supplementary Fig. 2. The graphs of the joinpoint regression output. [file 12943_2024_2188_MOESM1_ESM.pdf]

Supplementary Legends

**Supplementary Table 1a.**	Global incidence of tracheal cancer by sex
**Supplementary Table 1b.**	Global incidence of tracheal cancer by age
**Supplementary Table 2.**	Associations of risk factors with tracheal cancer incidence
**Supplementary Table 3.**	Trend analysis of tracheal cancer incidence by country
**Supplementary Figure 1.**	Trend analysis of tracheal cancer by country
**Supplementary Figure 2.**	The graphs of the joinpoint regression output

Table 1a. Global incidence of tracheal cancer by sex

Region	Both sexes		Males		Females	
	New cases	ASR*	New cases	ASR*	New cases	ASR*
**World**	3,474	2.9	2,223	3.8	1,251	2.0
**Asia**	1,904	2.8	1,246	3.8	658	1.9
**Eastern Asia**	1,300	4.0	811	6.0	489	3.0
China	1,171	4.7	726	5.9	445	3.5
Japan	84	2.4	60	3.3	24	1.4
Korea, Democratic People Republic of	14	3.9	7	4.8	7	3.2
Korea, Republic of	31	2.5	18	3.0	13	2.2
Mongolia	0	1.9	0	3.5	0	0.68
**South-Eastern Asia**	179	2.0	155	4.0	24	1.00
Brunei Darussalam	0	4.5	0	9.7	0	1.2
Cambodia	2	1.6	2	3.1	0	0.51
Indonesia	47	1.3	41	2.7	6	0.33
Lao People's Democratic Republic	1	1.7	1	3.5	0	0.46
Malaysia	7	1.5	6	3.0	1	0.36
Myanmar	9	1.6	7	2.8	2	0.62
Philippines	58	3.9	54	9.4	4	0.66
Singapore	1	0.96	0	0.87	1	0.94
Thailand	26	2.0	20	3.5	6	0.75
Timor-Leste	0	1.0	0	1.9	0	0.30
Viet Nam	28	2.0	24	4.1	4	0.56
**South Central Asia**	374	2.0	241	2.0	133	1.00
Afghanistan	4	2.0	3	2.7	1	1.3
Bangladesh	37	2.4	26	3.3	11	1.4
Bhutan	0	1.9	0	1.6	0	2.6
India	236	1.6	153	2.1	83	1.1
Iran, Islamic Republic of	32	4.1	12	2.0	20	5.5
Kazakhstan	12	5.4	10	10.2	2	1.6
Kyrgyzstan	3	3.9	2	6.4	1	2.0
Maldives	0	3.4	0	4.4	0	2.1
Nepal	7	2.5	4	3.2	3	2.2
Pakistan	27	1.6	20	2.3	7	0.80
Sri Lanka	6	1.8	4	2.8	2	1.1
Tajikistan	1	1.5	1	1.7	0	1.3
Turkmenistan	1	2.6	1	4.0	0	1.6

Uzbekistan	8	2.3	5	3.3	3	1.4
**Western Asia**	51	2.0	39	3.0	12	1.00
Armenia	1	2.7	1	4.8	0	1.2
Azerbaijan	2	1.8	2	3.1	0	0.67
Bahrain	0	0.93	0	1.3	0	0.69
Gaza Strip and West Bank	0	1.9	0	3.0	0	0.85
Georgia	1	2.0	1	3.9	0	0.57
Iraq	3	1.2	2	2.0	1	0.47
Israel	4	3.2	2	3.1	2	3.4
Jordan	1	1.3	1	2.0	0	0.66
Kuwait	1	1.8	1	2.6	0	0.66
Lebanon	2	1.9	1	2.2	1	1.8
Oman	0	0.49	0	0.58	0	0.37
Qatar	0	0.54	0	0.51	0	0.60
Saudi Arabia	1	0.40	1	0.47	0	0.32
Syrian Arab Republic	3	1.7	2	2.4	1	1.1
Türkiye	32	2.9	25	5.3	7	0.96
United Arab Emirates	0	0.58	0	0.57	0	0.65
Yemen	0	0.38	0	0.42	0	0.41
**Oceania**	16	2.1	9	2.8	7	1.4
Australia	14	2.8	7	3.0	7	2.6
Fiji	0	0.75	0	1.0	0	0.52
French Polynesia	0	4.4	0	6.4	0	2.4
Guam	0	3.5	0	5.1	0	2.1
New Caledonia	0	4.6	0	6.7	0	2.8
New Zealand	2	2.5	2	4.7	0	1.1
Papua New Guinea	0	1.2	0	1.5	0	0.85
Samoa	0	3.7	0	5.6	0	2.0
Solomon Islands	0	0.68	0	1.1	0	0.34
Vanuatu	0	0.85	0	1.3	0	0.37
**Northern America**	242	3.2	141	3.8	101	2.6
Canada	38	4.1	24	5.5	14	3.1
United States of America	204	3.0	117	3.6	87	2.5
**Latin America and the Caribbean**	264	3.0	170	4.0	94	1.9
**Caribbean**	24	3.0	19	4.0	5	2.0
Bahamas	0	1.8	0	3.2	0	0.74
Barbados	0	1.9	0	3.3	0	0.84

Cuba	16	6.1	12	9.5	4	3.1
Dominican Republic	3	2.6	2	3.7	1	1.5
France, Guadeloupe	1	10.9	1	17.3	0	5.8
France, Martinique	0	2.1	0	3.1	0	3.3
Haiti	1	1.2	1	1.4	0	0.92
Jamaica	1	2.9	1	4.7	0	1.3
Puerto Rico	1	1.7	1	3.7	0	0.40
Saint Lucia	0	2.0	0	4.1	0	0.48
Trinidad and Tobago	1	2.4	1	4.7	0	0.71
**Latin America**	240	3.0	151	4.0	89	2.0
Argentina	48	7.5	31	11.5	17	4.3
Belize	0	2.2	0	3.5	0	0.94
Bolivia	3	2.1	2	3.0	1	1.4
Brazil	113	3.8	71	5.2	42	2.7
Chile	10	1.4	6	2.9	4	2.2
Colombia	10	1.6	7	2.6	3	0.82
Costa Rica	2	1.9	1	1.5	1	1.8
Ecuador	4	1.6	1	0.72	3	2.4
El Salvador	0	1.2	0	1.3	0	1.0
French Guyana	0	3.8	0	6.1	0	1.8
Guatemala	2	0.94	1	1.2	1	0.76
Guyana	0	1.1	0	1.7	0	0.57
Honduras	1	1.7	1	2.3	0	1.2
Mexico	21	1.4	13	1.9	8	0.91
Nicaragua	0	1.4	0	1.8	0	0.99
Panama	1	1.6	1	2.2	0	1.0
Paraguay	2	3.2	2	5.5	0	1.1
Peru	5	1.00	3	1.3	2	0.75
Suriname	0	4.1	0	6.2	0	2.5
Uruguay	5	7.8	3	11.7	2	4.9
Venezuela	13	4.0	8	5.2	5	2.9
**Europe**	880	5.8	590	8.5	290	3.4
**Northern Europe**	61	2.0	38	3.0	23	2.0
Denmark	4	2.8	2	3.5	2	2.2
Estonia	1	2.9	1	5.1	0	1.3
Finland	4	2.8	4	5.4	0	0.47
Iceland	0	3.7	0	2.6	0	6.6
Ireland	2	2.3	1	2.4	1	2.2

Latvia	2	4.6	1	7.5	1	3.1
Lithuania	4	5.6	3	10.5	1	2.3
Norway	4	3.6	2	3.5	2	3.7
Sweden	3	1.3	2	1.7	1	0.96
United Kingdom	37	2.3	22	2.9	15	1.8
**Western Europe**	201	5.0	126	6.0	75	3.0
Austria	6	3.5	4	4.6	2	2.5
Belgium	8	3.4	4	3.5	4	3.6
France (metropolitan)	81	5.9	54	8.1	27	4.0
Germany	87	4.3	55	5.7	32	3.1
Luxembourg	0	3.5	0	4.3	0	2.9
Switzerland	6	3.3	3	4.1	3	2.7
The Netherlands	13	3.3	6	2.9	7	3.9
**Southern Europe**	174	5.0	108	6.0	66	3.0
Albania	3	4.7	2	6.8	1	2.3
Bosnia Herzegovina	4	6.6	3	9.8	1	3.6
Croatia	10	10.1	7	16.7	3	4.6
Cyprus	1	5.0	1	6.8	0	3.3
Greece	15	5.8	10	8.2	5	3.6
Italy	83	4.9	49	6.1	34	4.0
Malta	0	3.5	0	4.6	0	2.7
Montenegro	1	6.8	1	9.7	0	3.9
North Macedonia	2	5.7	1	8.2	1	3.3
Portugal	14	4.3	10	7.9	4	3.0
Serbia	13	7.4	8	9.8	5	5.4
Slovenia	3	6.3	2	7.2	1	5.8
Spain	25	2.3	14	2.5	11	2.5
**Eastern Europe**	444	9.0	318	14.0	126	4.0
Belarus	16	8.9	12	17.1	4	3.8
Bulgaria	10	7.1	7	11.1	3	4.1
Czechia	10	3.9	6	4.9	4	3.0
Hungary	26	12.5	14	16.4	12	10.7
Moldova	4	5.9	3	10.9	1	2.2
Poland	61	4.3	30	8.3	31	7.4
Romania	31	8.0	22	12.5	9	4.5
Russian Federation	237	8.6	186	16.9	51	3.4
Slovakia	5	4.3	3	6.2	2	2.8

Ukraine	44	5.5	35	10.7	9	2.1
**Africa**	168	1.6	67	1.5	101	1.6
**Northern Africa**	36	1.00	19	1.00	17	1.00
Algeria	12	1.3	9	2.6	3	1.0
Egypt	12	0.79	4	0.78	8	1.4
Libya	1	1.5	1	1.8	0	1.0
Morocco	7	1.6	3	1.2	4	1.6
Sudan	1	0.23	0	0.17	1	0.62
Tunisia	3	1.8	2	2.1	1	1.4
**Sub-Saharan Africa**	132	2.0	48	2.0	84	2.0
Angola	2	1.1	1	1.1	1	1.1
Benin	0	0.66	0	0.68	0	0.76
Botswana	0	1.6	0	2.1	0	1.1
Burkina Faso	2	1.7	0	0.99	2	3.1
Burundi	1	1.5	0	1.4	1	1.9
Cameroon	2	1.4	1	1.4	1	1.3
Cape Verde	0	0.97	0	0.60	0	1.7
Central African Republic	0	0.90	0	0.88	0	0.99
Chad	0	0.88	0	1.1	0	0.43
Comoros	0	0.79	0	0.86	0	0.73
Congo, Democratic People Republic of	5	0.96	2	0.91	3	1.1
Congo, Republic of	0	0.60	0	0.68	0	0.54
Côte d'Ivoire	2	1.1	1	1.1	1	0.93
Djibouti	0	1.4	0	1.2	0	1.7
Equatorial Guinea	0	1.2	0	0.96	0	1.7
Eritrea	0	1.4	0	1.2	0	1.9
Eswatini	0	1.8	0	2.5	0	1.6
Ethiopia	14	1.6	4	1.3	10	2.4
France, La Réunion	2	9.6	1	12.0	1	6.2
Gabon	0	1.4	0	1.1	0	1.8
Ghana	3	1.1	1	1.0	2	1.3
Guinea	2	1.4	1	1.7	1	1.3
Guinea-Bissau	0	0.93	0	1.0	0	0.92
Kenya	10	0.79	1	1.2	9	1.7
Lesotho	0	1.9	0	1.6	0	2.5
Liberia	0	1.2	0	1.3	0	1.00
Madagascar	2	0.92	1	0.92	1	1.0
Malawi	0	0.68	0	0.73	0	0.69

Mali	2	1.4	1	1.5	1	1.4
Mauritania	0	0.85	0	0.92	0	0.81
Mauritius	2	5.1	1	6.0	1	4.5
Mozambique	1	0.58	0	0.77	1	0.46
Namibia	0	2.5	0	2.7	0	2.8
Niger	0	0.42	0	0.60	0	0.06
Nigeria	10	0.76	3	0.62	7	1.1
Rwanda	1	1.2	1	1.5	0	1.0
Sao Tome and Principe	0	5.6	0	5.3	0	6.5
Senegal	2	1.2	1	1.4	1	1.0
Sierra Leone	0	0.55	0	0.90	0	0.01
Somalia	1	1.5	0	1.3	1	2.1
South Africa	47	8.0	20	9.1	27	7.6
South Sudan	0	0.93	0	0.80	0	1.2
Tanzania, United Republic of	4	1.1	1	0.90	3	1.5
The Republic of the Gambia	0	2.2	0	3.1	0	0.59
Togo	0	0.84	0	1.0	0	0.60
Uganda	11	4.9	4	5.3	7	4.6
Zambia	1	1.3	0	1.5	1	1.5
Zimbabwe	3	4.0	2	6.9	1	2.1

ASR*: per 10,000,000 persons

Supplementary Table 1b. Global incidence of tracheal cancer by age

Region	Young		Old	
	New cases	ASR*	New cases	ASR*
**World**	472	1.2	2045	11.9
**Asia**	305	1.2	1109	10.7
**Eastern Asia**	164	2.0	772	15.0
China	153	2.1	709	16.1
Japan	5	0.85	35	7.9
Korea, Democratic People Republic of	2	1.8	9	13.7
Korea, Republic of	4	1.4	19	9.9
Mongolia	0	0.55	0	7.0
**South-Eastern Asia**	36	1.00	111	8.0
Brunei Darussalam	0	7.2	0	8.2
Cambodia	1	1.4	1	5.5
Indonesia	14	0.93	26	4.8
Lao People's Democratic Republic	0	1.4	1	6.5
Malaysia	2	1.0	3	5.3
Myanmar	2	0.86	6	5.8
Philippines	5	0.98	42	22.7
Singapore	0	0.84	1	3.6
Thailand	5	1.2	13	6.0
Timor-Leste	0	0.61	0	3.7
Viet Nam	7	1.4	18	8.1
**South Central Asia**	89	1.00	201	6.0
Afghanistan	2	0.89	2	6.8
Bangladesh	9	1.0	20	8.1
Bhutan	0	0.60	0	6.3
India	55	0.70	133	5.4
Iran, Islamic Republic of	9	2.1	11	7.3
Kazakhstan	2	1.7	8	20.3
Kyrgyzstan	0	0.94	1	14.0
Maldives	0	1.3	0	10.4
Nepal	2	1.0	3	7.5
Pakistan	7	0.68	13	4.9
Sri Lanka	1	0.82	3	5.8
Tajikistan	0	0.97	1	4.5
Turkmenistan	0	1.1	1	8.7

Uzbekistan	2	0.90	5	8.3
**Western Asia**	16	1.00	25	5.0
Armenia	0	1.2	1	7.7
Azerbaijan	1	1.6	1	4.7
Bahrain	0	0.29	0	1.7
Gaza Strip and West Bank	0	1.0	0	5.3
Georgia	0	1.7	1	6.2
Iraq	1	0.45	1	3.3
Israel	1	1.9	2	8.8
Jordan	1	1.4	0	3.7
Kuwait	0	0.38	1	8.8
Lebanon	1	1.5	1	5.0
Oman	0	0.27	0	1.5
Qatar	0	0.33	0	1.4
Saudi Arabia	1	0.34	1	1.2
Syrian Arab Republic	1	1.3	1	4.4
Türkiye	8	1.9	15	8.5
United Arab Emirates	0	0.11	0	1.6
Yemen	1	1.1	0	0.90
**Oceania**	2	1.5	9	10.0
Australia	2	1.5	8	11.0
Fiji	0	1.0	0	2.6
French Polynesia	0	3.0	0	19.4
Guam	0	1.3	0	13.1
New Caledonia	0	4.5	0	18.5
New Zealand	0	1.5	1	9.3
Papua New Guinea	0	1.1	0	4.6
Samoa	0	2.7	0	14.7
Solomon Islands	0	1.00	0	2.4
Vanuatu	0	0.76	0	3.3
**Northern America**	17	0.94	146	12.4
Canada	2	0.76	20	15.1
United States of America	15	0.97	126	12.0
**Latin America and the Caribbean**	34	0.95	144	10.2
**Caribbean**	0	0.00	23	24.0
Bahamas	0	0.00	0	10.6
Barbados	0	0.00	0	11.9
Cuba	0	0.00	15	38.9

Dominican Republic	0	0.00	3	14.9
France, Guadeloupe	0	0.00	1	68.7
France, Martinique	0	0.00	0	13.5
Haiti	0	0.00	1	6.7
Jamaica	0	0.00	1	17.6
Puerto Rico	0	0.00	1	10.9
Saint Lucia	0	0.00	0	14.1
Trinidad and Tobago	0	0.00	1	14.5
**Latin America**	34	1.00	121	10.0
Argentina	4	1.5	23	20.9
Belize	0	0.39	0	7.6
Bolivia	0	0.58	1	5.7
Brazil	17	1.5	57	11.4
Chile	1	0.59	5	8.3
Colombia	2	0.77	8	7.3
Costa Rica	1	2.9	1	5.3
Ecuador	1	0.68	1	4.0
El Salvador	0	0.80	0	3.0
French Guyana	0	1.8	0	11.9
Guatemala	1	0.62	1	2.5
Guyana	0	0.56	0	3.3
Honduras	0	0.68	1	5.5
Mexico	3	0.47	10	4.0
Nicaragua	0	0.60	0	3.9
Panama	0	0.40	0	4.6
Paraguay	0	0.83	1	10.0
Peru	2	1.1	2	3.8
Suriname	0	2.7	0	11.3
Uruguay	0	1.5	3	32.7
Venezuela	2	1.6	7	12.2
**Europe**	73	1.8	590	23.3
**Northern Europe**	3	1.00	38	10.0
Denmark	0	1.0	3	12.8
Estonia	0	0.69	1	17.5
Finland	0	1.4	3	12.2
Iceland	0	0.55	0	10.0
Ireland	0	0.55	2	12.5
Latvia	0	1.1	2	22.3

Lithuania	0	1.6	2	26.1
Norway	0	1.1	2	12.2
Sweden	0	0.59	2	6.3
United Kingdom	3	1.0	21	9.7
**Western Europe**	13	1.00	129	19.0
Austria	1	1.3	5	15.1
Belgium	1	1.7	5	12.7
France (metropolitan)	5	1.4	59	26.7
Germany	4	1.1	51	17.5
Luxembourg	0	0.75	0	11.9
Switzerland	1	1.8	3	9.0
The Netherlands	1	1.6	6	10.4
**Southern Europe**	10	1.00	106	19.0
Albania	0	2.2	2	17.5
Bosnia Herzegovina	1	4.2	3	27.8
Croatia	0	0.46	7	46.3
Cyprus	0	1.6	1	20.1
Greece	0	0.76	9	25.4
Italy	4	1.1	47	20.8
Malta	0	0.67	0	14.3
Montenegro	0	2.9	1	30.3
North Macedonia	0	2.6	2	25.3
Portugal	1	1.9	9	24.0
Serbia	1	2.5	10	34.4
Slovenia	0	0.98	2	28.8
Spain	3	1.3	13	7.8
**Eastern Europe**	47	3.0	317	33.0
Belarus	2	3.4	10	31.2
Bulgaria	1	3.3	7	30.0
Czechia	1	1.2	5	13.3
Hungary	2	3.1	17	53.2
Moldova	1	3.2	3	24.2
Poland	3	1.6	30	25.3
Romania	5	4.2	20	32.0
Russian Federation	26	3.1	186	40.0
Slovakia	0	0.75	4	20.0
Ukraine	6	2.3	35	24.5
**Africa**	41	0.73	47	3.0

**Northern Africa**	0	0.00	13	3.0
Algeria	0	0.00	2	2.6
Egypt	0	0.00	4	1.9
Libya	0	0.00	0	3.1
Morocco	0	0.00	5	5.4
Sudan	0	0.00	0	0.50
Tunisia	0	0.00	2	4.7
**Sub-Saharan Africa**	41	1.00	34	3.0
Angola	1	0.58	1	2.6
Benin	0	0.56	0	0.82
Botswana	0	0.82	0	3.5
Burkina Faso	1	0.79	1	3.2
Burundi	0	0.96	0	2.8
Cameroon	1	0.70	1	2.6
Cape Verde	0	0.24	0	2.6
Central African Republic	0	0.37	0	1.9
Chad	1	0.85	0	1.5
Comoros	0	0.00	0	2.0
Congo, Democratic People Republic of	2	0.54	1	1.8
Congo, Republic of	0	0.19	0	1.4
Côte d'Ivoire	1	0.92	0	1.9
Djibouti	0	1.2	0	2.4
Equatorial Guinea	0	0.87	0	2.7
Eritrea	0	0.99	0	2.8
Eswatini	0	1.6	0	3.1
Ethiopia	6	1.2	3	3.1
France, La Réunion	0	3.9	1	20.2
Gabon	0	0.86	0	2.9
Ghana	1	0.83	1	2.2
Guinea	1	2.4	0	2.5
Guinea-Bissau	0	0.98	0	1.8
Kenya	5	1.7	2	3.5
Lesotho	0	0.77	0	3.5
Liberia	0	1.7	0	2.0
Madagascar	1	0.63	1	1.8
Malawi	0	0.38	0	1.2
Mali	1	1.0	0	3.0
Mauritania	0	0.75	0	1.7

Mauritius	0	2.0	1	23.2
Mozambique	0	0.29	0	1.3
Namibia	0	1.4	0	5.4
Niger	0	0.22	0	0.91
Nigeria	2	0.25	3	1.5
Rwanda	1	1.1	0	2.5
Sao Tome and Principe	0	2.3	0	13.5
Senegal	1	1.4	0	2.3
Sierra Leone	0	0.24	0	1.3
Somalia	0	0.92	0	3.1
South Africa	10	3.0	15	16.4
South Sudan	0	0.52	0	1.9
Tanzania, United Republic of	2	0.98	1	2.1
The Republic of the Gambia	0	1.3	0	4.1
Togo	0	0.68	0	1.2
Uganda	2	1.6	1	2.3
Zambia	1	1.0	0	2.3
Zimbabwe	0	0.77	1	6.0

ASR*: per 10,000,000 persons

Table 2. Associations of risk factors with tracheal cancer incidence

Outcome	Risk factor	Overall			
		β	*95% CI*		*P*
**All Sexes and ages**	HDI	0.643	0.391	0.894	<0.001*
	GDP per capita	0.226	0.030	0.422	0.024*
	Smoking	0.300	0.242	0.357	<0.001*
	Alcohol drinking	0.212	0.140	0.283	<0.001*
	Dietary	0.052	0.016	0.088	0.005*
	Physical inactivity	0.134	0.005	0.264	0.042*
	Obesity	0.097	0.063	0.132	<0.001*
	Hypertension	0.120	0.075	0.164	<0.001*
	Diabetes	0.149	0.076	0.222	<0.001*
	Lipid	0.123	0.094	0.153	<0.001*
**Male**	HDI	0.923	0.546	1.300	<0.001*
	GDP per capita	0.262	-0.037	0.561	0.086
	Smoking	0.319	0.257	0.381	<0.001*
	Alcohol drinking	0.275	0.202	0.348	<0.001*
	Dietary	0.109	0.070	0.148	<0.001*
	Physical inactivity	0.047	-0.147	0.242	0.632
	Obesity	0.145	0.094	0.196	<0.001*
	Hypertension	0.183	0.123	0.244	<0.001*
	Diabetes	0.202	0.099	0.305	<0.001*
	Lipid	0.176	0.133	0.219	<0.001*
	HDI	0.462	0.271	0.652	<0.001*
	GDP per capita	0.198	0.054	0.343	0.007*
	Smoking	0.176	0.122	0.230	<0.001*
	Alcohol drinking	0.139	0.059	0.220	0.001*

**Female**	Dietary	-0.016	-0.049	0.017	0.344
	Physical inactivity	0.186	0.098	0.274	<0.001*
	Obesity	0.062	0.036	0.088	<0.001*
	Hypertension	0.039	0.005	0.074	0.026*
	Diabetes	0.100	0.043	0.157	0.001*
	Lipid	0.077	0.053	0.101	<0.001*
**Young**	HDI	0.222	0.086	0.359	0.002*
	GDP per capita	0.065	-0.035	0.165	0.203
	Smoking	0.120	0.082	0.159	<0.001*
	Alcohol drinking	0.078	0.041	0.116	<0.001*
	Dietary	0.033	0.014	0.051	0.001*
	Physical inactivity	0.004	-0.067	0.075	0.910
	Obesity	0.024	0.005	0.043	0.015*
	Hypertension	0.048	0.012	0.084	0.008*
	Diabetes	0.029	-0.063	0.121	0.532
	Lipid	0.054	0.035	0.073	<0.001*
**Old**	HDI	2.629	1.470	3.788	<0.001*
	GDP per capita	0.830	-0.095	1.755	0.078
	Smoking	1.175	0.950	1.399	<0.001*
	Alcohol drinking	0.716	0.362	1.069	<0.001*
	Dietary	0.106	-0.041	0.253	0.155
	Physical inactivity	0.554	-0.007	1.115	0.053
	Obesity	0.345	0.220	0.470	<0.001*
	Hypertension	0.185	0.014	0.356	0.034*
	Diabetes	0.115	-0.074	0.304	0.230
	Lipid	0.534	0.397	0.671	<0.001*

The analysis was conducted using univariable linear regression model at a country level.
β, beta coefficient. The beta coefficient can be interpreted as the change in incidence or mortality associated with one percent increase of a certain risk factor.
CI, confidence interval; ASR, age-standardized rate; HDI, human development index;
GDP, gross domestic products.
* p values less than 0.05.

Supplementary Table 3· Trend analysis of tracheal cancer incidence

a) Male

Region	AAPC	Lower CI	Upper CI	p-value	Significant
Asia					
Bahrain	NA	NA	NA	NA	NA
China	-9.77	-22.88	5.58	0.170	
India	4.20	-3.29	12.28	0.239	
Israel	1.21	-7.94	11.27	0.777	
Japan	1.32	-22.30	32.12	0.912	
Korea	0.51	-10.19	12.49	0.919	
Kuwait	NA	NA	NA	NA	NA
Philippines	-17.04	-28.2	-4.15	0.018	*
Thailand	-14.41	-22.5	-5.48	0.007	*
Turkey	-5.23	-18.39	10.05	0.431	
Oceania					
Australia	-2.13	-10.20	6.66	0.580	
New Zealand	-2.08	-15.46	13.41	0.779	
Northern America					
Canada	-13.42	-22.77	-2.94	0.020	*
USA	0.01	-10.5	11.76	0.998	
Southern America					
Brazil	3.28	-5.49	12.86	0.426	
Chile	NA	NA	NA	NA	NA
Colombia	-5.27	-13.93	4.26	0.269	
Ecuador	8.81	-1.32	19.99	0.090	
Martinique	1.21	-6.85	9.98	0.776	
Northern Europe					
Denmark	-8.67	-20.01	4.27	0.153	

Estonia	-7.21	-13.44	-0.54	0.038	*
Iceland	2.64	-6.68	12.89	0.591	
Ireland	6.70	-3.18	17.58	0.162	
Lithuania	-10.02	-17.20	-2.23	0.019	*
Norway	-12.51	-22.70	-0.98	0.038	*
United Kingdom	-5.68	-10.15	-0.98	0.024	*
Western Europe					
Austria	-0.24	-10.21	10.84	0.96	
France	-3.70	-10.00	3.04	0.235	
Germany	-9.82	-25.99	9.89	0.262	
Netherlands	0.92	-5.18	7.42	0.743	
Switzerland	3.72	-7.00	15.67	0.462	
Southern Europe					
Croatia	-0.85	-11.69	11.31	0.869	
Cyprus	3.87	-5.80	14.55	0.446	
Italy	-7.07	-26.26	17.10	0.534	
Malta	12.13	-4.75	32.01	0.169	
Slovenia	1.13	-14.38	19.45	0.880	
Spain	1.41	-7.38	11.03	0.732	
Eastern Europe					
Bulgaria	-1.12	-7.86	6.11	0.723	
Czech Republic	-6.68	-15.88	3.52	0.191	
Poland	-11.39	-22.17	0.87	0.064	
Africa					
Uganda	-13.17	-24.36	-0.34	0.045	*

AAPC, annual percentage change; CI, confidence interval; * p values less than 0·05.

NA, not available as it reported zero cases during the period and joinpoint regression could not be performed in such circumstances.

b) Female

Region	AAPC	Lower CI	Upper CI	p-value	Significant
Asia					
Bahrain	NA	NA	NA	NA	NA
China	0.37	-13.08	15.89	0.954	
India	-1.20	-9.08	7.36	0.776	
Israel	6.48	-11.38	27.95	0.453	
Japan	2.28	-14.51	22.37	0.779	
Korea	5.56	-4.98	17.27	0.270	
Kuwait	15.17	0.34	32.20	0.045	*
Philippines	-0.21	-6.77	6.81	0.951	
Thailand	-1.86	-6.43	2.94	0.391	
Turkey	-7.16	-20.71	8.71	0.356	
Oceania					
Australia	2.82	-13.72	22.53	0.756	
New Zealand	-18.70	-27.61	-8.69	<0.001	*
Northern America					
Canada	-6.13	-19.20	9.05	0.359	
USA	0.65	-11.44	14.38	0.921	
Southern America					
Brazil	-7.82	-8.39	-7.25	<0.001	*
Chile	NA	NA	NA	NA	NA
Colombia	19.28	16.48	22.15	<0.001	*
Ecuador	NA	NA	NA	NA	NA
Martinique	7.32	-6.51	23.18	0.316	
Northern Europe					
Denmark	-2.19	-20.13	19.78	0.808	
Estonia	0.63	-12.33	15.51	0.929	

Iceland	-15.77	-15.84	-15.71	<0.001	*
Ireland	-10.64	-23.86	4.87	0.168	
Lithuania	-5.26	-14.99	5.59	0.284	
Norway	15.57	0.59	32.77	0.043	*
United Kingdom	-1.23	-8.86	7.04	0.732	
Western Europe					
Austria	-0.15	-10.68	11.61	0.978	
France	-18.98	-26.19	-11.06	0.001	*
Germany	7.02	-9.88	27.08	0.389	
Netherlands	-5.04	-12.50	3.06	0.183	
Switzerland	3.10	-3.21	9.82	0.298	
Southern Europe					
Croatia	-3.37	-18.23	14.19	0.648	
Cyprus	NA	NA	NA	NA	NA
Italy	-18.55	-35.17	2.32	0.072	
Malta	NA	NA	NA	NA	NA
Slovenia	-2.14	-11.05	7.67	0.616	
Spain	-6.46	-10.62	-2.11	0.004	*
Eastern Europe					
Bulgaria	2.72	-9.82	16.99	0.648	
Czech Republic	4.26	-5.43	14.94	0.353	
Poland	11.59	-5.42	31.67	0.194	
Africa					
Uganda	NA	NA	NA	NA	NA

AAPC, annual percentage change; CI, confidence interval; * p values less than 0·05.

NA, not available as it reported zero cases during the period and joinpoint regression could not be performed in such circumstances.

c) Both

Region	AAPC	Lower CI	Upper CI	p-value	Significant
Asia					
Bahrain	NA	NA	NA	NA	NA
China	-3.95	-19.90	15.17	0.623	
India	3.41	-4.09	11.50	0.334	
Israel	1.66	-15.05	21.66	0.838	
Japan	-3.76	-22.08	18.87	0.687	
Korea	2.96	-4.99	11.58	0.427	
Kuwait	15.17	0.34	32.20	0.045	*
Philippines	-9.84	-24.18	7.22	0.205	
Thailand	-15.06	-21.76	-7.78	0.002	*
Turkey	-5.35	-19.30	11.02	0.450	
Oceania					
Australia	-2.41	-7.88	3.37	0.356	
New Zealand	-14.40	-21.92	-6.15	0.005	*
Northern America					
Canada	-9.17	-15.93	-1.86	0.021	*
USA	0.37	-8.32	9.88	0.927	
Southern America					
Brazil	5.96	-3.86	16.78	0.207	
Chile	NA	NA	NA	NA	NA
Colombia	4.57	-3.94	13.83	0.259	
Ecuador	10.26	-0.01	21.58	0.050	
Martinique	6.10	-3.78	17.00	0.235	
Northern Europe					
Denmark	-14.60	-35.27	12.67	0.264	
Estonia	-2.06	-12.12	9.16	0.670	

Iceland	0.15	-9.61	10.96	0.978	
Ireland	3.69	-12.73	23.19	0.641	
Lithuania	-9.60	-24.80	8.67	0.242	
Norway	-2.32	-18.59	17.20	0.774	
United Kingdom	-3.49	-6.55	-0.32	0.035	*
Western Europe					
Austria	0.66	-7.10	9.06	0.855	
France	-6.75	-11.02	-2.28	0.009	*
Germany	-4.24	-17.01	10.49	0.505	
Netherlands	-1.64	-7.13	4.17	0.524	
Switzerland	-0.28	-11.44	12.28	0.957	
Southern Europe					
Croatia	-0.69	-10.01	9.59	0.875	
Cyprus	3.87	-5.80	14.55	0.446	
Italy	-12.21	-24.67	2.31	0.085	
Malta	13.52	-3.95	34.17	0.137	
Slovenia	-1.48	-14.47	13.49	0.814	
Spain	0.14	-12.21	14.23	0.984	
Eastern Europe					
Bulgaria	-0.51	-6.35	5.69	0.849	
Czech Republic	-2.36	-11.64	7.89	0.639	
Poland	-10.87	-26.55	8.17	0.208	
Africa					
Uganda	-13.17	-24.36	-0.34	0.045	*

AAPC, annual percentage change; CI, confidence interval; * p values less than 0·05.

NA, not available as it reported zero cases during the period and joinpoint regression could not be performed in such circumstances.

d) Young

Region	AAPC	Lower CI	Upper CI	p-value	Significant
Asia					
Bahrain	NA	NA	NA	NA	NA
China	-3.83	-21.42	17.71	0.705	
India	15.17	0.34	32.20	0.045	*
Israel	-4.76	-18.40	11.15	0.536	
Japan	8.82	-0.13	18.56	0.053	
Korea	-1.51	-10.82	8.78	0.733	
Kuwait	NA	NA	NA	NA	NA
Philippines	1.40	-3.29	6.32	0.565	
Thailand	-8.03	-12.07	-3.80	0.003	*
Turkey	4.87	3.30	6.47	<0.001	*
Oceania					
Australia	-1.52	-13.45	12.05	0.816	
New Zealand	-4.51	-20.92	15.29	0.631	
Northern America					
Canada	-3.20	-18.61	15.14	0.677	
USA	-2.40	-12.98	9.47	0.638	
Southern America					
Brazil	-22.98	-25.41	-20.47	<0.001	*
Chile	NA	NA	NA	NA	NA
Colombia	29.84	25.74	34.06	<0.001	*
Ecuador	NA	NA	NA	NA	NA
Martinique	7.32	-6.51	23.18	0.316	
Northern Europe					
Denmark	-0.95	-7.68	6.27	0.763	
Estonia	5.81	-6.75	20.06	0.381	

Iceland	3.87	-5.80	14.55	0.446	
Ireland	29.84	25.74	34.06	<0.001	*
Lithuania	-7.48	-20.10	7.13	0.299	
Norway	3.87	-5.80	14.55	0.446	
United Kingdom	2.88	-11.70	19.87	0.679	
Western Europe					
Austria	-5.55	-9.26	-1.69	0.011	*
France	1.31	-10.99	15.30	0.823	
Germany	-16.13	-19.68	-12.42	<0.001	*
Netherlands	0.00	-13.02	14.97	1.000	
Switzerland	0.86	-3.53	5.44	0.671	
Southern Europe					
Croatia	-1.17	-9.40	7.81	0.763	
Cyprus	NA	NA	NA	NA	NA
Italy	-14.03	-22.46	-4.69	0.010	*
Malta	15.17	0.34	32.20	0.045	*
Slovenia	11.01	-7.50	33.22	0.262	
Spain	4.98	4.09	5.88	<0.001	*
Eastern Europe					
Bulgaria	-2.74	-14.87	11.11	0.643	
Czech Republic	4.41	-22.35	40.40	0.775	
Poland	-23.62	-25.67	-21.52	<0.001	*
Africa					
Uganda	-13.17	-24.36	-0.34	0.045	*

AAPC, annual percentage change; CI, confidence interval; * p values less than 0·05.

NA, not available as it reported zero cases during the period and joinpoint regression could not be performed in such circumstances.

e) Old

Region	AAPC	Lower CI	Upper CI	p-value	Significant
Asia					
Bahrain	NA	NA	NA	NA	NA
China	7.29	-5.51	21.83	0.237	
India	-0.46	-7.09	6.65	0.882	
Israel	10.00	-0.85	22.04	0.067	
Japan	-3.23	-19.63	16.50	0.694	
Korea	8.05	-3.62	21.13	0.157	
Kuwait	15.17	0.34	32.20	0.045	*
Philippines	-6.86	-12.40	-0.97	0.023	*
Thailand	-16.75	-18.54	-14.93	<0.001	*
Turkey	-6.36	-19.45	8.85	0.344	
Oceania					
Australia	-2.69	-10.28	5.53	0.460	
New Zealand	-14.08	-21.42	-6.06	0.001	*
Northern America					
Canada	-10.43	-16.00	-4.48	0.004	*
USA	2.46	-10.07	16.73	0.679	
Southern America					
Brazil	7.51	-2.97	19.12	0.142	
Chile	NA	NA	NA	NA	NA
Colombia	9.32	-4.29	24.85	0.189	
Ecuador	3.87	-5.80	14.55	0.446	
Martinique	1.21	-6.85	9.98	0.776	
Northern Europe					
Denmark	-3.46	-18.40	14.22	0.642	
Estonia	-5.28	-13.36	3.55	0.198	

Iceland	-3.73	-12.70	6.16	0.446	
Ireland	4.20	-7.30	17.13	0.440	
Lithuania	-9.63	-20.23	2.37	0.098	
Norway	-0.11	-17.35	20.71	0.989	
United Kingdom	-4.44	-7.72	-1.04	0.017	*
Western Europe					
Austria	1.96	-11.47	17.42	0.760	
France	-8.06	-15.20	-0.32	0.043	*
Germany	-0.98	-16.29	17.12	0.895	
Netherlands	-0.77	-9.58	8.91	0.853	
Switzerland	-1.80	-10.88	8.19	0.676	
Southern Europe					
Croatia	-1.83	-12.77	10.48	0.728	
Cyprus	NA	NA	NA	NA	NA
Italy	-7.22	-22.50	11.07	0.365	
Malta	-6.82	-18.82	6.96	0.316	
Slovenia	-1.76	-14.43	12.79	0.775	
Spain	-1.75	-16.79	16.01	0.835	
Eastern Europe					
Bulgaria	1.28	-3.03	5.78	0.520	
Czech Republic	-2.71	-14.81	11.10	0.646	
Poland	3.04	-9.88	17.80	0.620	
Africa					
Uganda	NA	NA	NA	NA	NA

AAPC, annual percentage change; CI, confidence interval; * p values less than 0·05.

NA, not available as it reported zero cases during the period and joinpoint regression could not be performed in such circumstances.

Supplementary Figure 1: Trend analysis of tracheal cancer

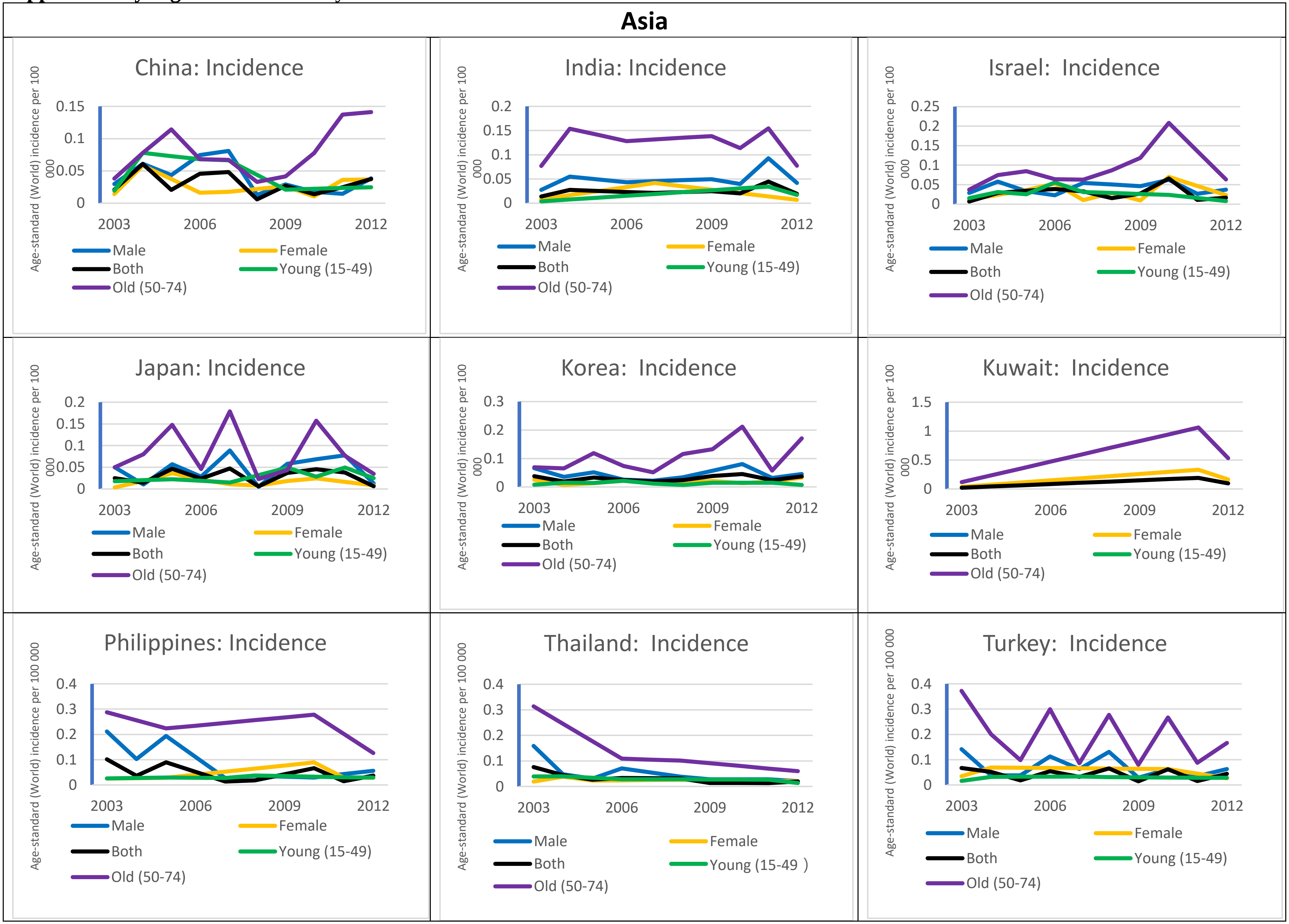

Oceania

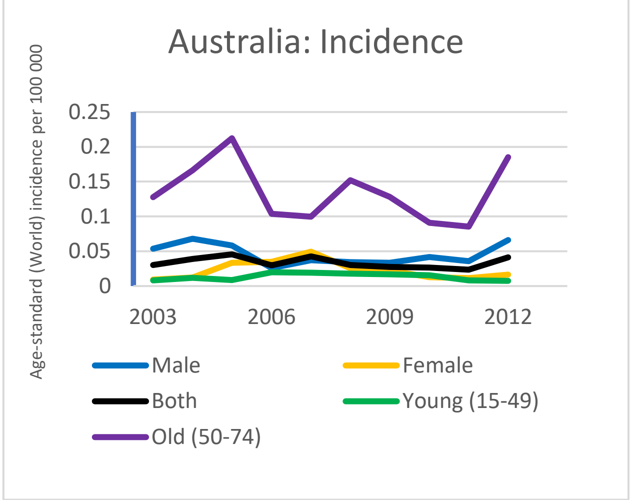

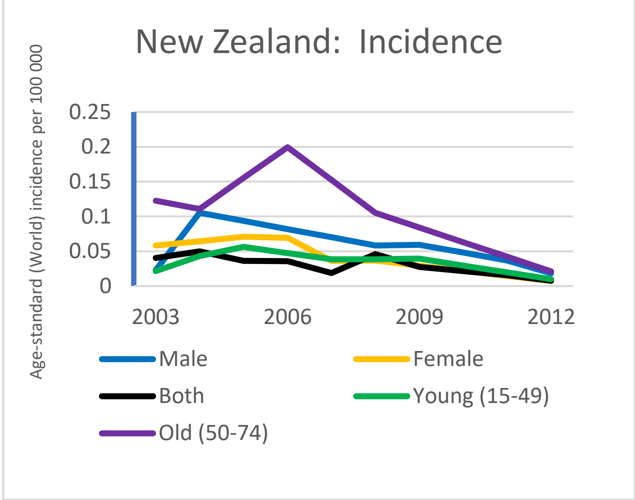

Northern America

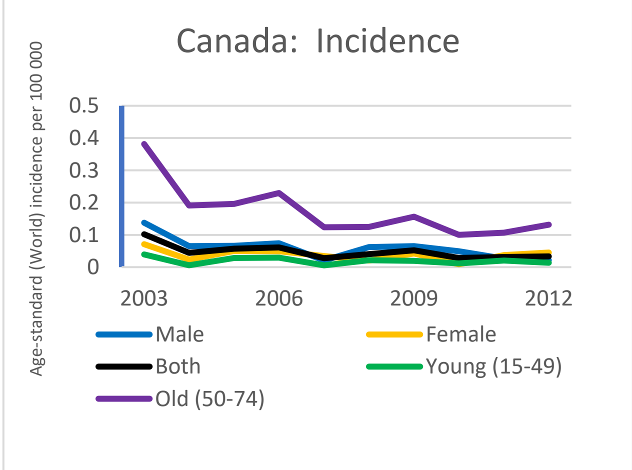

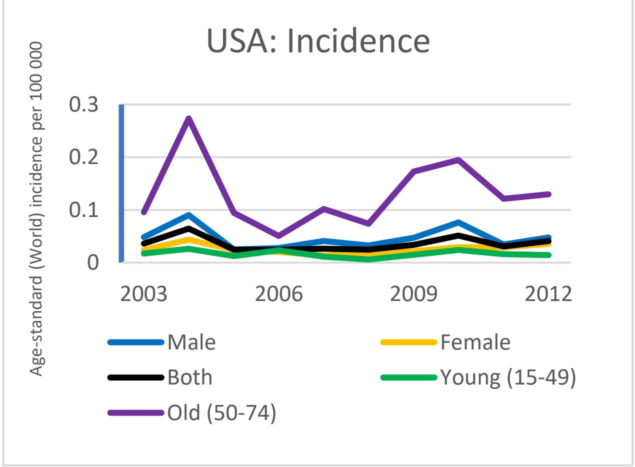

Southern America

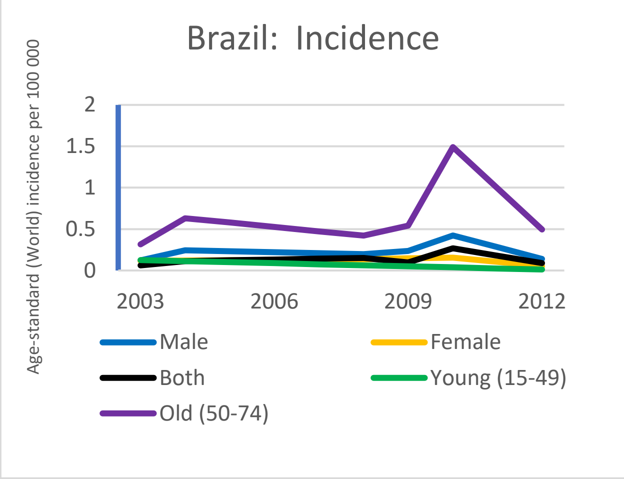

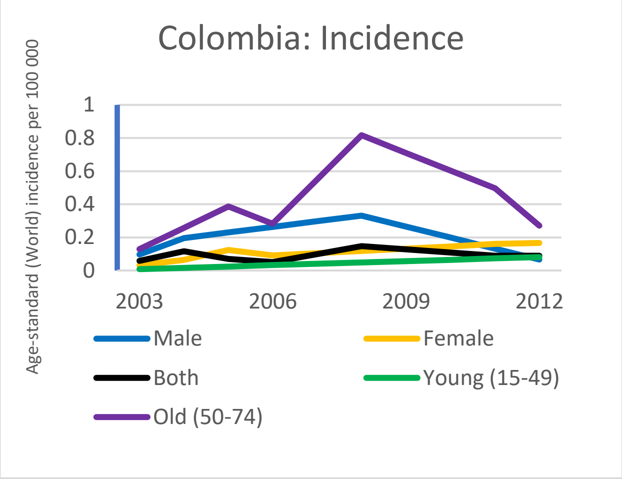

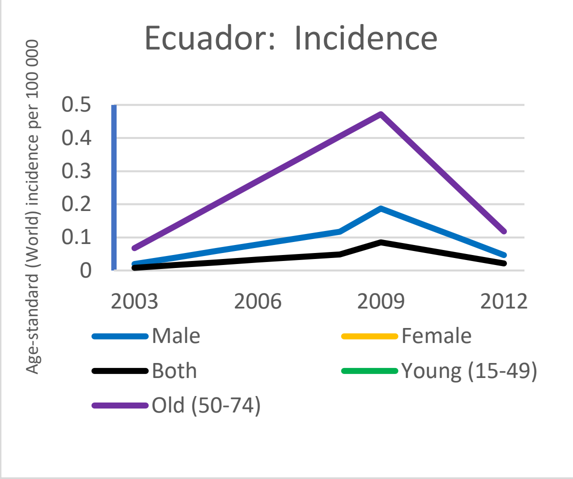

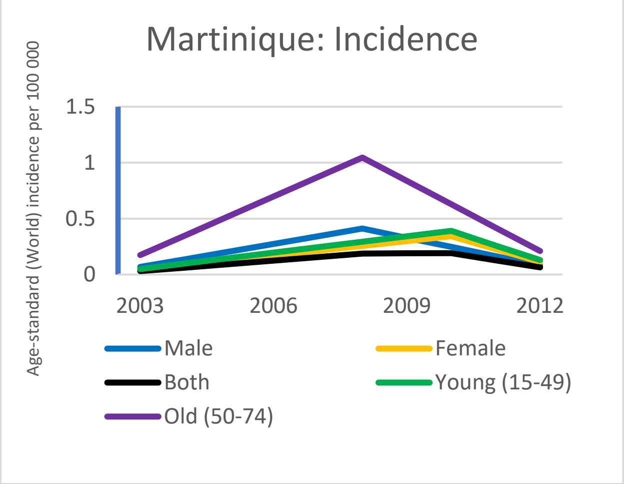

Northern Europe

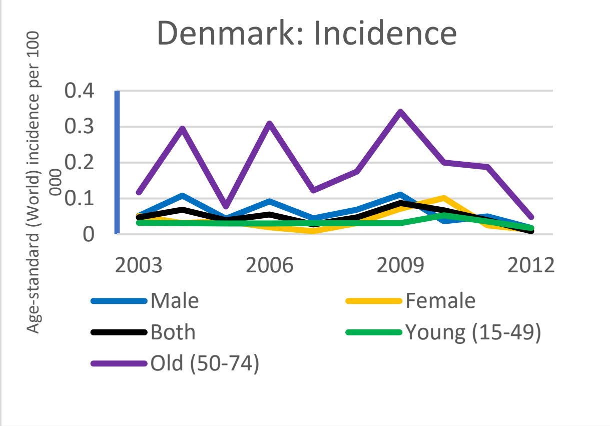

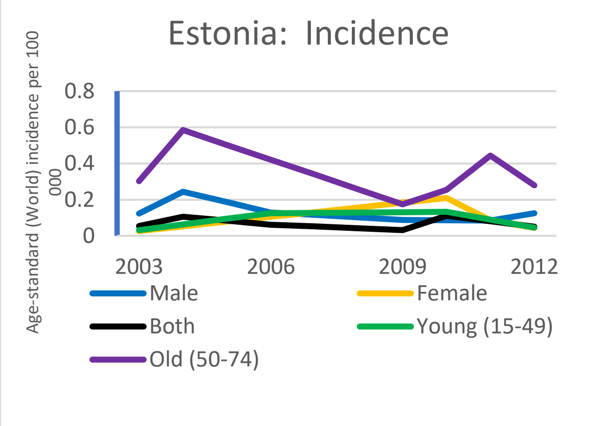

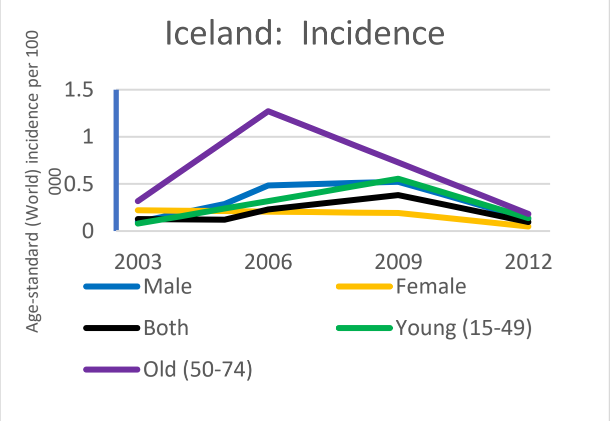

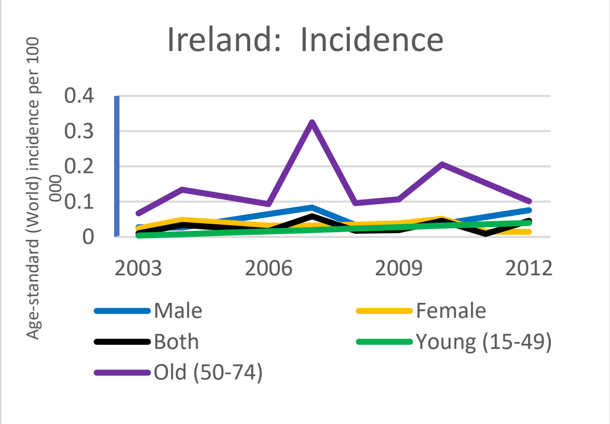

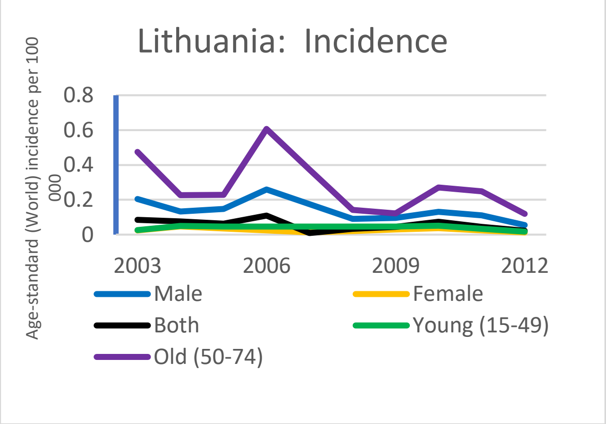

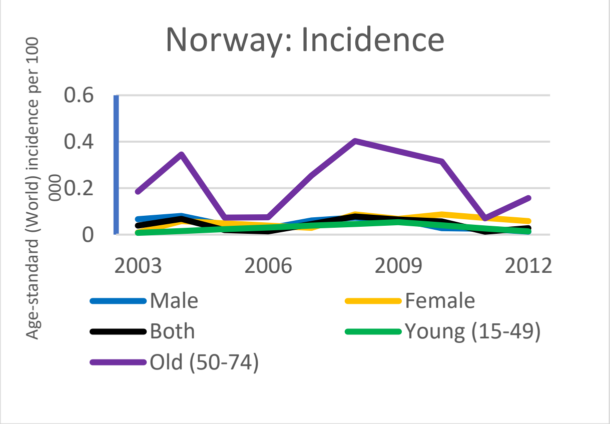

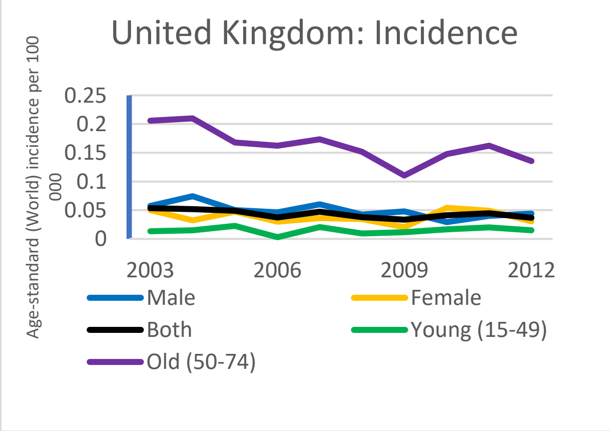

Western Europe

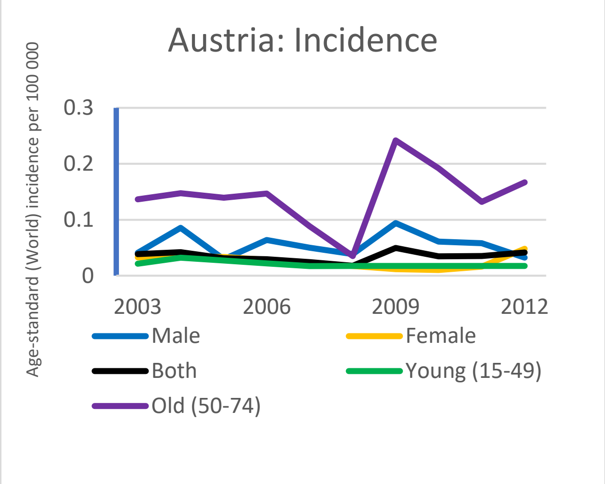

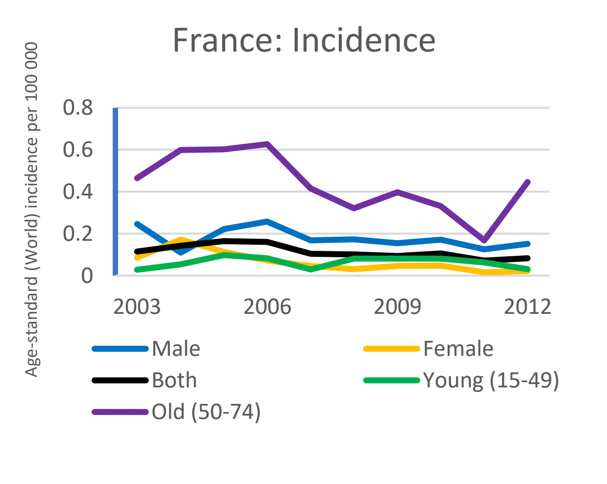

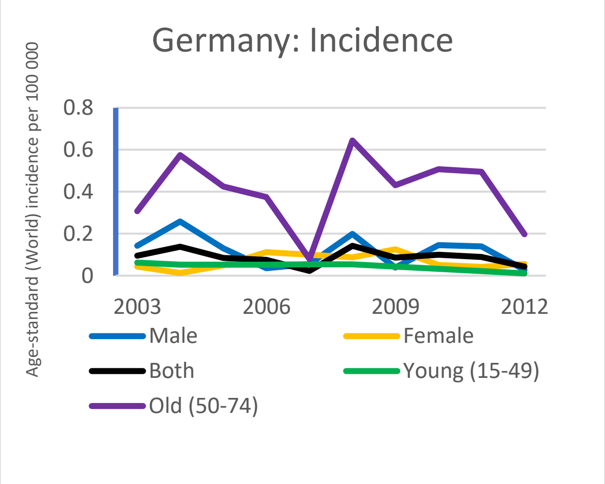

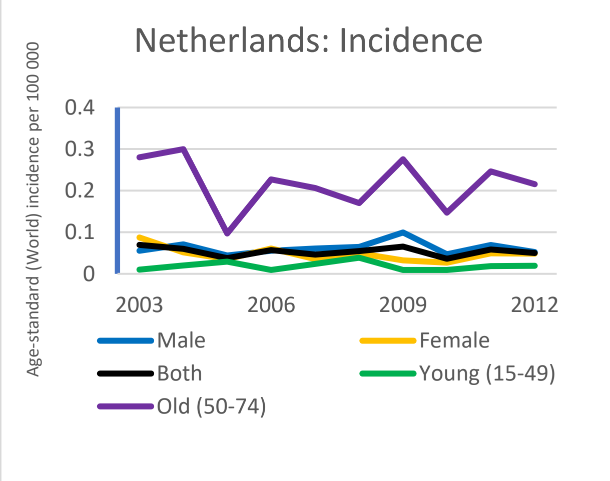

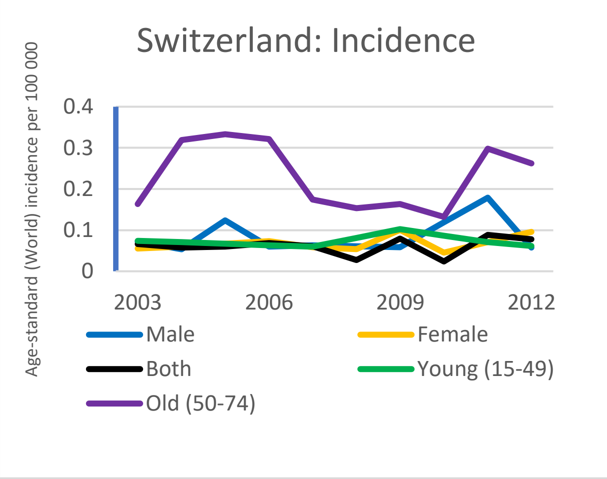

Southern Europe

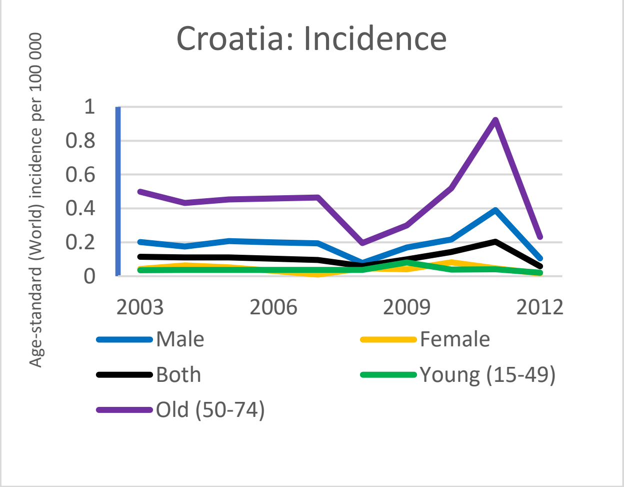

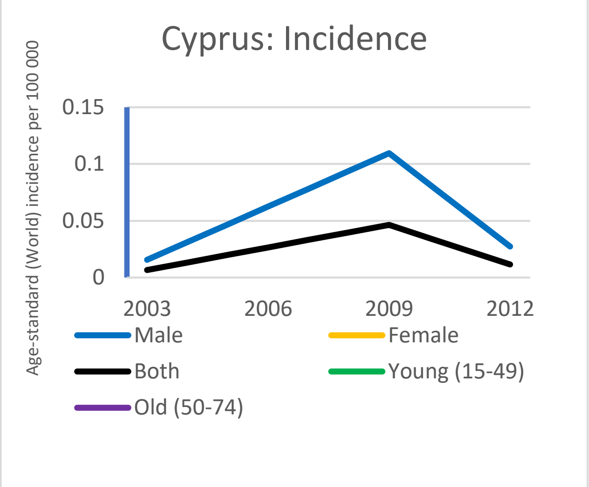

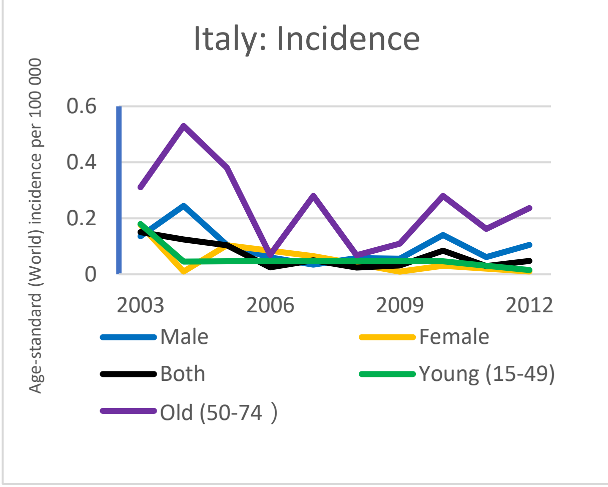

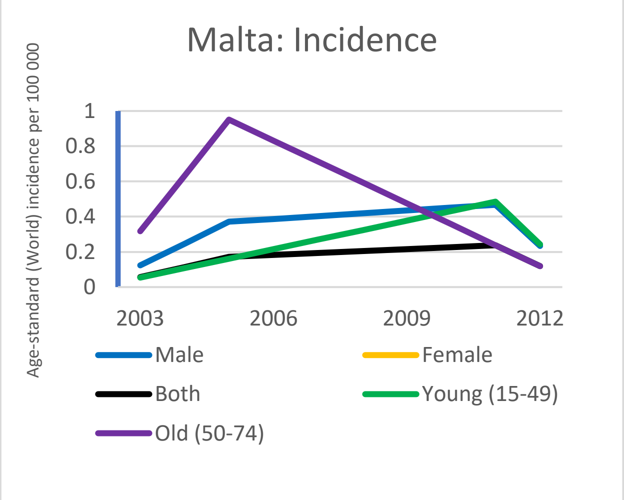

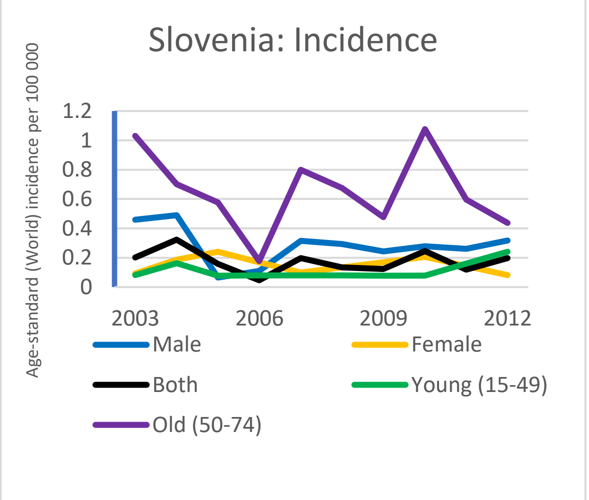

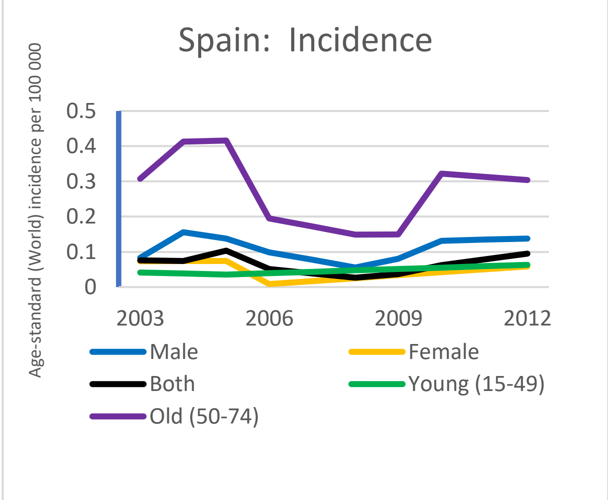

Eastern Europe

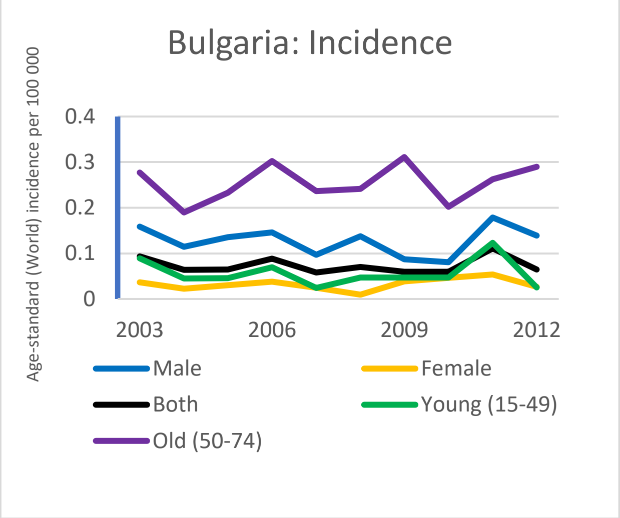

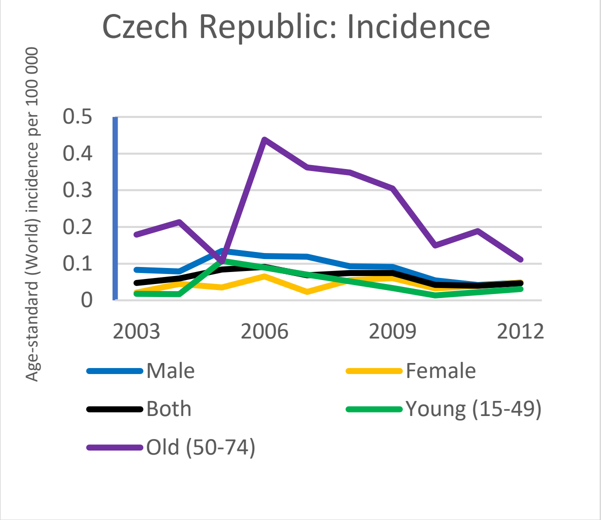

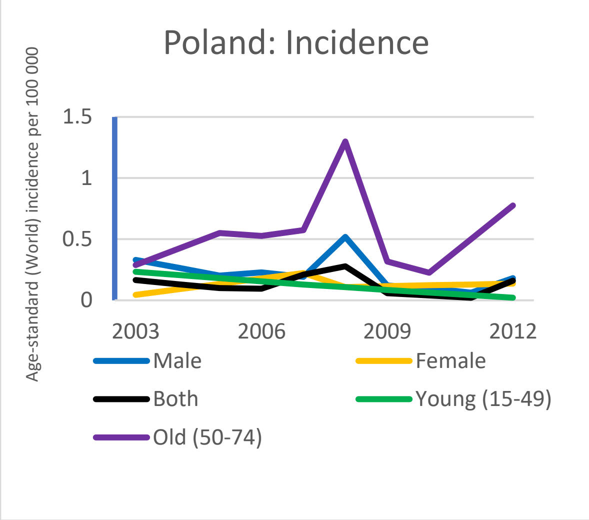

Africa

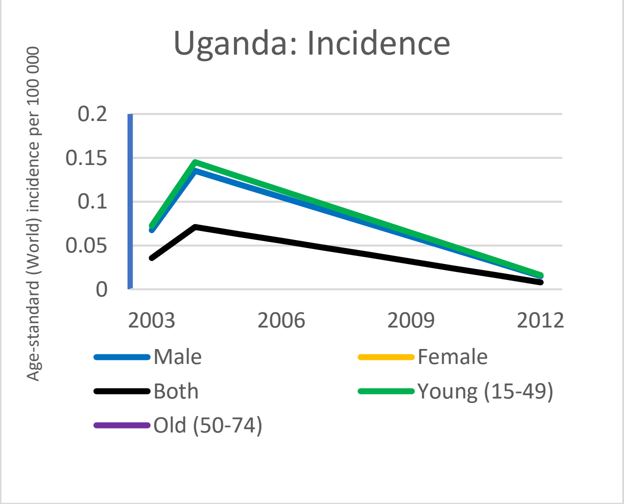

Supplementary Figure 2: The graphs of the joinpoint regression output

a.) Male

Asia

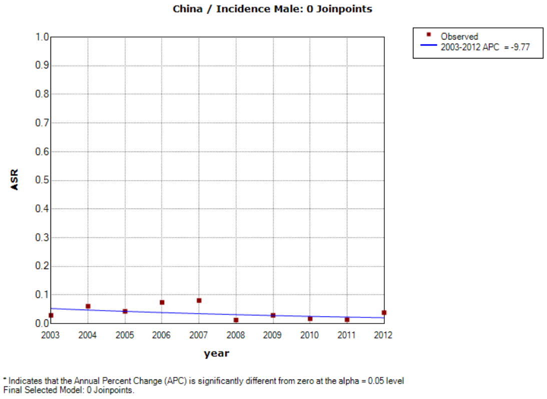

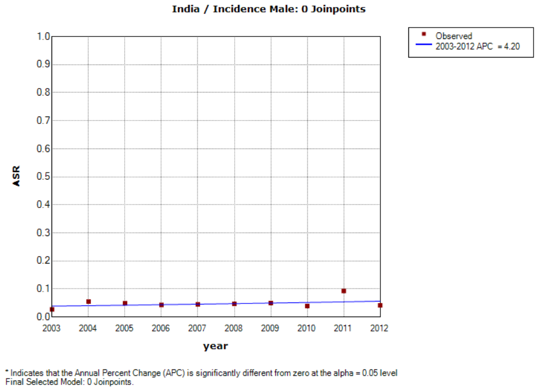

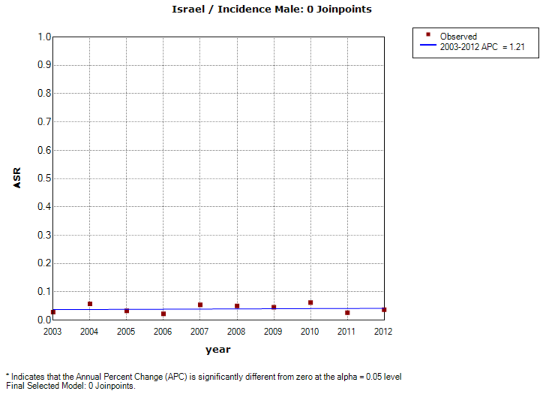

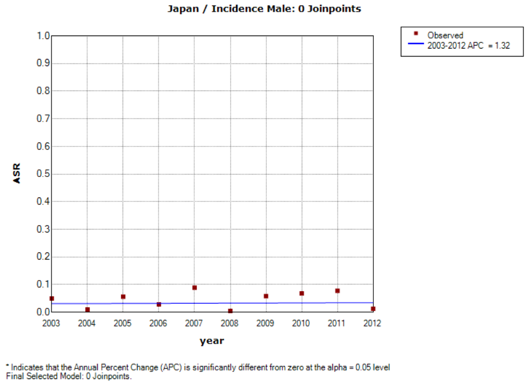

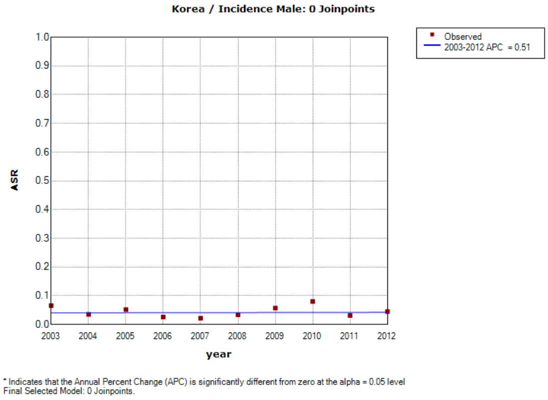

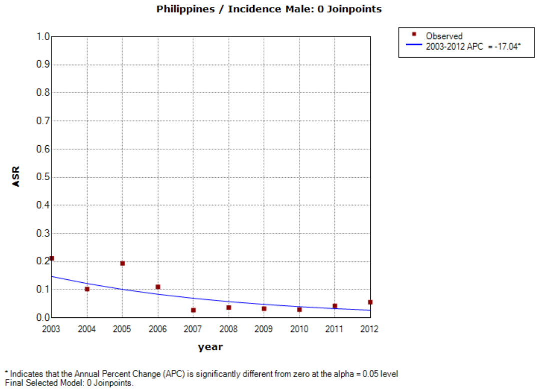

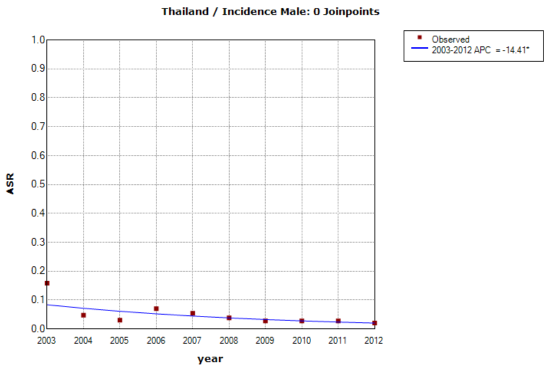

* Indicates that the Annual Percent Change (APC) is significantly different from zero at the alpha = 0.05 level
Final Selected Model: 0 Joinpoints.

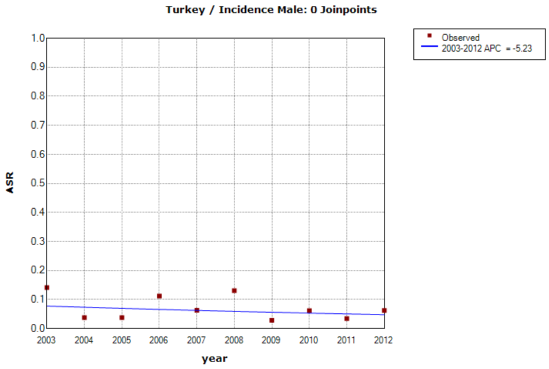

* Indicates that the Annual Percent Change (APC) is significantly different from zero at the alpha = 0.05 level
Final Selected Model: 0 Joinpoints.

Oceania

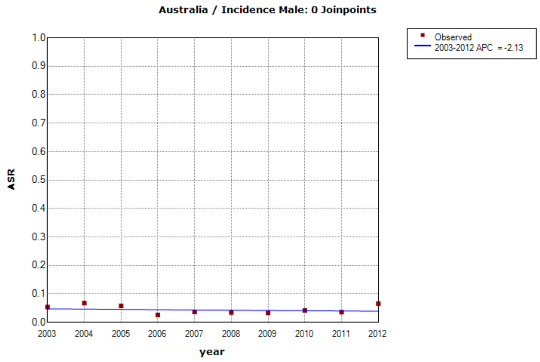

* Indicates that the Annual Percent Change (APC) is significantly different from zero at the alpha = 0.05 level
Final Selected Model: 0 Joinpoints.

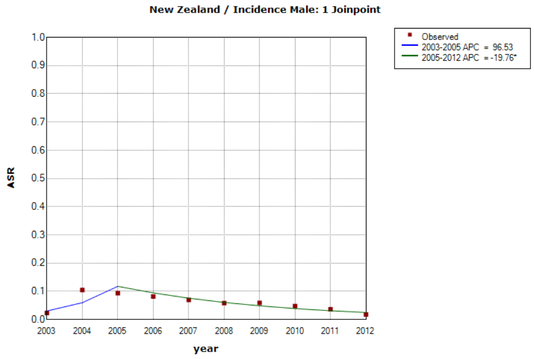

* Indicates that the Annual Percent Change (APC) is significantly different from zero at the alpha = 0.05 level
Final Selected Model: 1 Joinpoint.

Northern America

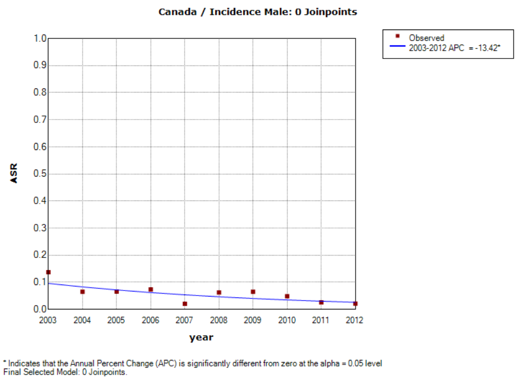

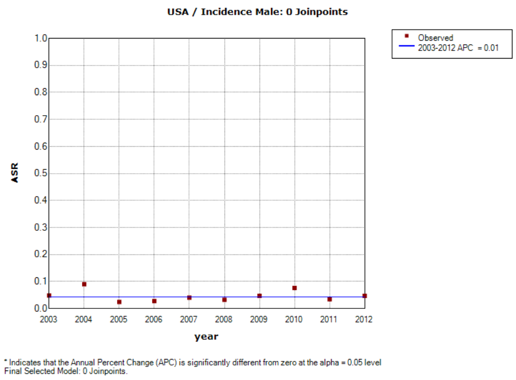

Southern America

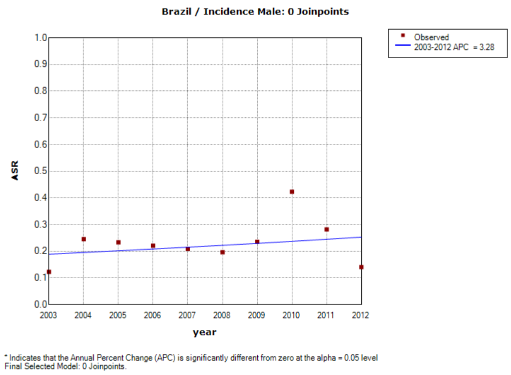

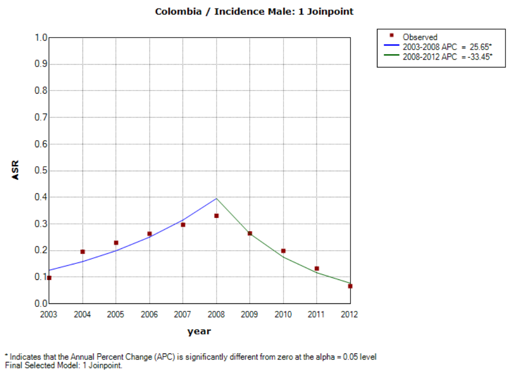

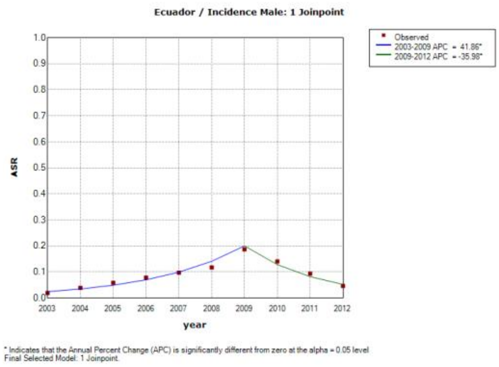

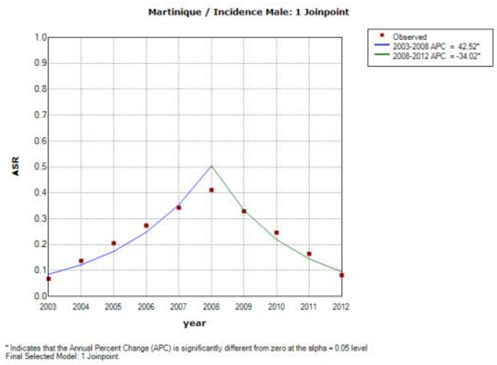

Northern Europe

Denmark / Incidence Male: 0 Joinpoints

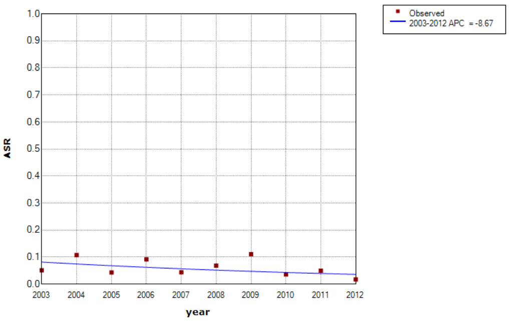

* Indicates that the Annual Percent Change (APC) is significantly different from zero at the alpha = 0.05 level
Final Selected Model: 0 Joinpoints.

Estonia / Incidence Male: 0 Joinpoints

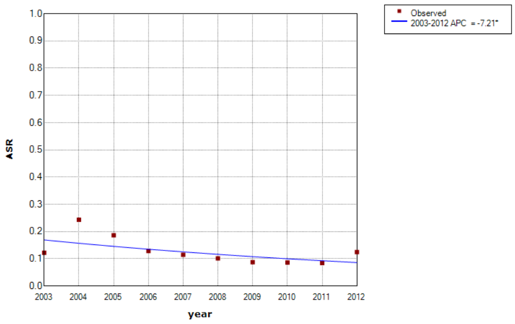

* Indicates that the Annual Percent Change (APC) is significantly different from zero at the alpha = 0.05 level
Final Selected Model: 0 Joinpoints.

Iceland / Incidence Male: 1 Joinpoint

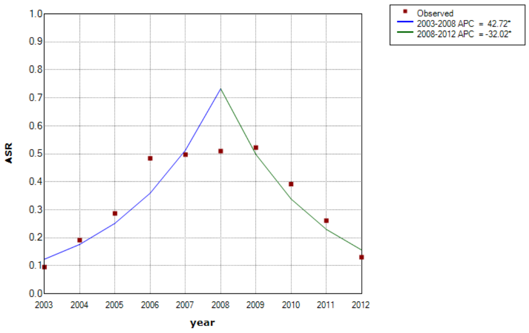

* Indicates that the Annual Percent Change (APC) is significantly different from zero at the alpha = 0.05 level
Final Selected Model: 1 Joinpoint.

Ireland / Incidence Male: 0 Joinpoints

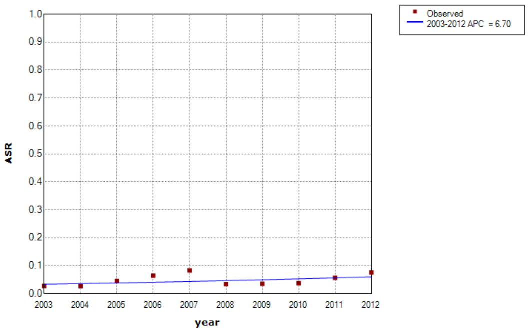

* Indicates that the Annual Percent Change (APC) is significantly different from zero at the alpha = 0.05 level
Final Selected Model: 0 Joinpoints.

Lithuania / Incidence Male: 0 Joinpoints

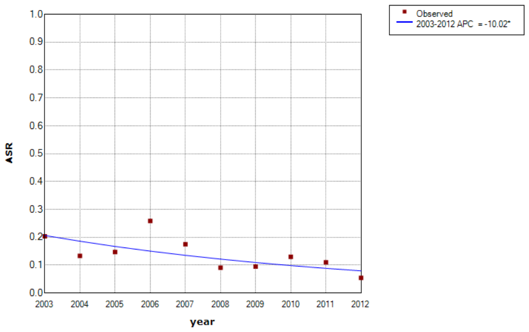

* Indicates that the Annual Percent Change (APC) is significantly different from zero at the alpha = 0.05 level
Final Selected Model: 0 Joinpoints.

Norway / Incidence Male: 0 Joinpoints

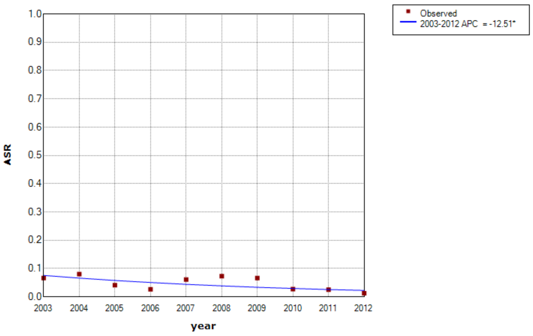

* Indicates that the Annual Percent Change (APC) is significantly different from zero at the alpha = 0.05 level
Final Selected Model: 0 Joinpoints.

United Kingdom / Incidence Male: 0 Joinpoints

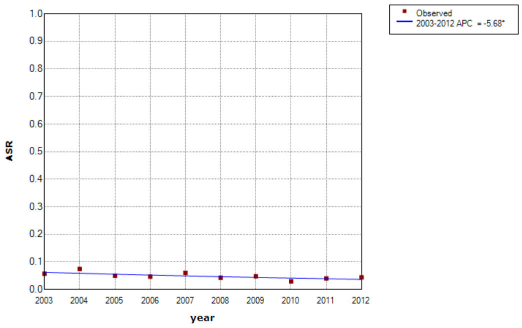

* Indicates that the Annual Percent Change (APC) is significantly different from zero at the alpha = 0.05 level
Final Selected Model: 0 Joinpoints.

Western Europe

Austria / Incidence Male: 0 Joinpoints

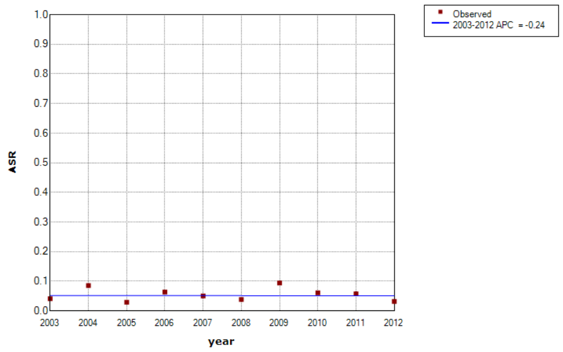

* Indicates that the Annual Percent Change (APC) is significantly different from zero at the alpha = 0.05 level
Final Selected Model: 0 Joinpoints.

France / Incidence Male: 0 Joinpoints

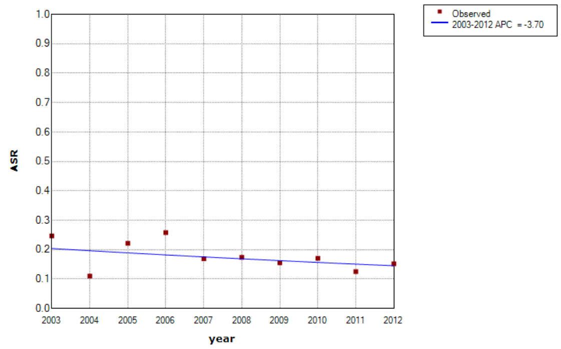

* Indicates that the Annual Percent Change (APC) is significantly different from zero at the alpha = 0.05 level
Final Selected Model: 0 Joinpoints.

Germany / Incidence Male: 0 Joinpoints

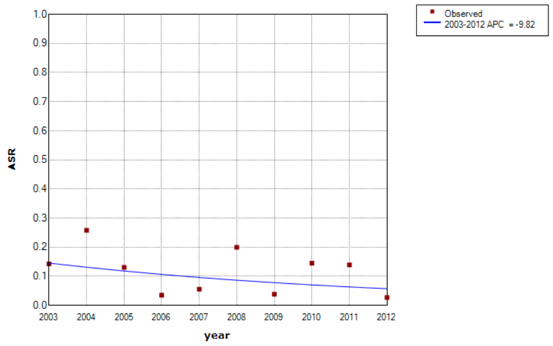

* Indicates that the Annual Percent Change (APC) is significantly different from zero at the alpha = 0.05 level
Final Selected Model: 0 Joinpoints.

Netherlands / Incidence Male: 0 Joinpoints

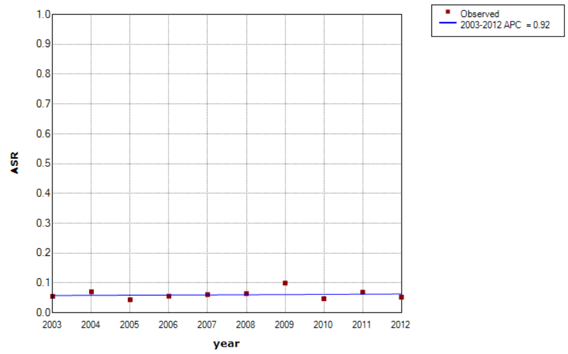

* Indicates that the Annual Percent Change (APC) is significantly different from zero at the alpha = 0.05 level
Final Selected Model: 0 Joinpoints.

Switzerland / Incidence Male: 0 Joinpoints

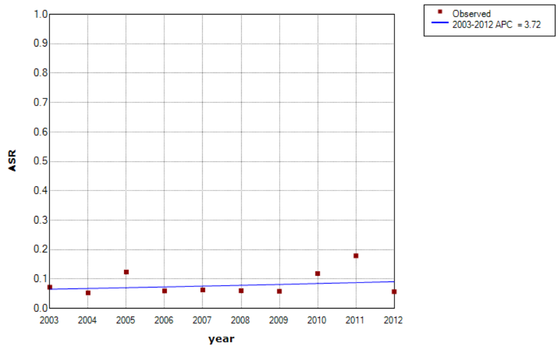

* Indicates that the Annual Percent Change (APC) is significantly different from zero at the alpha = 0.05 level
Final Selected Model: 0 Joinpoints.

Southern Europe

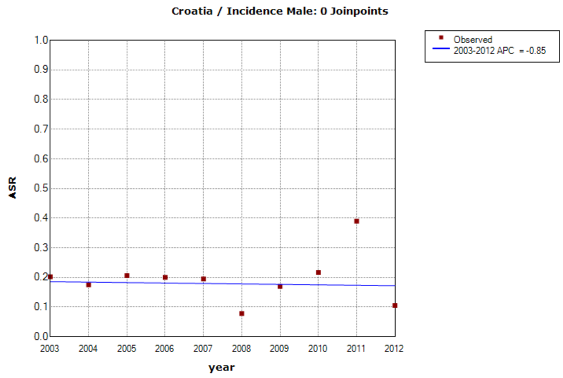

* Indicates that the Annual Percent Change (APC) is significantly different from zero at the alpha = 0.05 level
Final Selected Model: 0 Joinpoints.

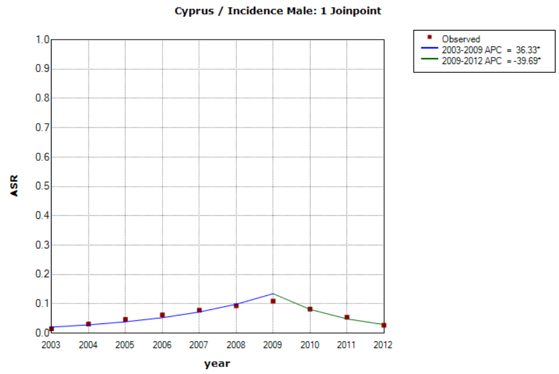

* Indicates that the Annual Percent Change (APC) is significantly different from zero at the alpha = 0.05 level
Final Selected Model: 1 Joinpoint.

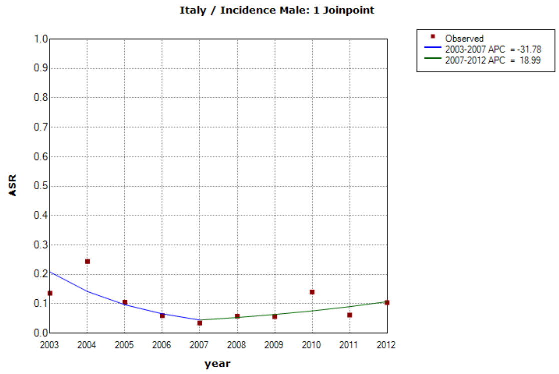

* Indicates that the Annual Percent Change (APC) is significantly different from zero at the alpha = 0.05 level
Final Selected Model: 1 Joinpoint.

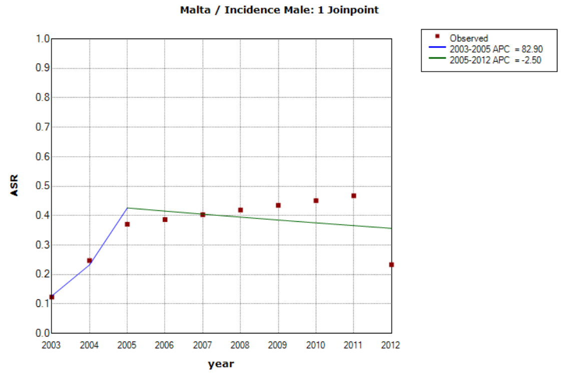

* Indicates that the Annual Percent Change (APC) is significantly different from zero at the alpha = 0.05 level
Final Selected Model: 1 Joinpoint.

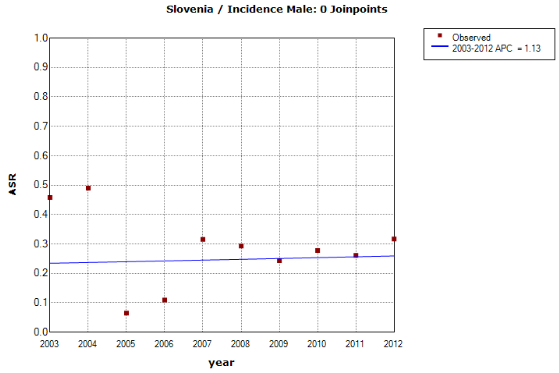

* Indicates that the Annual Percent Change (APC) is significantly different from zero at the alpha = 0.05 level
Final Selected Model: 0 Joinpoints.

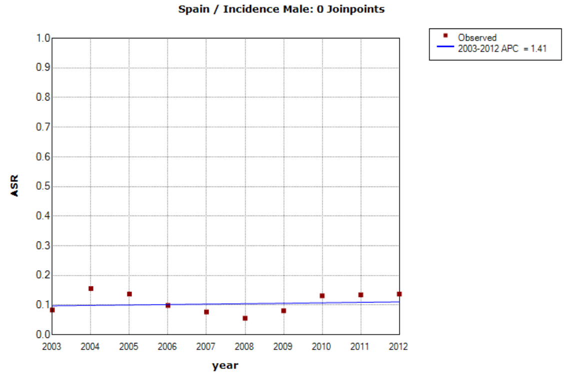

* Indicates that the Annual Percent Change (APC) is significantly different from zero at the alpha = 0.05 level
Final Selected Model: 0 Joinpoints.

Eastern Europe

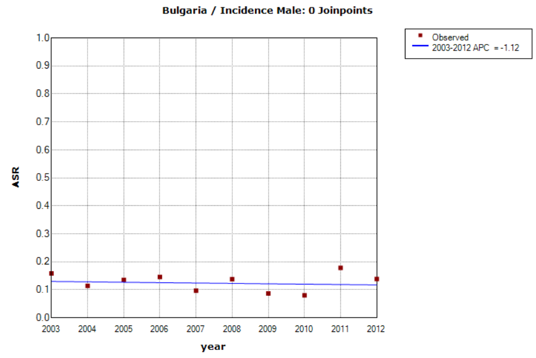

* Indicates that the Annual Percent Change (APC) is significantly different from zero at the alpha = 0.05 level
Final Selected Model: 0 Joinpoints.

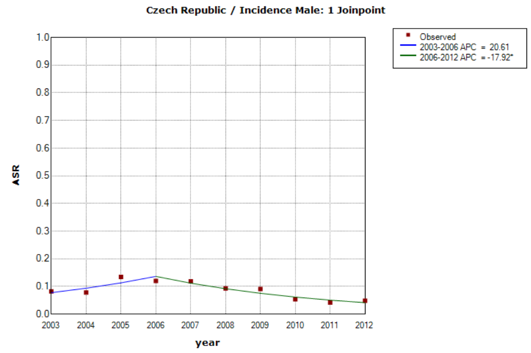

* Indicates that the Annual Percent Change (APC) is significantly different from zero at the alpha = 0.05 level
Final Selected Model: 1 Joinpoint.

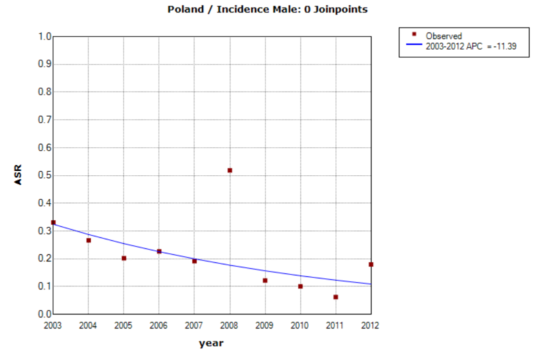

* Indicates that the Annual Percent Change (APC) is significantly different from zero at the alpha = 0.05 level
Final Selected Model: 0 Joinpoints.

Africa

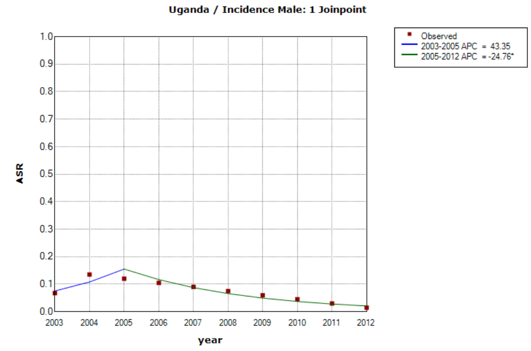

* Indicates that the Annual Percent Change (APC) is significantly different from zero at the alpha = 0.05 level
Final Selected Model: 1 Joinpoint.

b.) Female

Asia

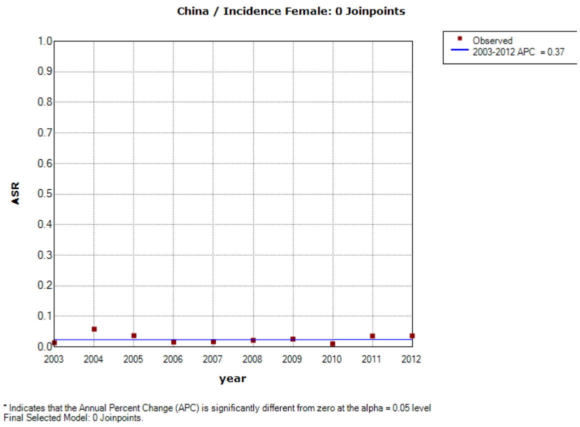

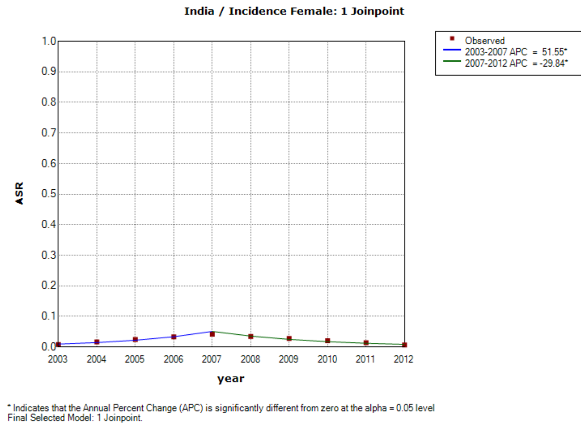

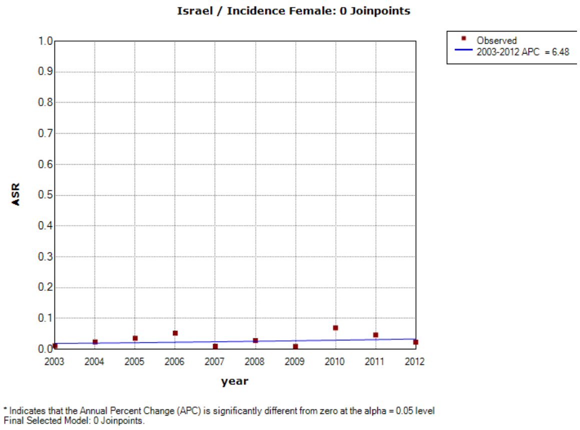

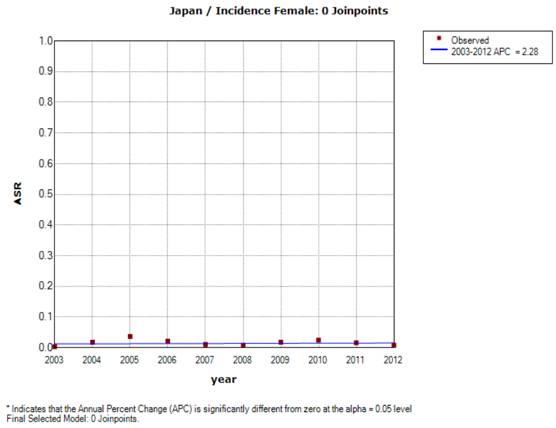

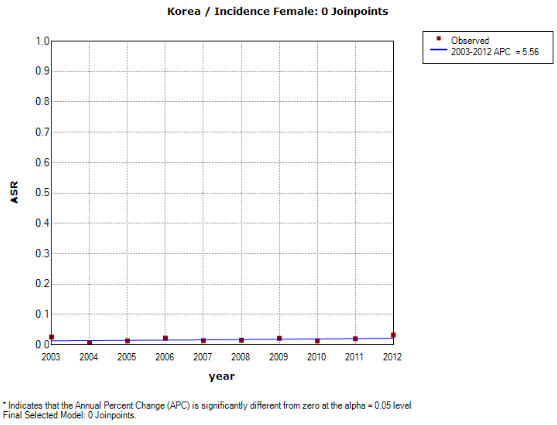

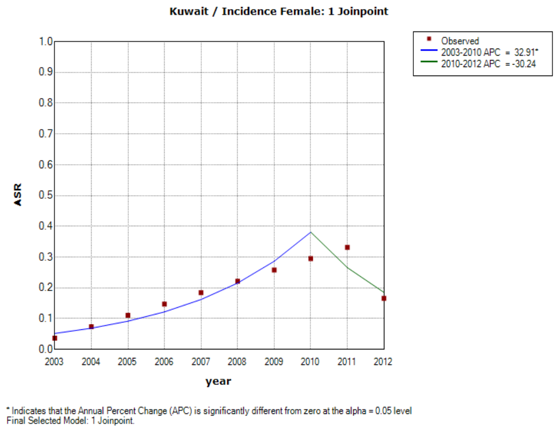

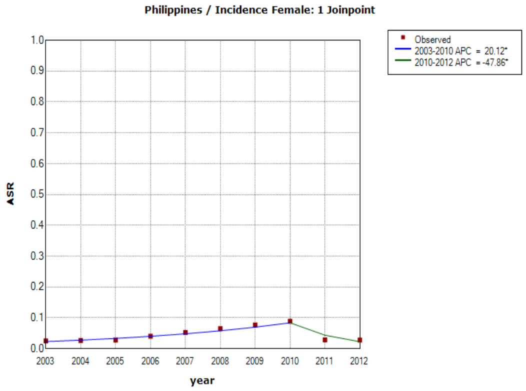

* Indicates that the Annual Percent Change (APC) is significantly different from zero at the alpha = 0.05 level
Final Selected Model: 1 Joinpoint.

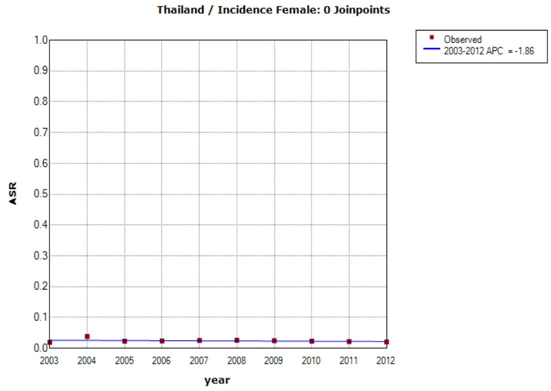

* Indicates that the Annual Percent Change (APC) is significantly different from zero at the alpha = 0.05 level
Final Selected Model: 0 Joinpoints.

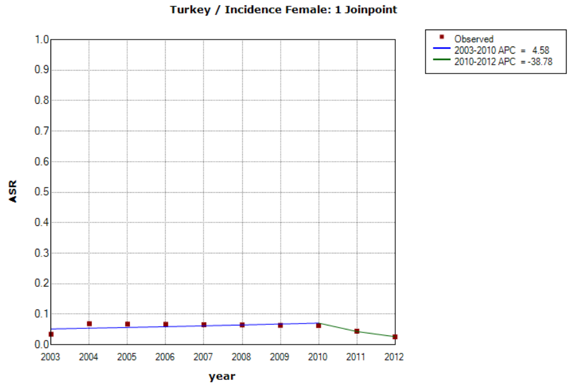

* Indicates that the Annual Percent Change (APC) is significantly different from zero at the alpha = 0.05 level
Final Selected Model: 1 Joinpoint.

Oceania

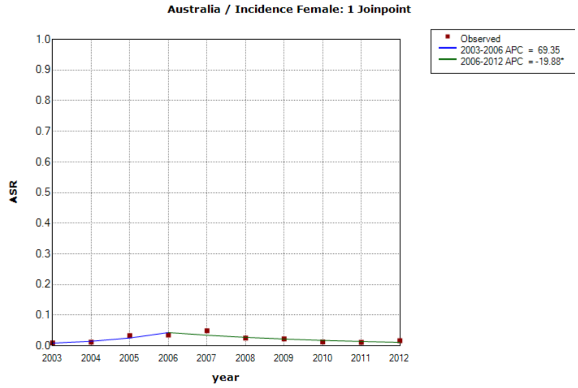

* Indicates that the Annual Percent Change (APC) is significantly different from zero at the alpha = 0.05 level
Final Selected Model: 1 Joinpoint.

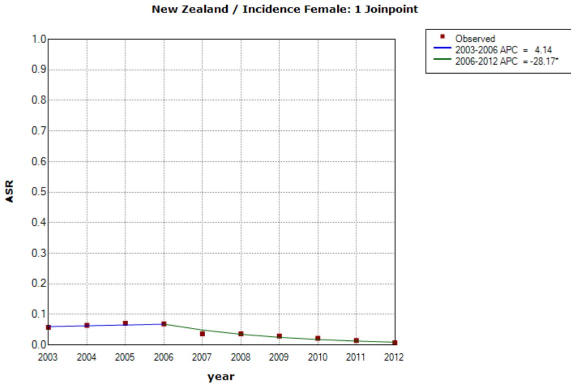

* Indicates that the Annual Percent Change (APC) is significantly different from zero at the alpha = 0.05 level
Final Selected Model: 1 Joinpoint.

Northern America

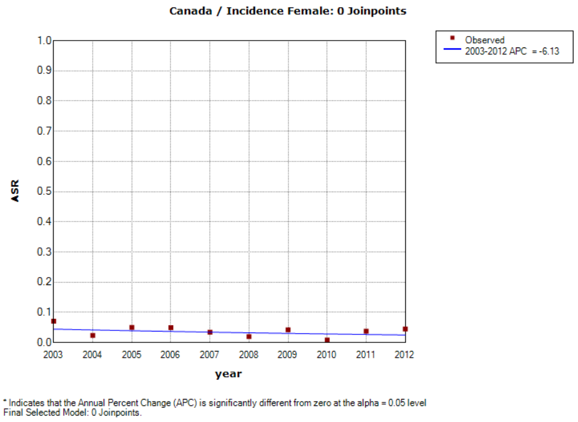

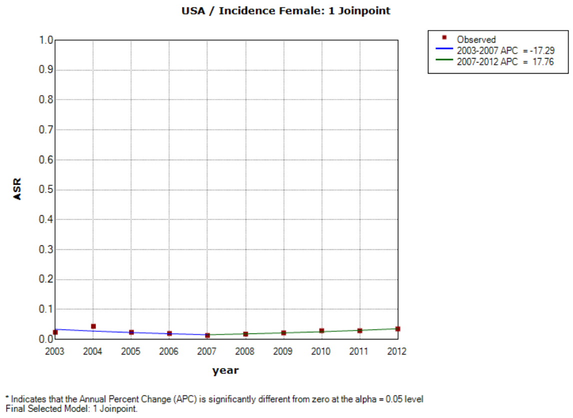

Southern America

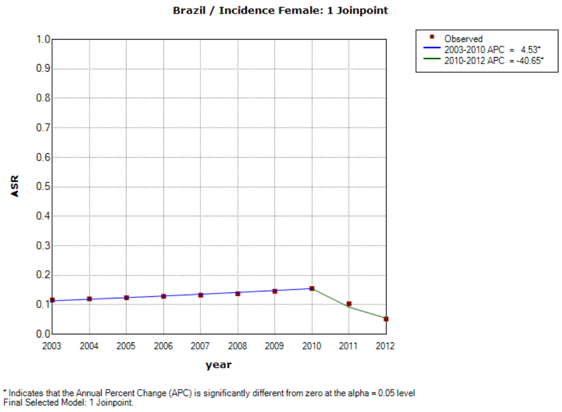

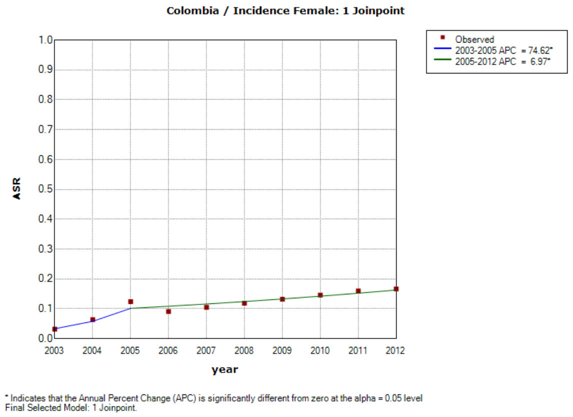

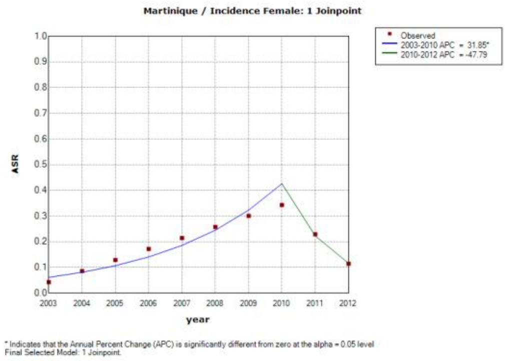

Northern Europe

Denmark / Incidence Female: 0 Joinpoints

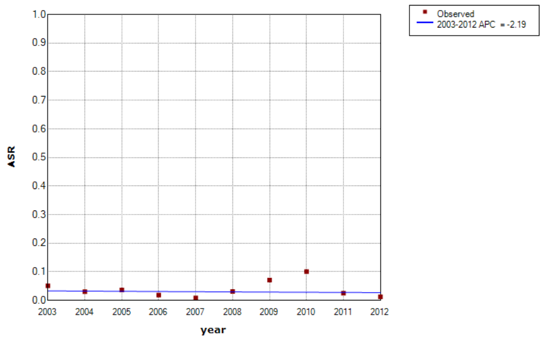

* Indicates that the Annual Percent Change (APC) is significantly different from zero at the alpha = 0.05 level
Final Selected Model: 0 Joinpoints.

Estonia / Incidence Female: 1 Joinpoint

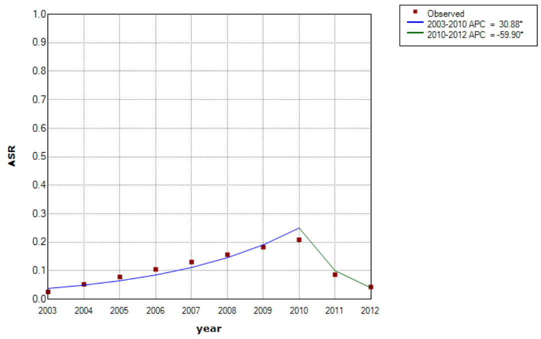

* Indicates that the Annual Percent Change (APC) is significantly different from zero at the alpha = 0.05 level
Final Selected Model: 1 Joinpoint.

Iceland / Incidence Female: 1 Joinpoint

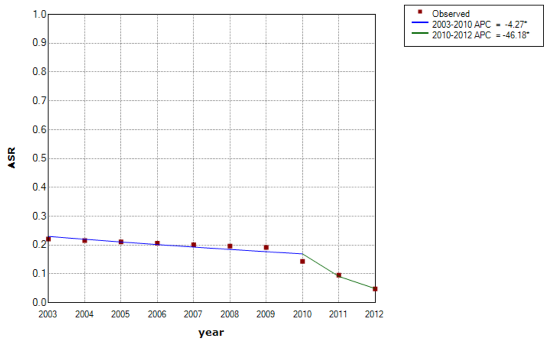

* Indicates that the Annual Percent Change (APC) is significantly different from zero at the alpha = 0.05 level
Final Selected Model: 1 Joinpoint.

Ireland / Incidence Female: 1 Joinpoint

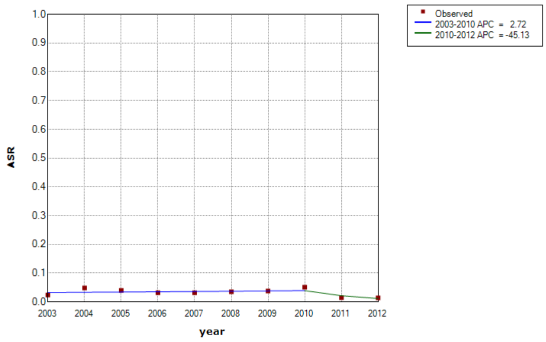

* Indicates that the Annual Percent Change (APC) is significantly different from zero at the alpha = 0.05 level
Final Selected Model: 1 Joinpoint.

Lithuania / Incidence Female: 0 Joinpoints

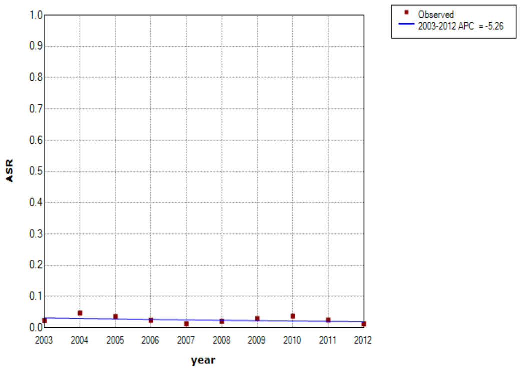

* Indicates that the Annual Percent Change (APC) is significantly different from zero at the alpha = 0.05 level
Final Selected Model: 0 Joinpoints.

Norway / Incidence Female: 0 Joinpoints

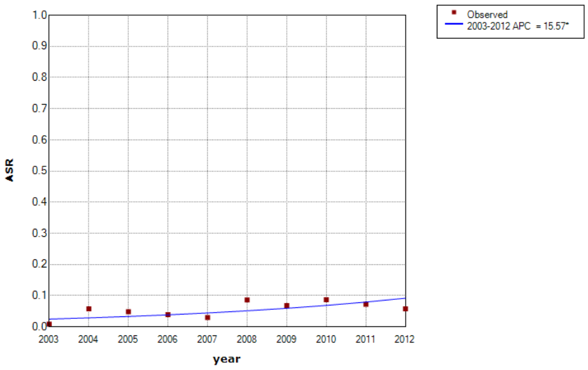

* Indicates that the Annual Percent Change (APC) is significantly different from zero at the alpha = 0.05 level
Final Selected Model: 0 Joinpoints.

United Kingdom / Incidence Female: 0 Joinpoints

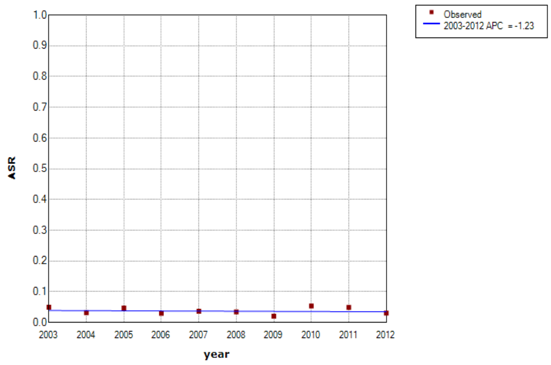

* Indicates that the Annual Percent Change (APC) is significantly different from zero at the alpha = 0.05 level
Final Selected Model: 0 Joinpoints.

Western Europe

Austria / Incidence Female: 1 Joinpoint

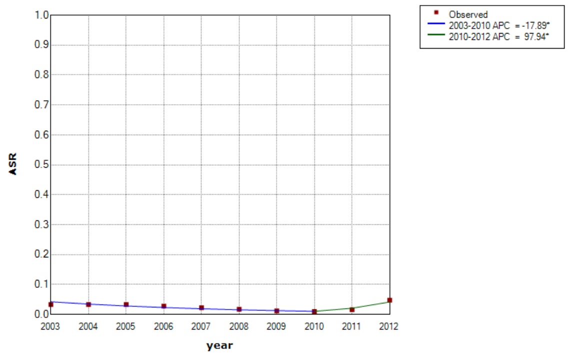

* Indicates that the Annual Percent Change (APC) is significantly different from zero at the alpha = 0.05 level
Final Selected Model: 1 Joinpoint.

France / Incidence Female: 0 Joinpoints

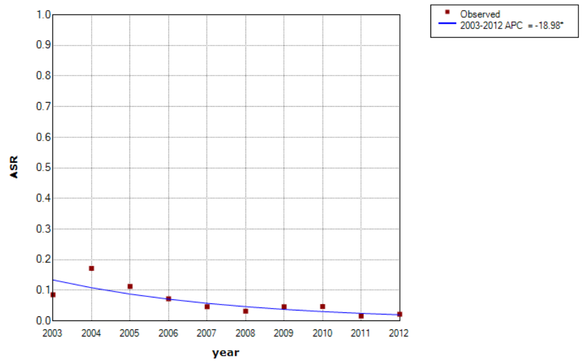

* Indicates that the Annual Percent Change (APC) is significantly different from zero at the alpha = 0.05 level
Final Selected Model: 0 Joinpoints.

Germany / Incidence Female: 0 Joinpoints

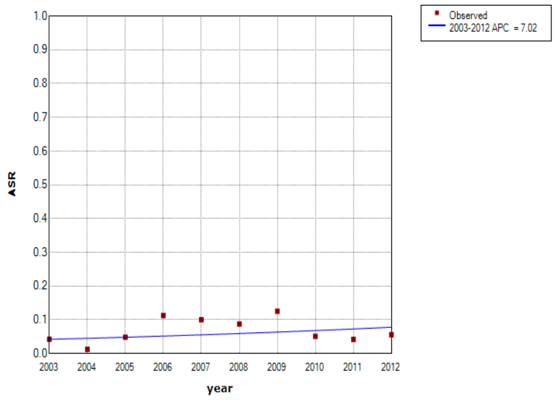

* Indicates that the Annual Percent Change (APC) is significantly different from zero at the alpha = 0.05 level
Final Selected Model: 0 Joinpoints.

Netherlands / Incidence Female: 0 Joinpoints

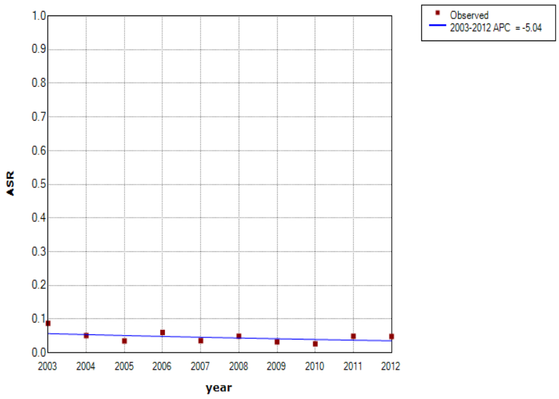

* Indicates that the Annual Percent Change (APC) is significantly different from zero at the alpha = 0.05 level
Final Selected Model: 0 Joinpoints.

Switzerland / Incidence Female: 0 Joinpoints

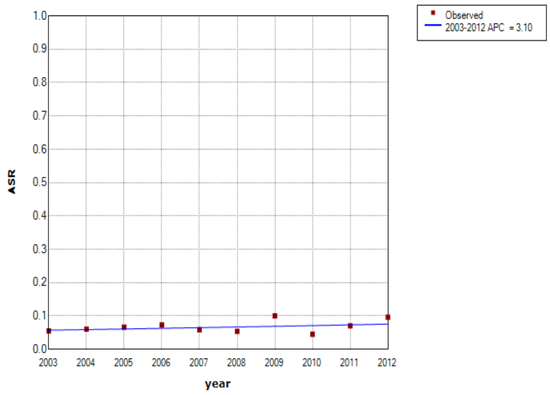

* Indicates that the Annual Percent Change (APC) is significantly different from zero at the alpha = 0.05 level
Final Selected Model: 0 Joinpoints.

Southern Europe

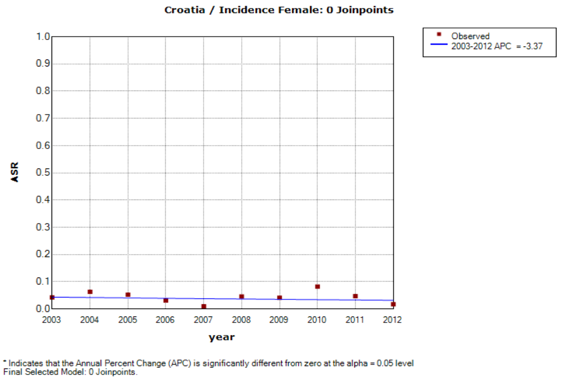

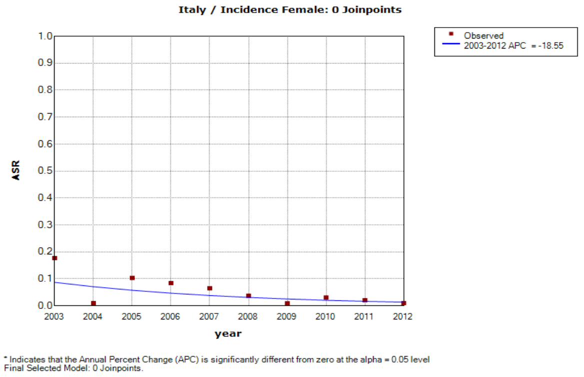

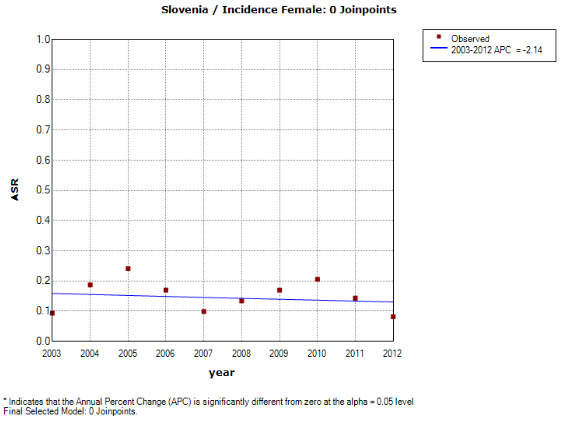

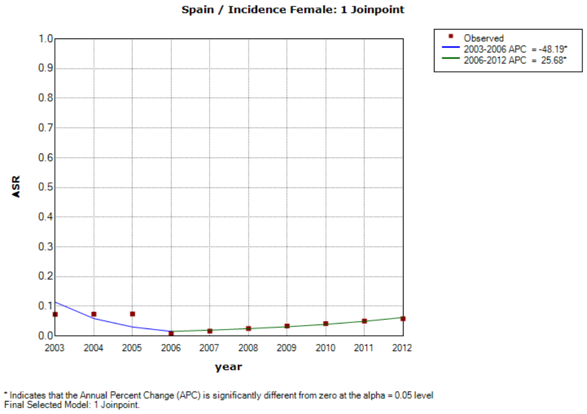

Eastern Europe

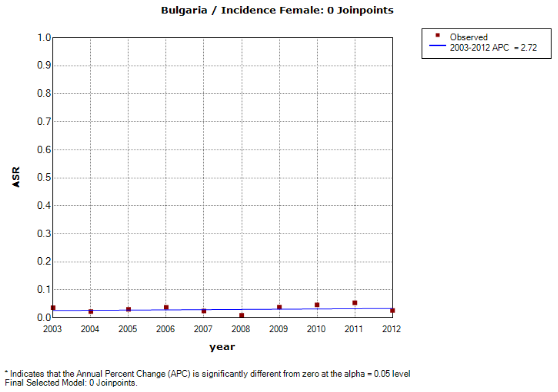

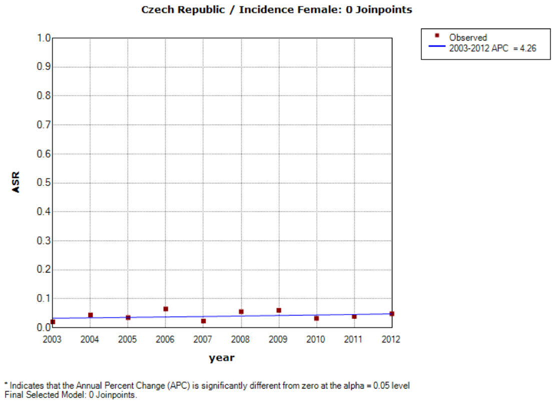

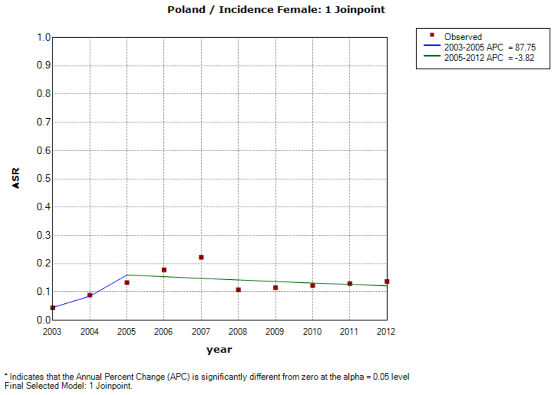

c.) Both

Asia

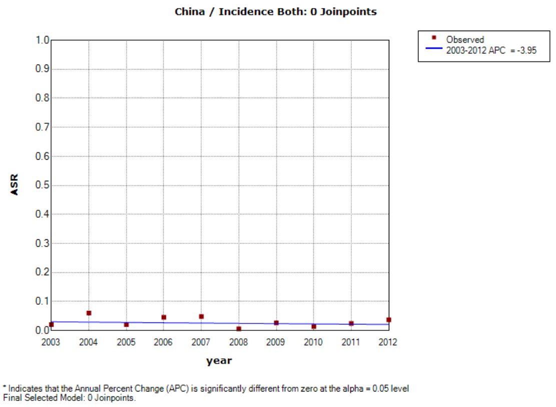
China / Incidence Both: 0 Joinpoints
Observed
2003-2012 APC = -3.95
ASR
year
* Indicates that the Annual Percent Change (APC) is significantly different from zero at the alpha = 0.05 level
Final Selected Model: 0 Joinpoints.

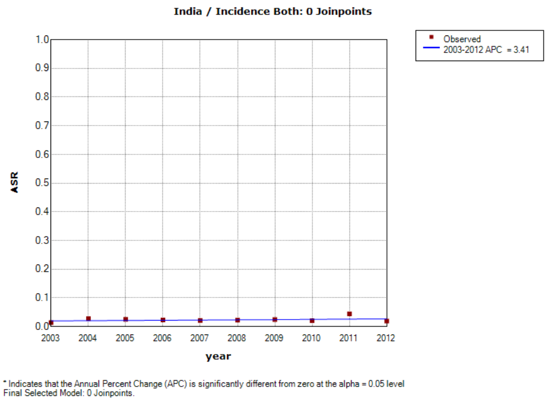
India / Incidence Both: 0 Joinpoints
Observed
2003-2012 APC = 3.41
ASR
year
* Indicates that the Annual Percent Change (APC) is significantly different from zero at the alpha = 0.05 level
Final Selected Model: 0 Joinpoints.

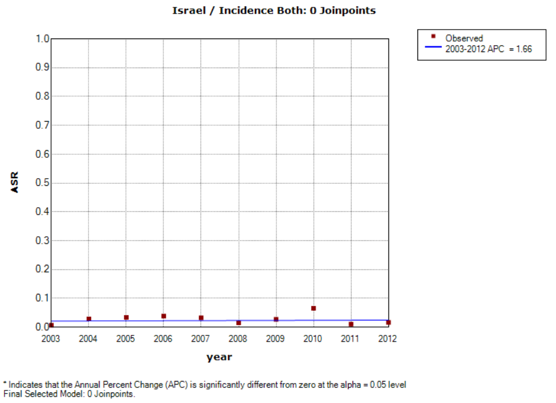
Israel / Incidence Both: 0 Joinpoints
Observed
2003-2012 APC = 1.66
ASR
year
* Indicates that the Annual Percent Change (APC) is significantly different from zero at the alpha = 0.05 level
Final Selected Model: 0 Joinpoints.

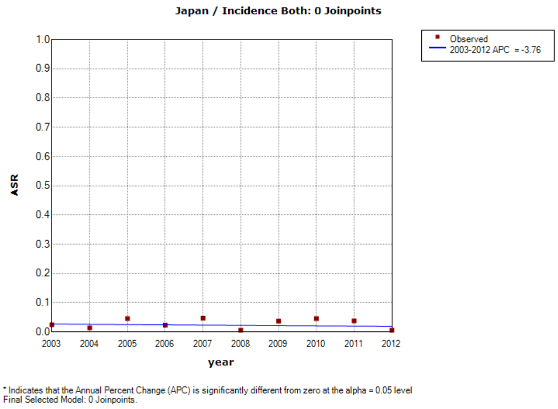
Japan / Incidence Both: 0 Joinpoints
Observed
2003-2012 APC = -3.76
ASR
year
* Indicates that the Annual Percent Change (APC) is significantly different from zero at the alpha = 0.05 level
Final Selected Model: 0 Joinpoints.

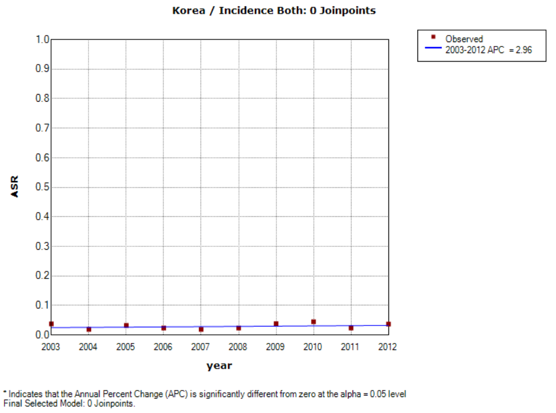
Korea / Incidence Both: 0 Joinpoints
Observed
2003-2012 APC = 2.96
ASR
year
* Indicates that the Annual Percent Change (APC) is significantly different from zero at the alpha = 0.05 level
Final Selected Model: 0 Joinpoints.

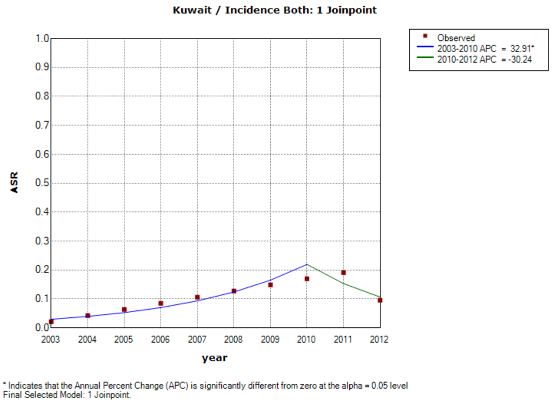
Kuwait / Incidence Both: 1 Joinpoint
Observed
2003-2010 APC = 32.91*
2010-2012 APC = -30.24
ASR
year
* Indicates that the Annual Percent Change (APC) is significantly different from zero at the alpha = 0.05 level
Final Selected Model: 1 Joinpoint.

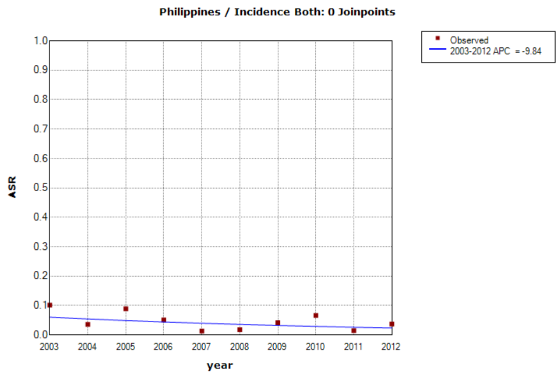

* Indicates that the Annual Percent Change (APC) is significantly different from zero at the alpha = 0.05 level
Final Selected Model: 0 Joinpoints.

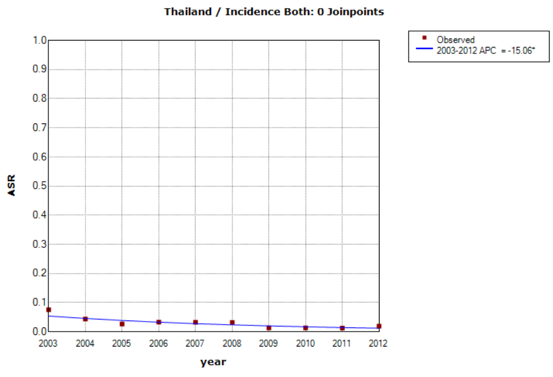

* Indicates that the Annual Percent Change (APC) is significantly different from zero at the alpha = 0.05 level
Final Selected Model: 0 Joinpoints.

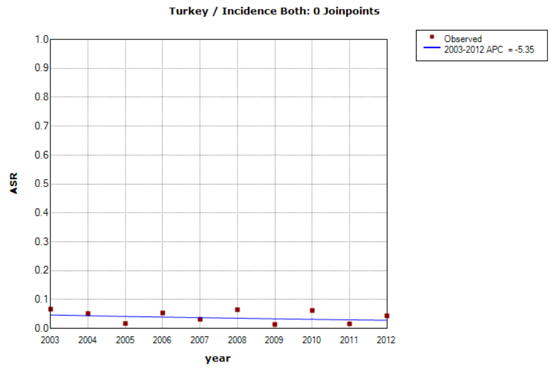

* Indicates that the Annual Percent Change (APC) is significantly different from zero at the alpha = 0.05 level
Final Selected Model: 0 Joinpoints.

Oceania

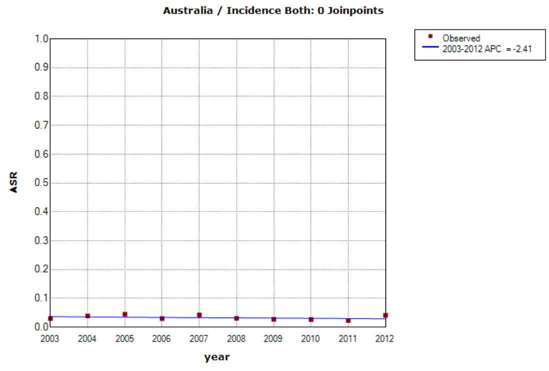

* Indicates that the Annual Percent Change (APC) is significantly different from zero at the alpha = 0.05 level
Final Selected Model: 0 Joinpoints.

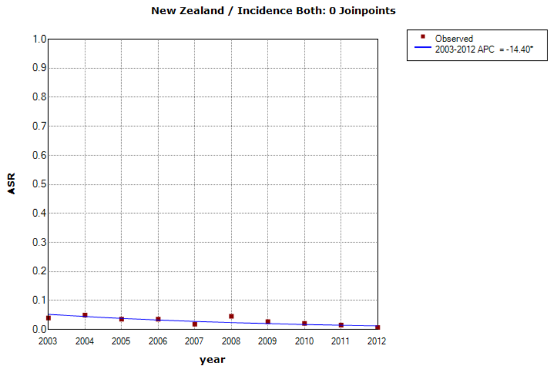

* Indicates that the Annual Percent Change (APC) is significantly different from zero at the alpha = 0.05 level
Final Selected Model: 0 Joinpoints.

Northern America

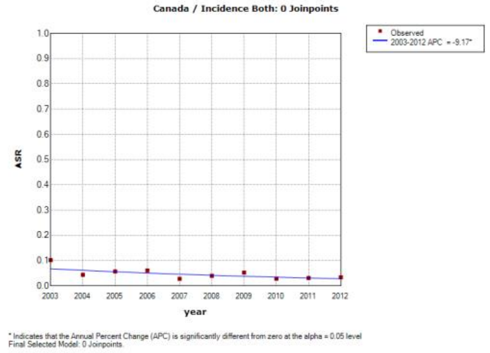

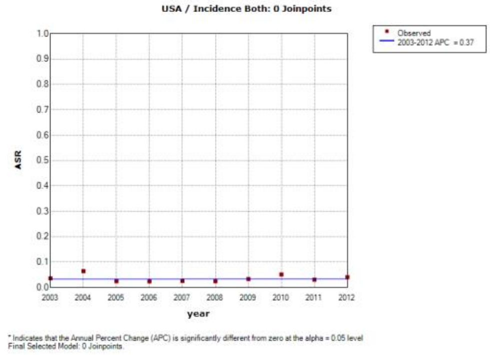

Southern America

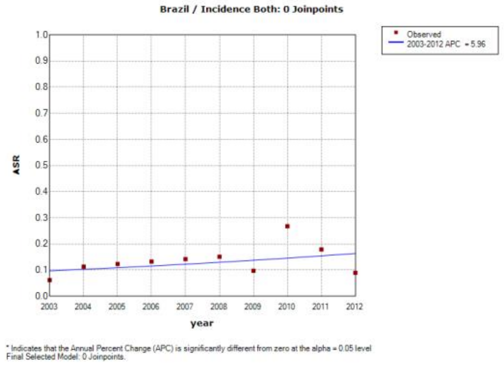

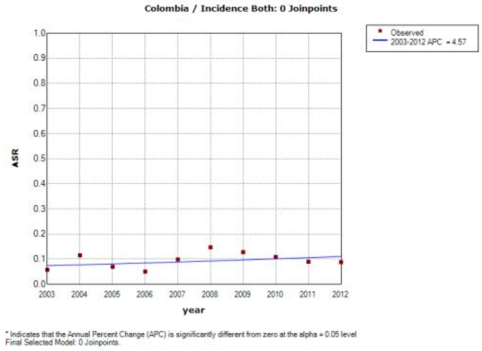

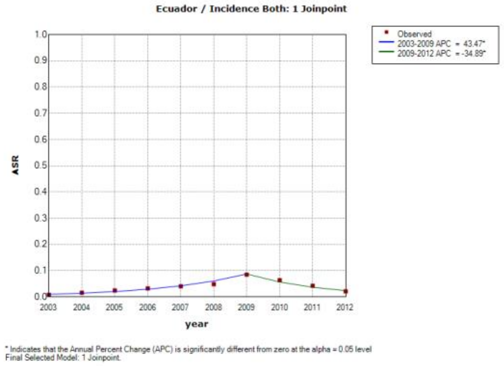

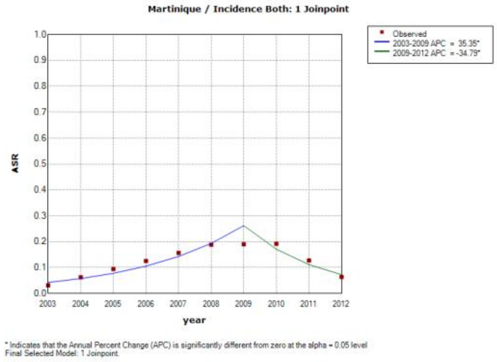

Northern Europe

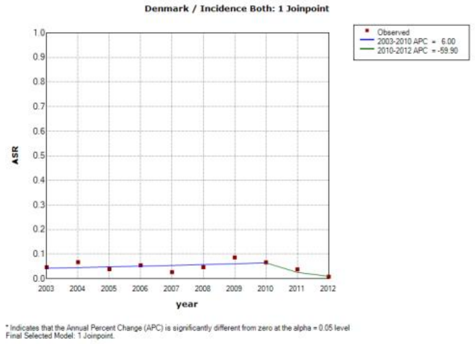

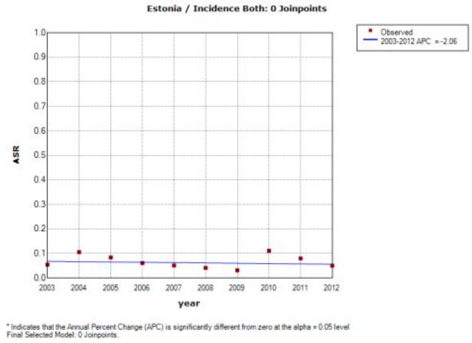

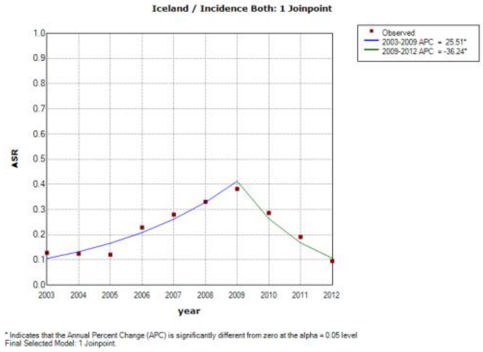

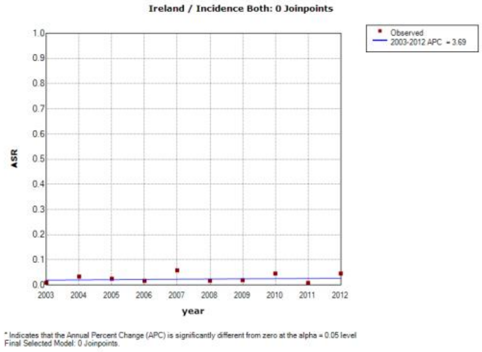

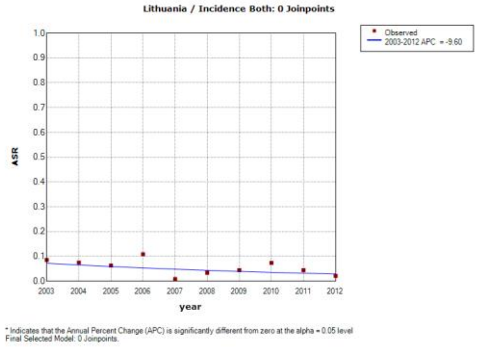

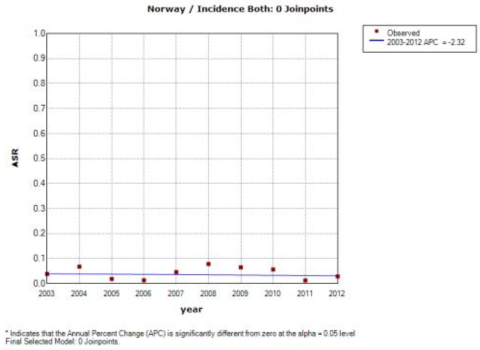

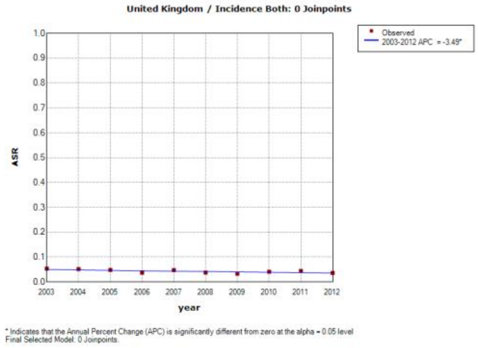

Western Europe

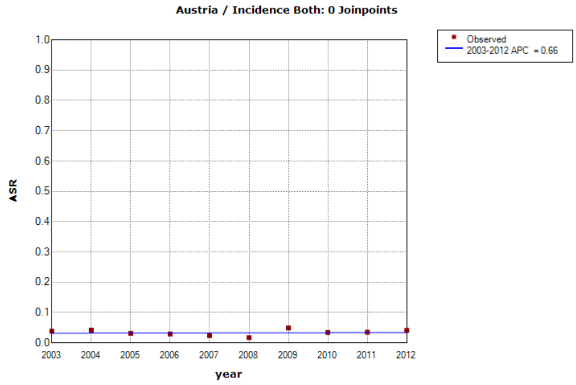

* Indicates that the Annual Percent Change (APC) is significantly different from zero at the alpha = 0.05 level
Final Selected Model: 0 Joinpoints.

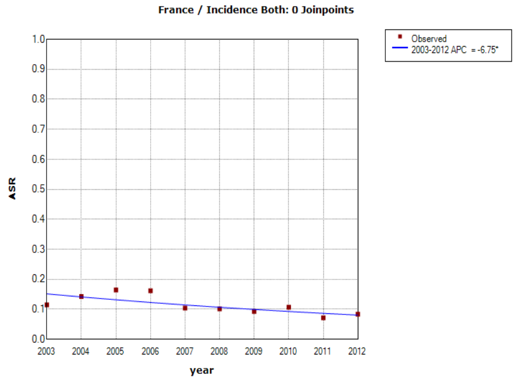

* Indicates that the Annual Percent Change (APC) is significantly different from zero at the alpha = 0.05 level
Final Selected Model: 0 Joinpoints.

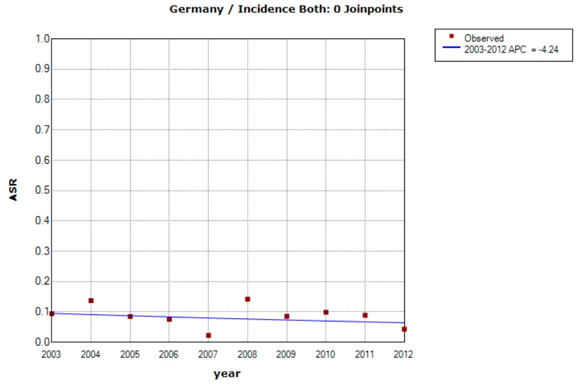

* Indicates that the Annual Percent Change (APC) is significantly different from zero at the alpha = 0.05 level
Final Selected Model: 0 Joinpoints.

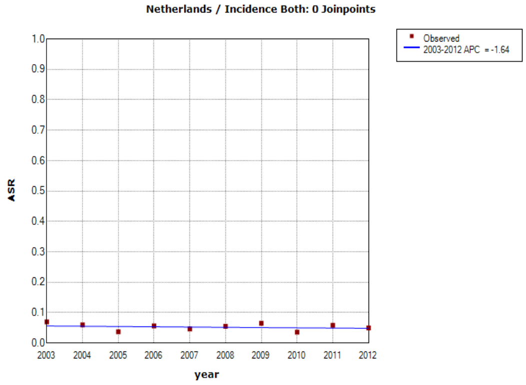

* Indicates that the Annual Percent Change (APC) is significantly different from zero at the alpha = 0.05 level
Final Selected Model: 0 Joinpoints.

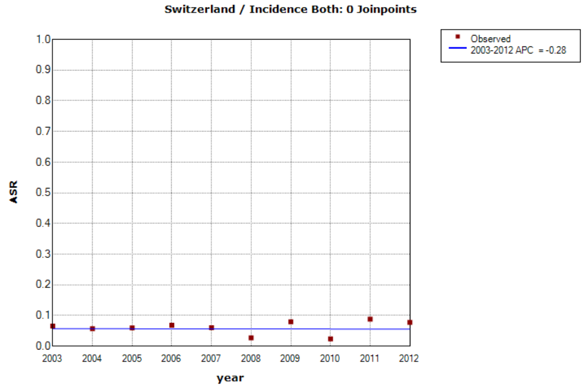

* Indicates that the Annual Percent Change (APC) is significantly different from zero at the alpha = 0.05 level
Final Selected Model: 0 Joinpoints.

Southern Europe

Croatia / Incidence Both: 0 Joinpoints

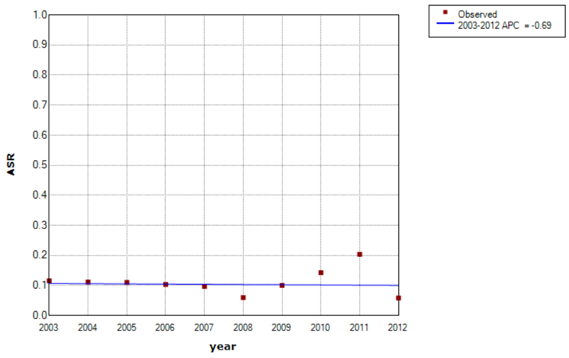

* Indicates that the Annual Percent Change (APC) is significantly different from zero at the alpha = 0.05 level
Final Selected Model: 0 Joinpoints.

Cyprus / Incidence Both: 1 Joinpoint

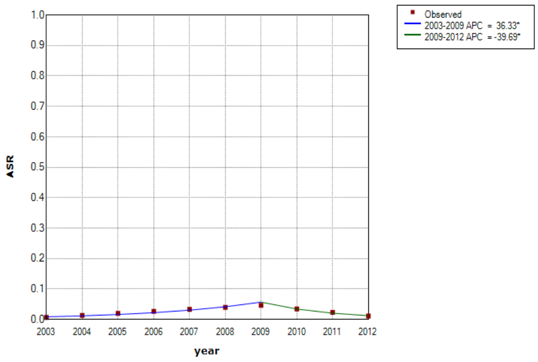

* Indicates that the Annual Percent Change (APC) is significantly different from zero at the alpha = 0.05 level
Final Selected Model: 1 Joinpoint.

Italy / Incidence Both: 0 Joinpoints

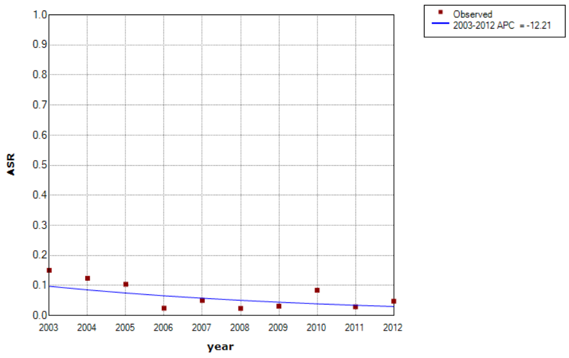

* Indicates that the Annual Percent Change (APC) is significantly different from zero at the alpha = 0.05 level
Final Selected Model: 0 Joinpoints.

Malta / Incidence Both: 1 Joinpoint

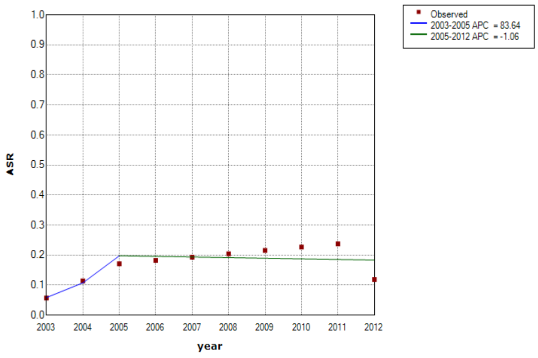

* Indicates that the Annual Percent Change (APC) is significantly different from zero at the alpha = 0.05 level
Final Selected Model: 1 Joinpoint.

Slovenia / Incidence Both: 0 Joinpoints

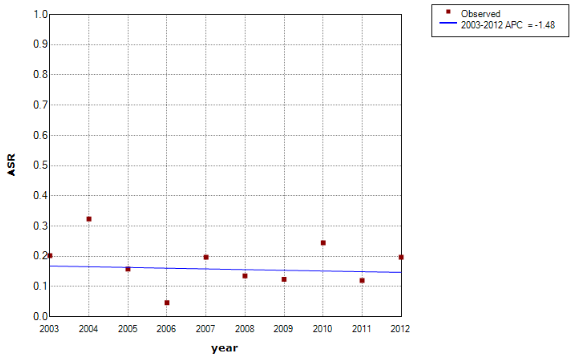

* Indicates that the Annual Percent Change (APC) is significantly different from zero at the alpha = 0.05 level
Final Selected Model: 0 Joinpoints.

Spain / Incidence Both: 1 Joinpoint

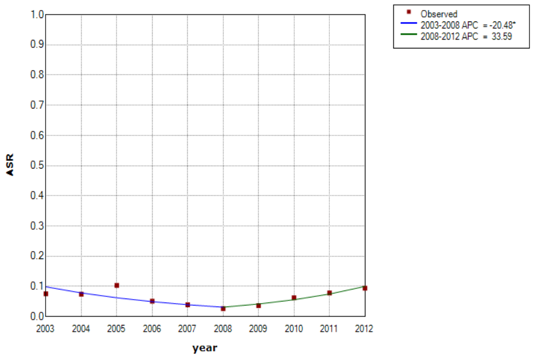

* Indicates that the Annual Percent Change (APC) is significantly different from zero at the alpha = 0.05 level
Final Selected Model: 1 Joinpoint.

Eastern Europe

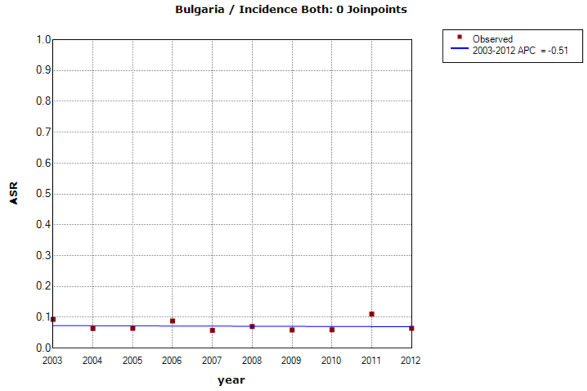

* Indicates that the Annual Percent Change (APC) is significantly different from zero at the alpha = 0.05 level
Final Selected Model: 0 Joinpoints.

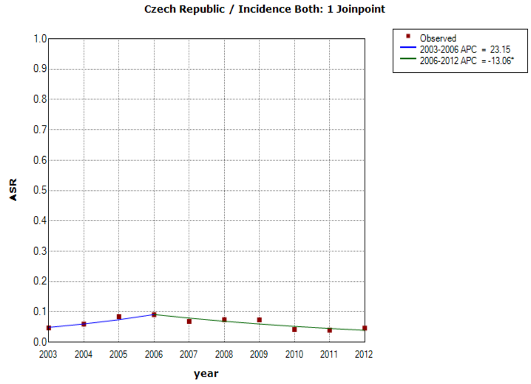

* Indicates that the Annual Percent Change (APC) is significantly different from zero at the alpha = 0.05 level
Final Selected Model: 1 Joinpoint.

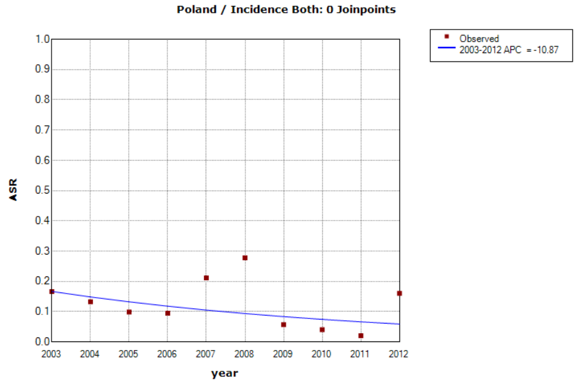

* Indicates that the Annual Percent Change (APC) is significantly different from zero at the alpha = 0.05 level
Final Selected Model: 0 Joinpoints.

Africa

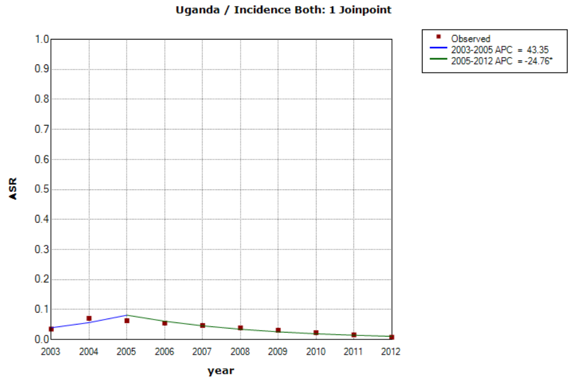

* Indicates that the Annual Percent Change (APC) is significantly different from zero at the alpha = 0.05 level
Final Selected Model: 1 Joinpoint.

d.) Young

Asia

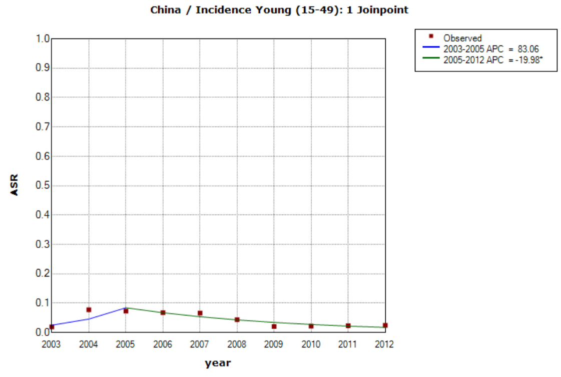

* Indicates that the Annual Percent Change (APC) is significantly different from zero at the alpha = 0.05 level
Final Selected Model: 1 Joinpoint.

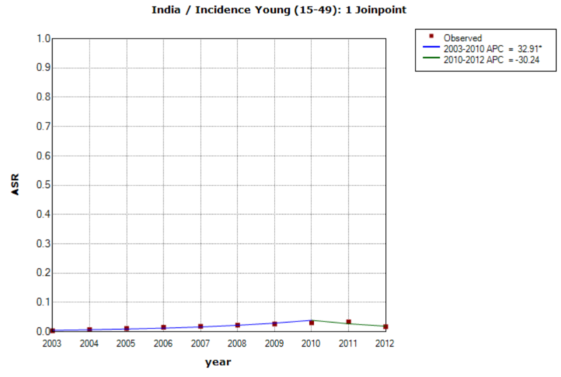

* Indicates that the Annual Percent Change (APC) is significantly different from zero at the alpha = 0.05 level
Final Selected Model: 1 Joinpoint.

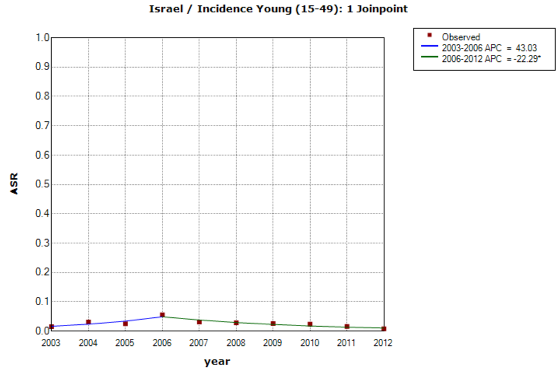

* Indicates that the Annual Percent Change (APC) is significantly different from zero at the alpha = 0.05 level
Final Selected Model: 1 Joinpoint.

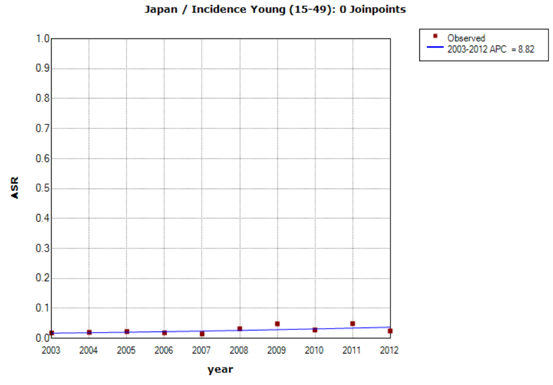

* Indicates that the Annual Percent Change (APC) is significantly different from zero at the alpha = 0.05 level
Final Selected Model: 0 Joinpoints.

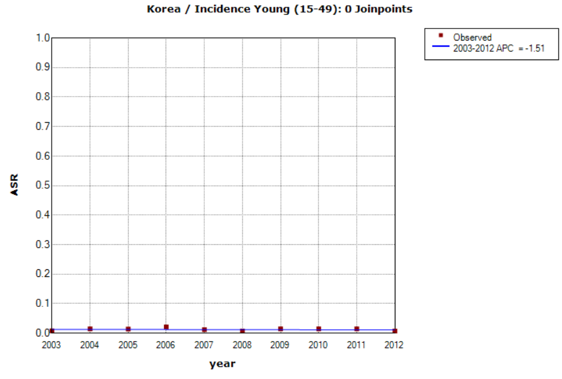

* Indicates that the Annual Percent Change (APC) is significantly different from zero at the alpha = 0.05 level
Final Selected Model: 0 Joinpoints.

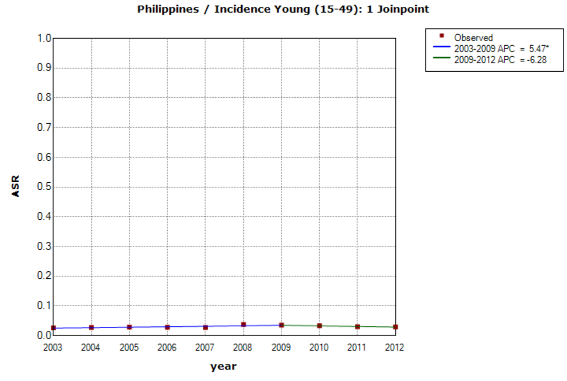

* Indicates that the Annual Percent Change (APC) is significantly different from zero at the alpha = 0.05 level
Final Selected Model: 1 Joinpoint.

Thailand / Incidence Young (15-49): 0 Joinpoints

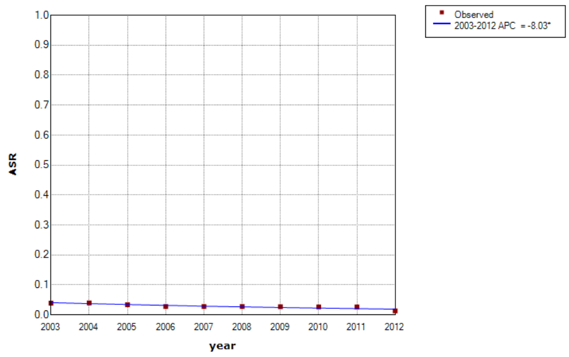

* Indicates that the Annual Percent Change (APC) is significantly different from zero at the alpha = 0.05 level
Final Selected Model: 0 Joinpoints.

Turkey / Incidence Young (15-49): 1 Joinpoint

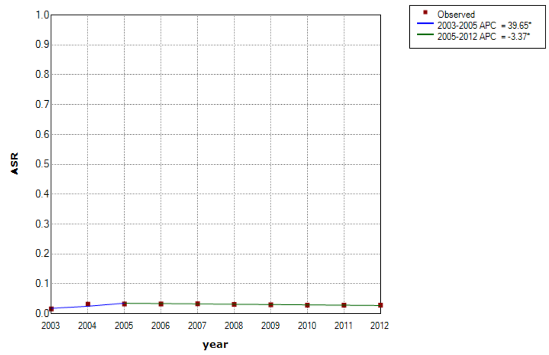

* Indicates that the Annual Percent Change (APC) is significantly different from zero at the alpha = 0.05 level
Final Selected Model: 1 Joinpoint.

Oceania

Australia / Incidence Young (15-49): 1 Joinpoint

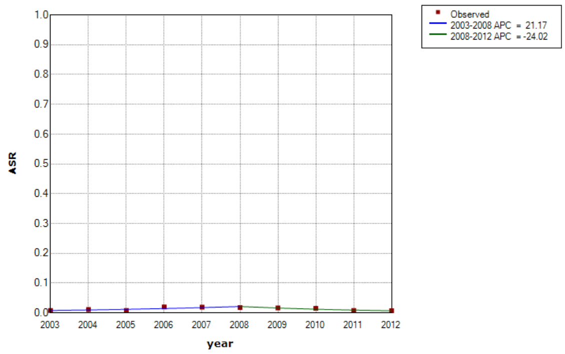

* Indicates that the Annual Percent Change (APC) is significantly different from zero at the alpha = 0.05 level
Final Selected Model: 1 Joinpoint.

New Zealand / Incidence Young (15-49): 1 Joinpoint

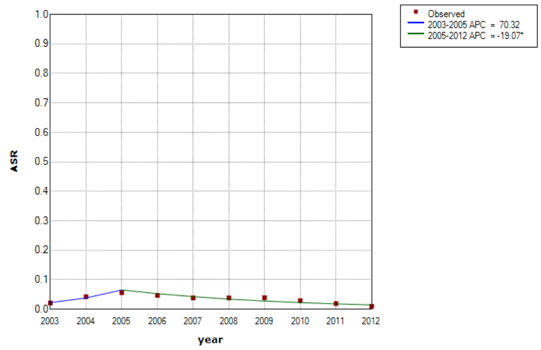

* Indicates that the Annual Percent Change (APC) is significantly different from zero at the alpha = 0.05 level
Final Selected Model: 1 Joinpoint.

Northern America

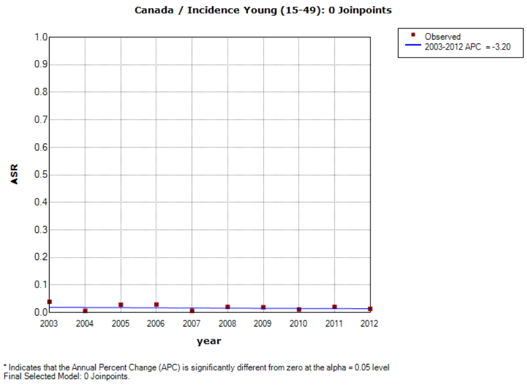

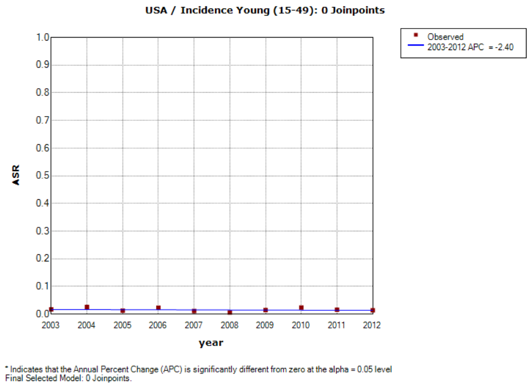

Southern America

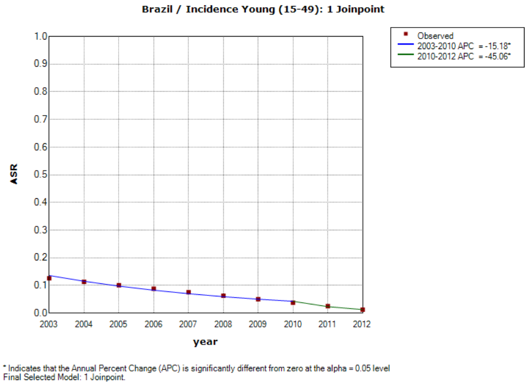

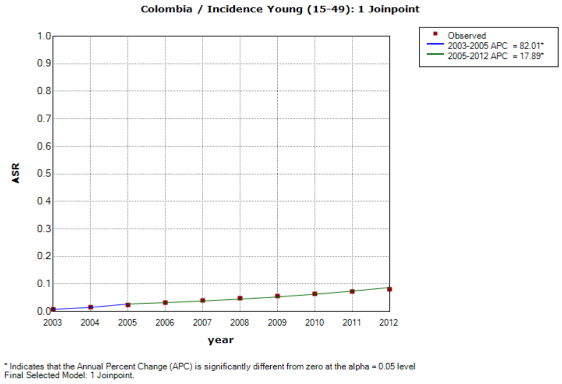

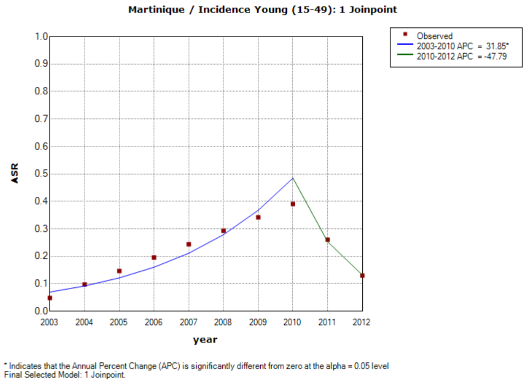

Northern Europe

Denmark / Incidence Young (15-49): 0 Joinpoints

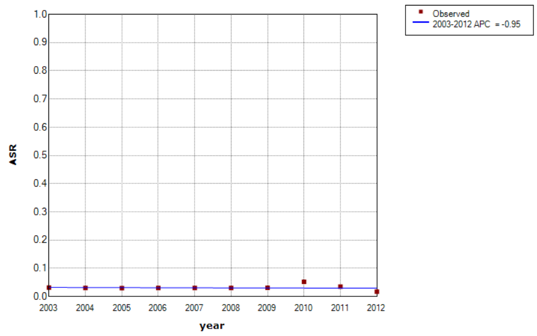

* Indicates that the Annual Percent Change (APC) is significantly different from zero at the alpha = 0.05 level
Final Selected Model: 0 Joinpoints.

Estonia / Incidence Young (15-49): 1 Joinpoint

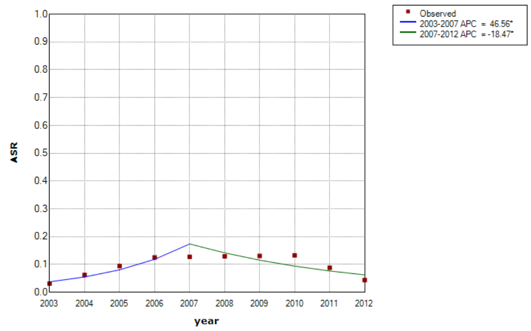

* Indicates that the Annual Percent Change (APC) is significantly different from zero at the alpha = 0.05 level
Final Selected Model: 1 Joinpoint.

Iceland / Incidence Young (15-49): 1 Joinpoint

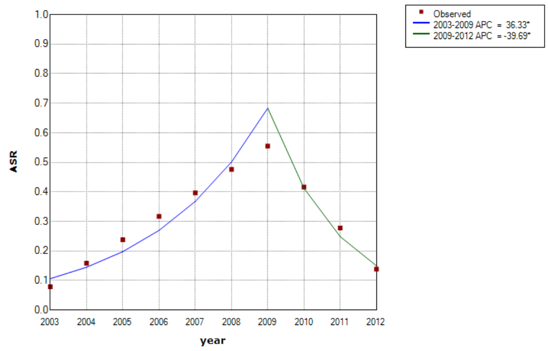

* Indicates that the Annual Percent Change (APC) is significantly different from zero at the alpha = 0.05 level
Final Selected Model: 1 Joinpoint.

Ireland / Incidence Young (15-49): 1 Joinpoint

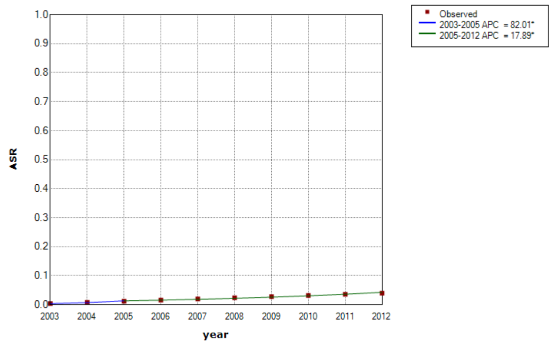

* Indicates that the Annual Percent Change (APC) is significantly different from zero at the alpha = 0.05 level
Final Selected Model: 1 Joinpoint.

Lithuania / Incidence Young (15-49): 1 Joinpoint

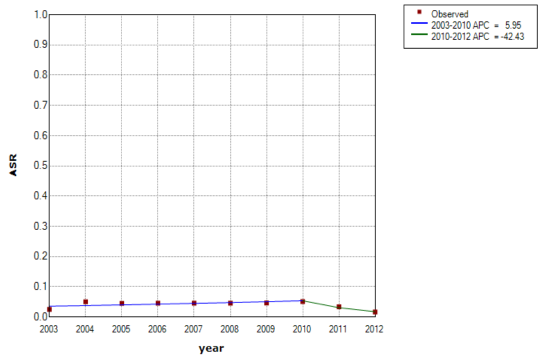

* Indicates that the Annual Percent Change (APC) is significantly different from zero at the alpha = 0.05 level
Final Selected Model: 1 Joinpoint.

Norway / Incidence Young (15-49): 1 Joinpoint

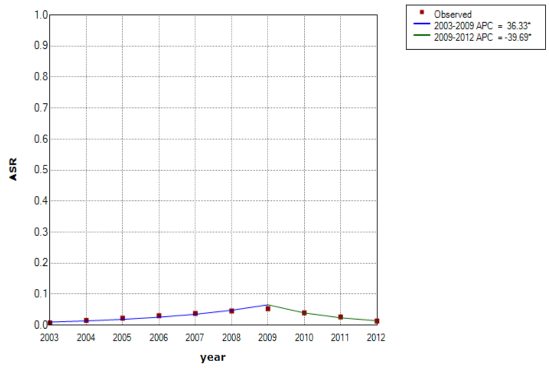

* Indicates that the Annual Percent Change (APC) is significantly different from zero at the alpha = 0.05 level
Final Selected Model: 1 Joinpoint.

United Kingdom / Incidence Young (15-49): 0 Joinpoints

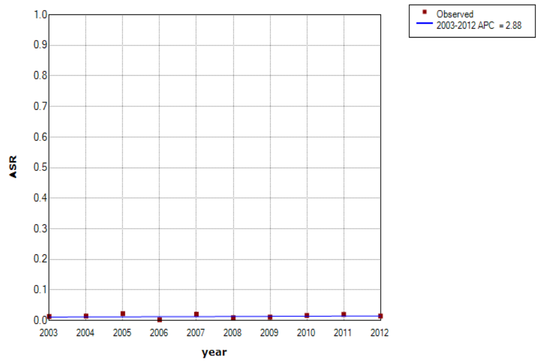

* Indicates that the Annual Percent Change (APC) is significantly different from zero at the alpha = 0.05 level
Final Selected Model: 0 Joinpoints.

Western Europe

Austria / Incidence Young (15-49): 0 Joinpoints

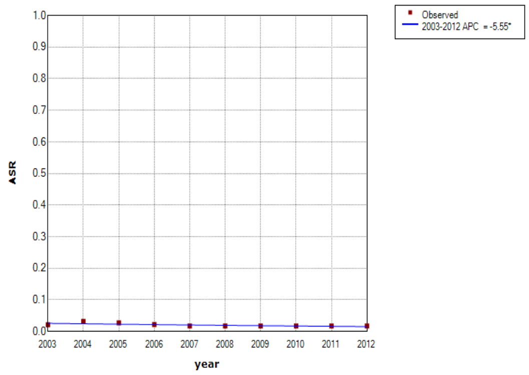

* Indicates that the Annual Percent Change (APC) is significantly different from zero at the alpha = 0.05 level
Final Selected Model: 0 Joinpoints.

France / Incidence Young (15-49): 0 Joinpoints

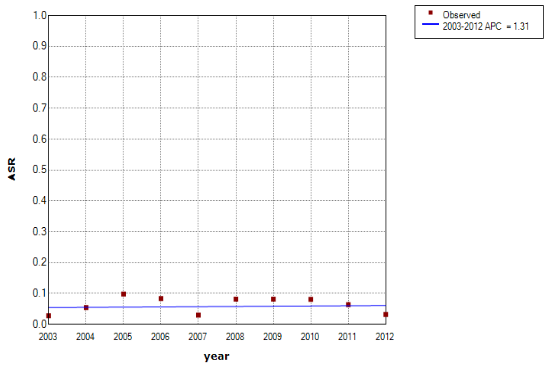

* Indicates that the Annual Percent Change (APC) is significantly different from zero at the alpha = 0.05 level
Final Selected Model: 0 Joinpoints.

Germany / Incidence Young (15-49): 1 Joinpoint

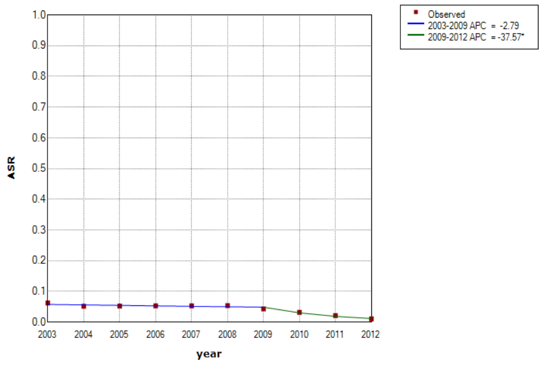

* Indicates that the Annual Percent Change (APC) is significantly different from zero at the alpha = 0.05 level
Final Selected Model: 1 Joinpoint.

Netherlands / Incidence Young (15-49): 0 Joinpoints

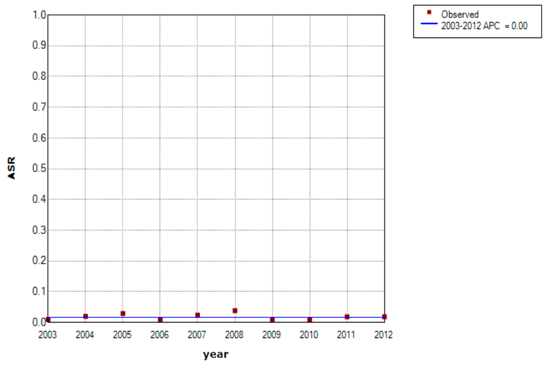

* Indicates that the Annual Percent Change (APC) is significantly different from zero at the alpha = 0.05 level
Final Selected Model: 0 Joinpoints.

Switzerland / Incidence Young (15-49): 0 Joinpoints

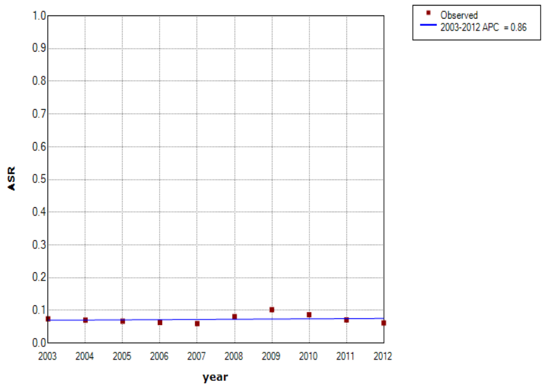

* Indicates that the Annual Percent Change (APC) is significantly different from zero at the alpha = 0.05 level
Final Selected Model: 0 Joinpoints.

Southern Europe

Croatia / Incidence Young (15-49): 0 Joinpoints

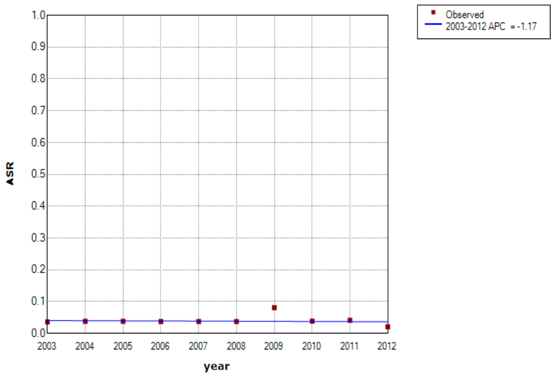

* Indicates that the Annual Percent Change (APC) is significantly different from zero at the alpha = 0.05 level
Final Selected Model: 0 Joinpoints.

Italy / Incidence Young (15-49): 0 Joinpoints

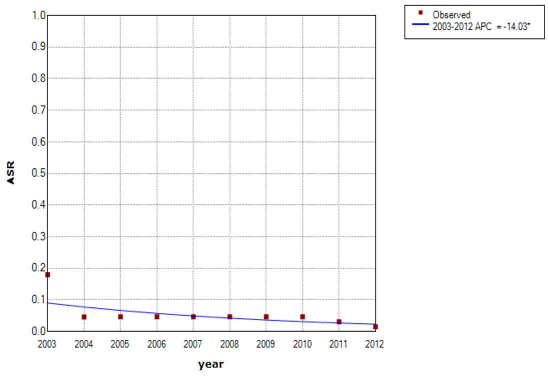

* Indicates that the Annual Percent Change (APC) is significantly different from zero at the alpha = 0.05 level
Final Selected Model: 0 Joinpoints.

Malta / Incidence Young (15-49): 1 Joinpoint

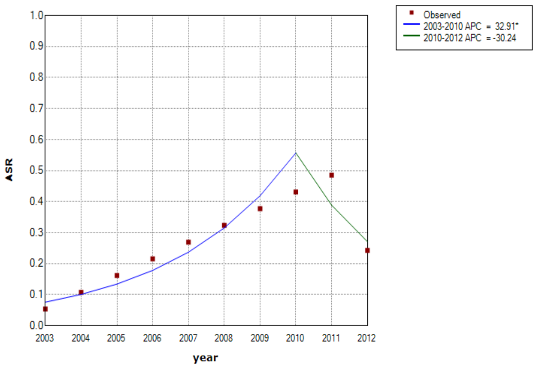

* Indicates that the Annual Percent Change (APC) is significantly different from zero at the alpha = 0.05 level
Final Selected Model: 1 Joinpoint.

Slovenia / Incidence Young (15-49): 1 Joinpoint

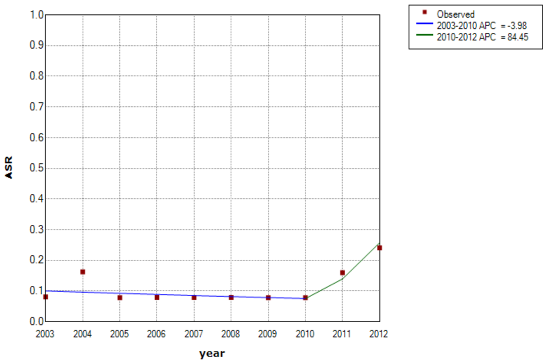

* Indicates that the Annual Percent Change (APC) is significantly different from zero at the alpha = 0.05 level
Final Selected Model: 1 Joinpoint.

Spain / Incidence Young (15-49): 1 Joinpoint

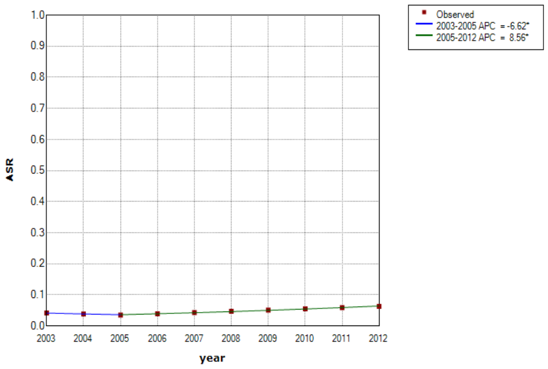

* Indicates that the Annual Percent Change (APC) is significantly different from zero at the alpha = 0.05 level
Final Selected Model: 1 Joinpoint.

Eastern Europe

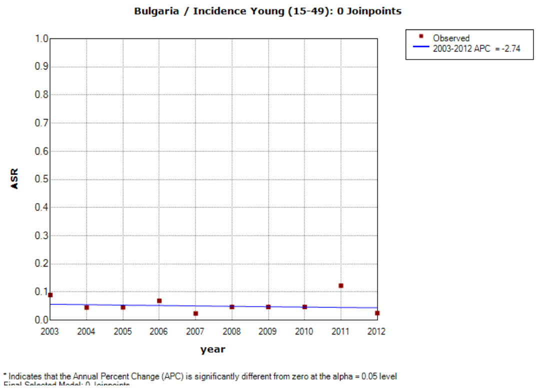

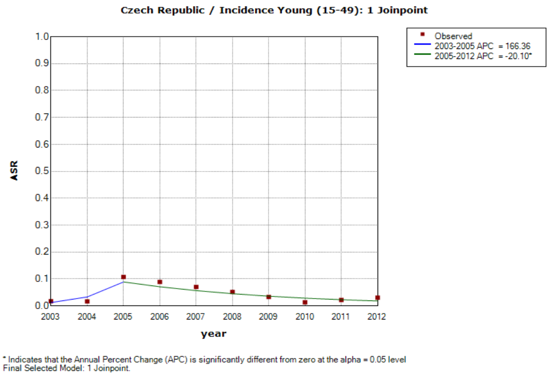

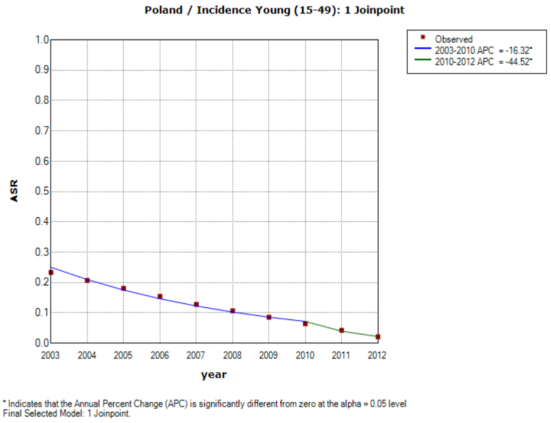

Africa

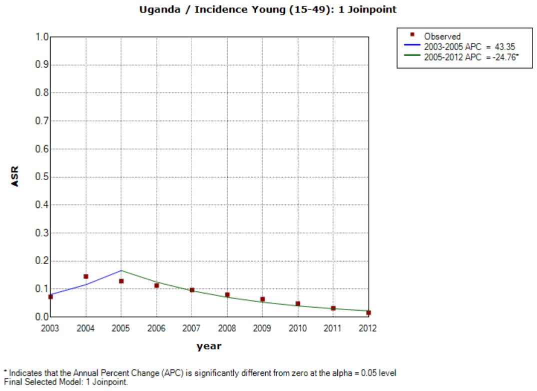

e.) Old

Asia

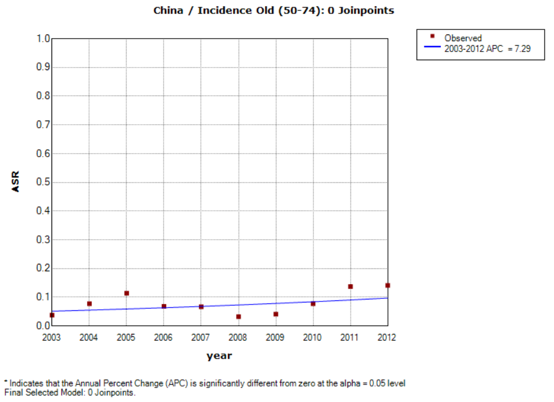
China / Incidence Old (50-74): 0 Joinpoints
Observed
2003-2012 APC = 7.29
ASR
year
* Indicates that the Annual Percent Change (APC) is significantly different from zero at the alpha = 0.05 level
Final Selected Model: 0 Joinpoints.

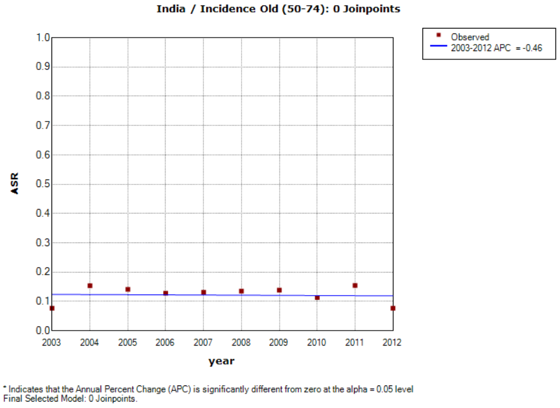
India / Incidence Old (50-74): 0 Joinpoints
Observed
2003-2012 APC = -0.46
ASR
year
* Indicates that the Annual Percent Change (APC) is significantly different from zero at the alpha = 0.05 level
Final Selected Model: 0 Joinpoints.

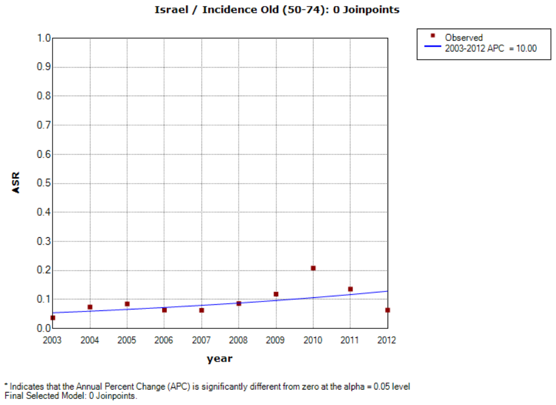
Israel / Incidence Old (50-74): 0 Joinpoints
Observed
2003-2012 APC = 10.00
ASR
year
* Indicates that the Annual Percent Change (APC) is significantly different from zero at the alpha = 0.05 level
Final Selected Model: 0 Joinpoints.

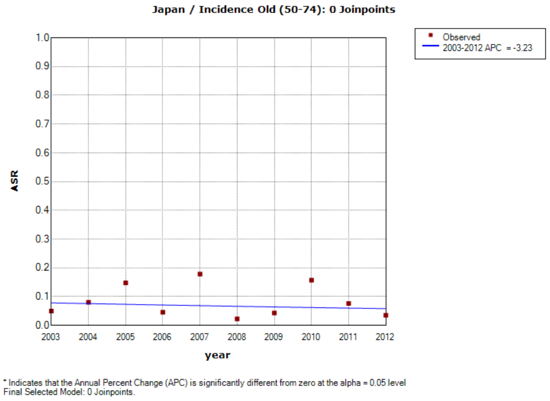
Japan / Incidence Old (50-74): 0 Joinpoints
Observed
2003-2012 APC = -3.23
ASR
year
* Indicates that the Annual Percent Change (APC) is significantly different from zero at the alpha = 0.05 level
Final Selected Model: 0 Joinpoints.

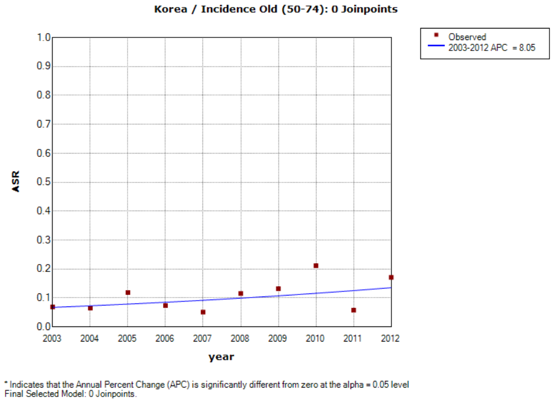
Korea / Incidence Old (50-74): 0 Joinpoints
Observed
2003-2012 APC = 8.05
ASR
year
* Indicates that the Annual Percent Change (APC) is significantly different from zero at the alpha = 0.05 level
Final Selected Model: 0 Joinpoints.

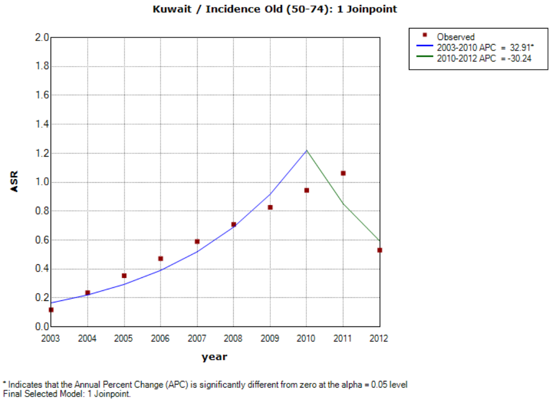
Kuwait / Incidence Old (50-74): 1 Joinpoint
Observed
2003-2010 APC = 32.91*
2010-2012 APC = -30.24
ASR
year
* Indicates that the Annual Percent Change (APC) is significantly different from zero at the alpha = 0.05 level
Final Selected Model: 1 Joinpoint.

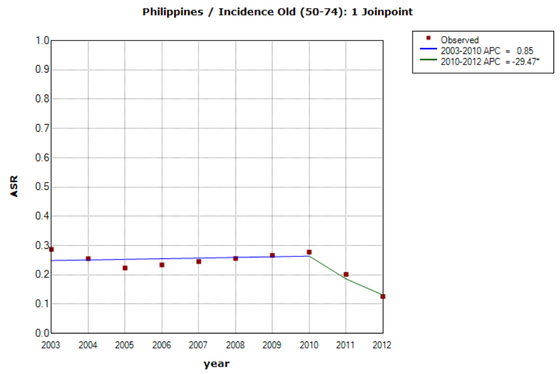

* Indicates that the Annual Percent Change (APC) is significantly different from zero at the alpha = 0.05 level
Final Selected Model: 1 Joinpoint.

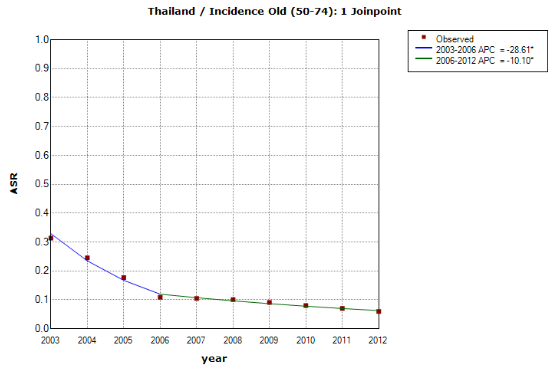

* Indicates that the Annual Percent Change (APC) is significantly different from zero at the alpha = 0.05 level
Final Selected Model: 1 Joinpoint.

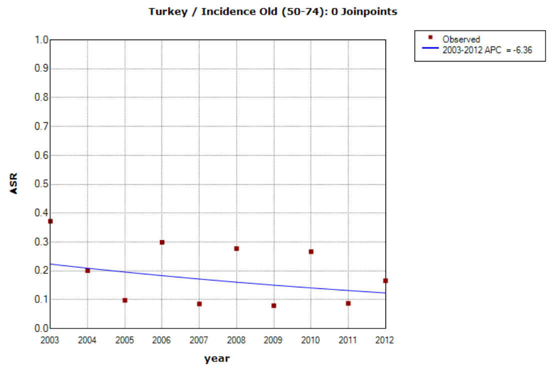

* Indicates that the Annual Percent Change (APC) is significantly different from zero at the alpha = 0.05 level
Final Selected Model: 0 Joinpoints.

Oceania

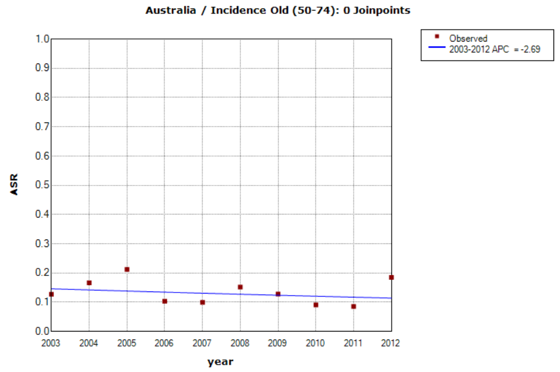

* Indicates that the Annual Percent Change (APC) is significantly different from zero at the alpha = 0.05 level
Final Selected Model: 0 Joinpoints.

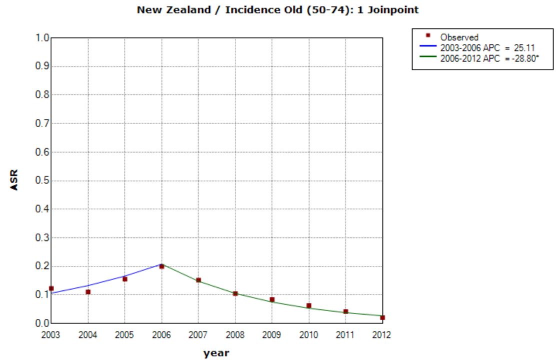

* Indicates that the Annual Percent Change (APC) is significantly different from zero at the alpha = 0.05 level
Final Selected Model: 1 Joinpoint.

Northern America

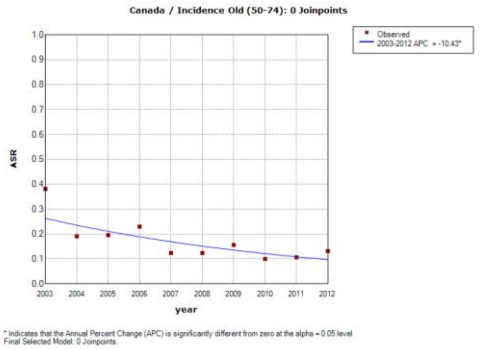

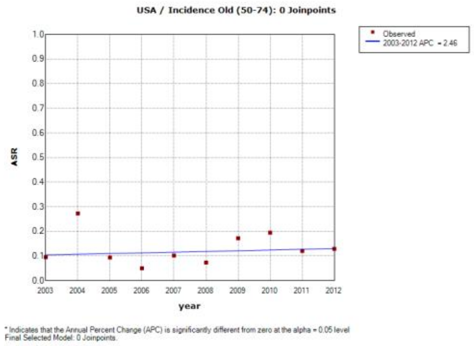

Southern America

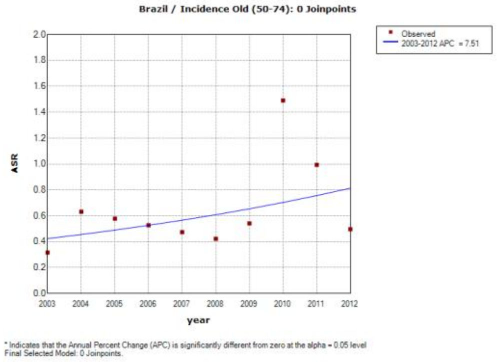

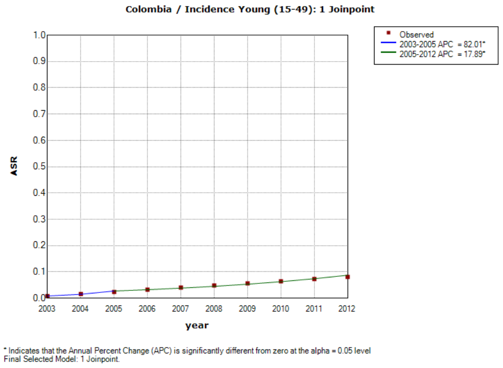

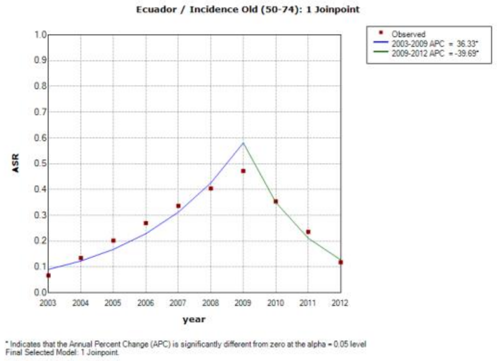

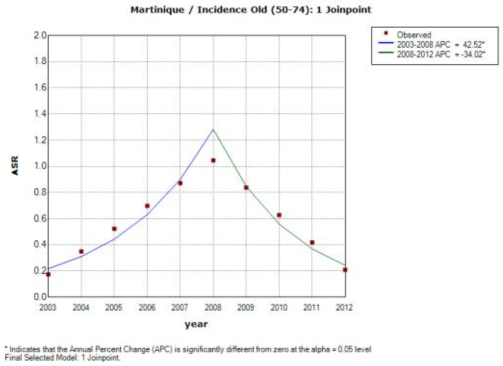

Northern Europe

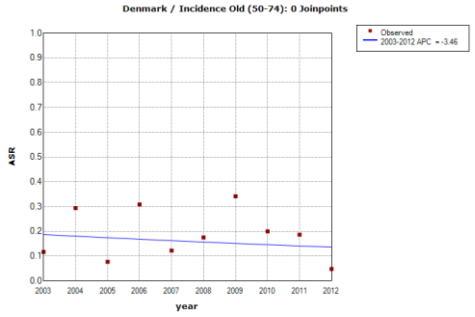

* Indicates that the Annual Percent Change (APC) is significantly different from zero at the alpha = 0.05 level
Final Selected Model: 0 Joinpoints.

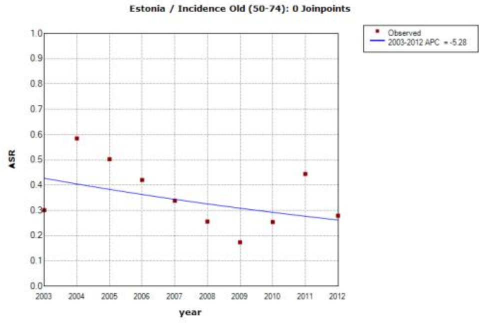

* Indicates that the Annual Percent Change (APC) is significantly different from zero at the alpha = 0.05 level
Final Selected Model: 0 Joinpoints.

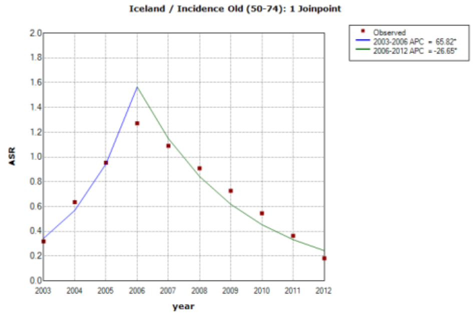

* Indicates that the Annual Percent Change (APC) is significantly different from zero at the alpha = 0.05 level
Final Selected Model: 1 Joinpoint.

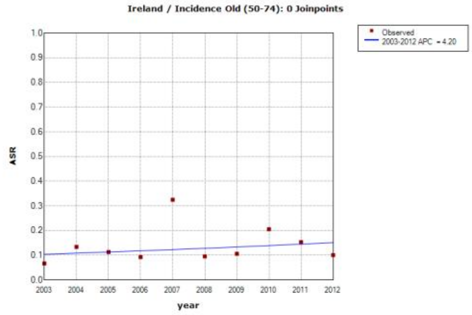

* Indicates that the Annual Percent Change (APC) is significantly different from zero at the alpha = 0.05 level
Final Selected Model: 0 Joinpoints.

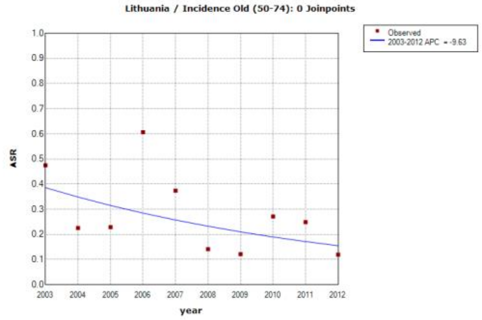

* Indicates that the Annual Percent Change (APC) is significantly different from zero at the alpha = 0.05 level
Final Selected Model: 0 Joinpoints.

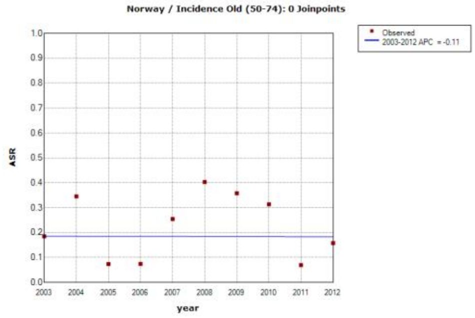

* Indicates that the Annual Percent Change (APC) is significantly different from zero at the alpha = 0.05 level
Final Selected Model: 0 Joinpoints.

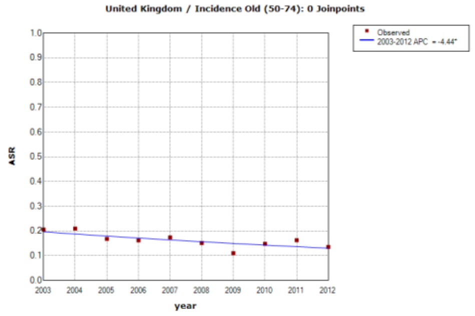

* Indicates that the Annual Percent Change (APC) is significantly different from zero at the alpha = 0.05 level
Final Selected Model: 0 Joinpoints.

Western Europe

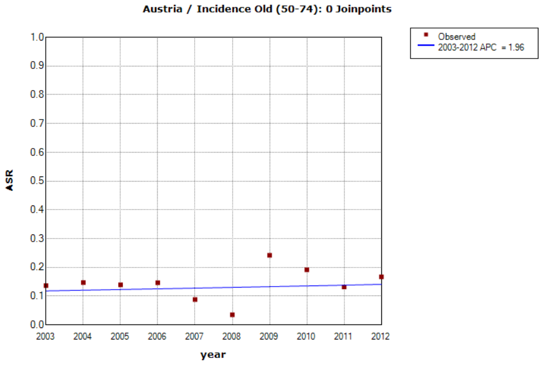

* Indicates that the Annual Percent Change (APC) is significantly different from zero at the alpha = 0.05 level
Final Selected Model: 0 Joinpoints.

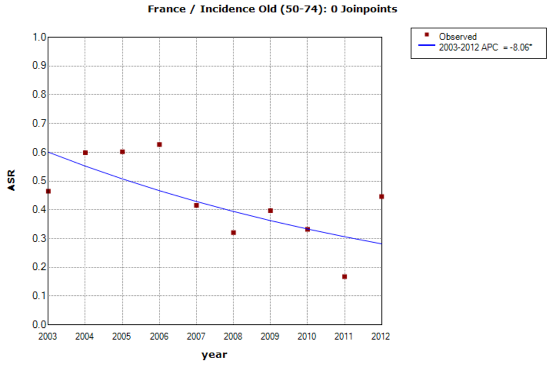

* Indicates that the Annual Percent Change (APC) is significantly different from zero at the alpha = 0.05 level
Final Selected Model: 0 Joinpoints.

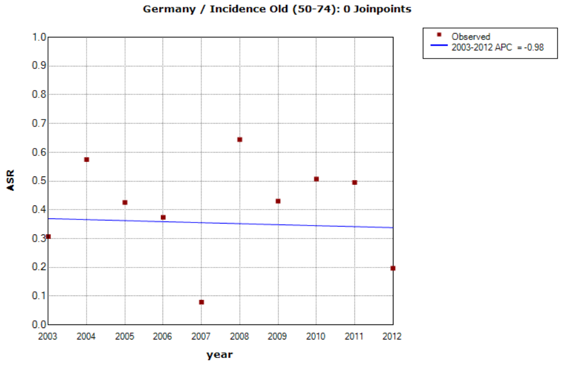

* Indicates that the Annual Percent Change (APC) is significantly different from zero at the alpha = 0.05 level
Final Selected Model: 0 Joinpoints.

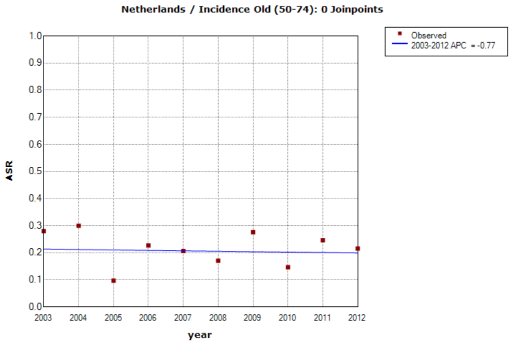

* Indicates that the Annual Percent Change (APC) is significantly different from zero at the alpha = 0.05 level
Final Selected Model: 0 Joinpoints.

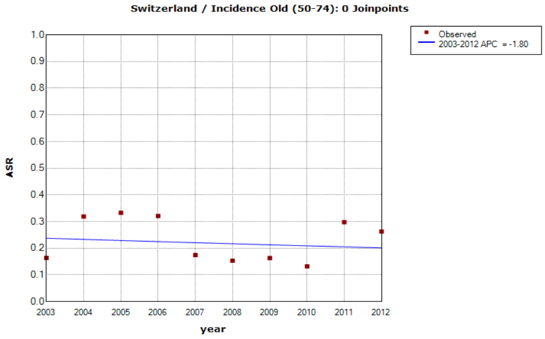

* Indicates that the Annual Percent Change (APC) is significantly different from zero at the alpha = 0.05 level
Final Selected Model: 0 Joinpoints.

Southern Europe

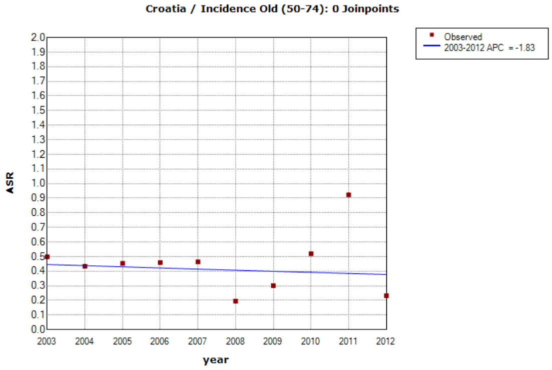

* Indicates that the Annual Percent Change (APC) is significantly different from zero at the alpha = 0.05 level
Final Selected Model: 0 Joinpoints.

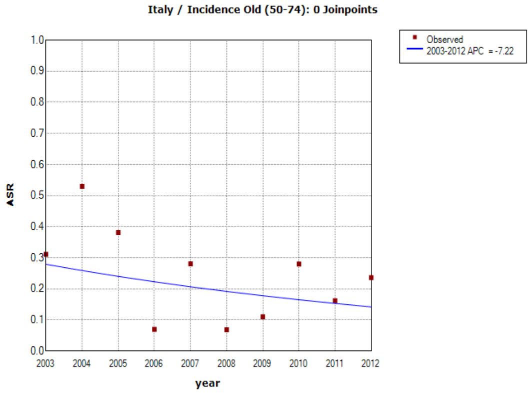

* Indicates that the Annual Percent Change (APC) is significantly different from zero at the alpha = 0.05 level
Final Selected Model: 0 Joinpoints.

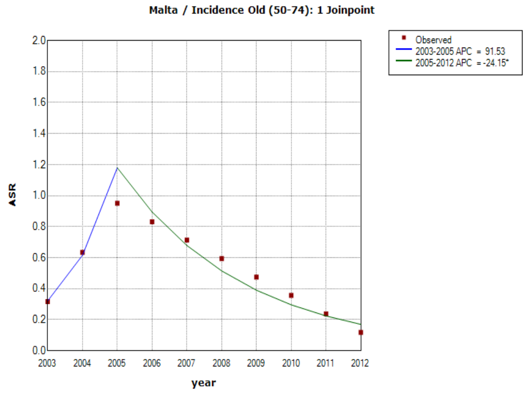

* Indicates that the Annual Percent Change (APC) is significantly different from zero at the alpha = 0.05 level
Final Selected Model: 1 Joinpoint.

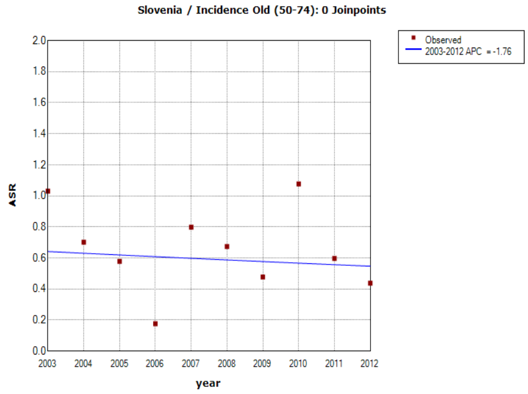

* Indicates that the Annual Percent Change (APC) is significantly different from zero at the alpha = 0.05 level
Final Selected Model: 0 Joinpoints.

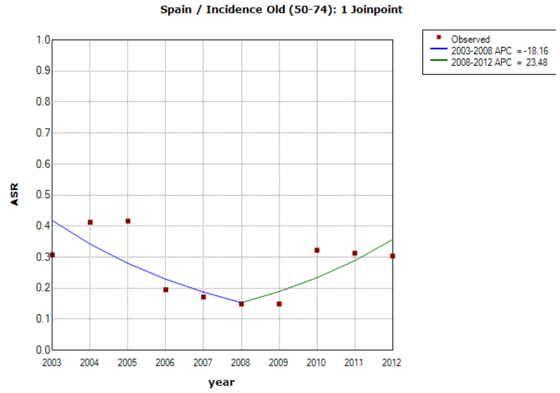

* Indicates that the Annual Percent Change (APC) is significantly different from zero at the alpha = 0.05 level
Final Selected Model: 1 Joinpoint.

Eastern Europe

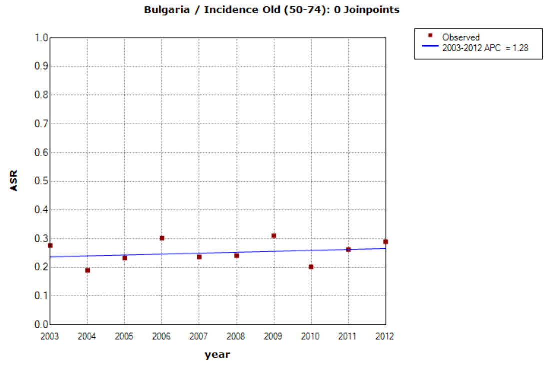

* Indicates that the Annual Percent Change (APC) is significantly different from zero at the alpha = 0.05 level
Final Selected Model: 0 Joinpoints.

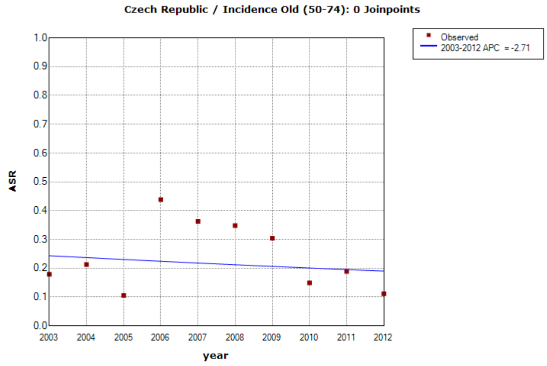

* Indicates that the Annual Percent Change (APC) is significantly different from zero at the alpha = 0.05 level
Final Selected Model: 0 Joinpoints.

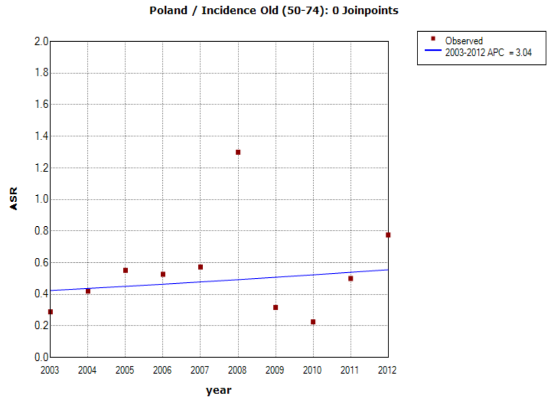

* Indicates that the Annual Percent Change (APC) is significantly different from zero at the alpha = 0.05 level
Final Selected Model: 0 Joinpoints.
